# Scoping review on the effect of labour inspections on occupational health and safety: a meta-analytic update

**DOI:** 10.1186/s12995-026-00497-6

**Published:** 2026-02-12

**Authors:** Melanie Schubert, Ulrich Bolm-Audorff, Johan Hviid Andersen, Gabriela Petereit-Haack, David Reissig, Andreas Seidler

**Affiliations:** 1 Institute and Policlinic of Occupational and Social Medicine, Faculty of Medicine, Technische Universität Dresden, Dresden, Germany; 2 Department of Occupational Medicine, Danish Ramazzini Centre, Regional Hospital West Jutland – University Clinic, Herning, Denmark; 3 Division of Occupational Health, Department of Occupational Safety, Regional Government of South Hesse, Wiesbaden, Germany

**Keywords:** Compliance, Enforcement, Injuries, Inspections, Labour, Meta-analysis, Occupational safety and health, OSH, Scoping review, Work

## Abstract

**Purpose:**

The purpose of this scoping review was to summarise recent literature on labour inspections carried out by government authorities and accident insurance organisations, and their impact on workers’ health and safety.

**Methods:**

A scoping review was conducted to investigate the effect of occupational safety and health (OSH) inspectorate interventions on occupational safety and health. The methodical approach was based on a previous systematic review on the effect of inspections on OSH. Literature searches were performed in PubMed, Web of Science and NIOSHTIC-2, complemented by screening of reference lists of the included full texts and topic-relevant reviews and a forward search in Google Scholar. Additionally, OSH authorities and accident insurance institutions in Germany, as well as the EU-OSHA national focal points were asked to submit reports on OSH inspections. Furthermore, the ‘grey’ literature search was extended to publications from the federal governments in Germany, Austria and Switzerland. Findings were extracted and combined with studies from the above-mentioned systematic review for meta-analysis. The study protocol with the methodological procedure was registered on Open Science Framework (https://osf.io/gy2u6/).

**Results:**

A total of 37 publications from Europe and North America on the effect of OSH inspections by government authorities were included in the study. Most of the studies examined the effect of labour inspections on (fatal and non-fatal) injuries at work, as well as the implementation of safety measures and compliance with legal requirements. The results of the individual studies generally indicate a protective effect of labour inspections on work-related injuries and compliance, but the results regarding compensation claims were heterogenous. In addition, pooled risk estimates from meta-analyses indicate that inspected workplaces had a lower risk of work-related injuries (relative risk (RR) = 0.75, 95% CI 0.57–0.97) and of non-compliance with workplace safety rules (RR = 0.68, 95% CI 0.45–1.01) compared with non-inspected workplaces.

**Conclusions:**

Our results reinforce the findings of previous reviews that inspections are effective in reducing work-related injuries. Thus, inspections are important for promoting and improving occupational health and safety.

**Supplementary Information:**

The online version contains supplementary material available at 10.1186/s12995-026-00497-6.

## Background

Every day, many workers face high risks to their health and safety at work. The International Labour Organization (ILO) estimated that 395 million workers sustain non-fatal injuries, and nearly 3 million people die from work-related accidents and diseases each year [1]. The economic burden is high comprising around 4% of global Gross Domestic Product [2]. According to the 2025 report by the American Federation of Labour – Congress of Industrial Organisations (AFL-CIO), 385 workers died each day because of hazardous conditions at work, 5,283 workers lost their lives on the job and 135,304 deaths attributed to occupational diseases in the US in 2023 [3]. The social and economic costs of work-related injuries and illnesses are high, estimated at between $174 billion and $348 billion per year [3].

In industrialised countries, occupational health and safety organisations form a fundamental part of modern society. Efforts have been made to protect workers’ health and safety by introducing regulations including enforcement and work-specific programmes. For example in the US, the number of work-related injuries and deaths have been decreasing since the 1970s with introduction of the Occupational Safety and Health Administration (OSHA) and the National Institute for Occupational Safety and Health (NIOSH) aiming “to ensure every man and woman in the Nation safe and healthful working conditions and to preserve our human resources” [4].

A slight increase in work-related fatalities was observed during the 2019–2022 period of the COVID pandemic. Since its introduction, the OSH Act has saved more than 712,000 lives and reduced workplace deaths by almost two-thirds, despite the workforce doubling in size [3]. Though, changes in the structure of work, including a shift towards less hazardous industries [5] as well as structural changes such as mechanization and automation [6] are also associated with declines in work-related injuries and deaths. Similarly, the number of fatal work-related accidents in Germany fell from 2,696 in 1970 to 351 in 2023 [7]. In 1970, there were 0.072 fatal work-related accidents per 1 million working hours, compared to 0.007 in 2023. According to this report, 72,203 cases of occupational disease were recognised in 2023, compared to 10,384 in 1990. The number of deaths resulting from occupational diseases increased from 1,440 in 1990 to 2,151 in 2023 [7]. This is primarily due to the sharp rise in the number of occupational infectious diseases as a result of the COVID-19 pandemic.

Regulations can be enforced through inspections carried out by government authorities. Literature-based studies indicate a protective influence of inspections on work-related injuries and compliance with occupational health and safety regulations [8–11]. This appears to be due in particular to the “deterrent” effect of inspections with possible subsequent sanctions [8, 11]. There is also evidence that visits reduce the number of injuries in the long term, but not in the short term [10]. The type of inspection also appears to have an impact on occupational safety: Mischke et al. [10] found evidence that focused inspections – i.e., inspections that are more targeted and concentrate on specific aspects – have a protective effect on occupational safety when compared with general inspections, which are broader in scope. There is also evidence that the first visit has the greatest impact on compliance [8, 11]. Recently, Bondebjerg et al. [9] used evidence mapping to compile an up-to-date overview of existing systematic reviews and primary studies that examined the effects of measures taken by government authorities on occupational health and safety. Based on these studies, they concluded that inspections by the government inspectorate have an effect on the frequency of injuries and compliance, as well as on information, guidance and consultation.

In some European countries, such as Germany, Austria and Switzerland, inspections may also be carried out by accident insurance providers. To date, scientific research has only focused on inspections carried out by government authorities. Thus, there are still research gaps regarding non-governmental occupational safety and health actors. This study aimed to review recent literature on inspections conducted by accident insurance organisations and their effects on workers’ health and safety. Occupational safety is constantly evolving, and workplace inspections are often influenced by staffing reductions. In this context, we also considered it valuable to update the evidence by including recently published literature on the effects of inspections conducted by governmental authorities. Thus, the aim was to provide an overview of the effects of both governmental inspections and inspections conducted by accident insurance providers on occupational safety and health. In line with previous research, this study focused on high-income countries to ensure comparability of the prevailing legal frameworks governing occupational safety [9]. Furthermore, the results of the literature search were quantitatively synthesised through meta-analysis alongside studies from the systematic review by Andersen et al. [3]. This was done to strengthen the scientific evidence on the effect of labour inspections on occupational safety and health.

## Materials and methodsresearch question and study eligibility

This study was conducted under Project F 2592, commissioned by the German Federal Institute for Occupational Safety and Health (BAuA), with the objective of conducting a scoping review to update the evidence on the effects of occupational safety and health (OSH) inspections. The main research question was: ”What is the effect of OSH inspectorate interventions on occupational safety and health in companies?“ The update was based on the systematic review by Andersen and colleagues [8] which summarizes the evidence on the effect of OSH interventions for the years 1966–2017. The research question was specified according to a modified PICO (population-intervention-comparison (context)-outcome) format by Andersen et al. [8] where the methodology is described in detail. In summary, the study population of our scoping review included the working population of countries with developed economies according to the country classification of the United Nations. Studies from developing and emerging countries were excluded due to a lack of comparability of the prevailing legal regulations on occupational safety. The key intervention were inspections by government authorities and accident insurance institutions. Outcomes included work-related injuries, accidents, occupational diseases, illnesses and complaints, as well as fatalities, sick leave and return-to-work. Furthermore, employee behaviour, improvements to working conditions (e.g. reducing exposure and introducing occupational safety measures) and the use of personal protective equipment (e.g. hearing protection, respirators and safety helmets) were also of interest. The following study types were included: epidemiological observational studies, intervention studies, and systematic reviews. Qualitative studies, case studies and case series were excluded. Furthermore, narrative reviews, selective reviews and scoping reviews were excluded from the review as well as editorials, comments, letters to the editors, and any publication with incomplete information on study methods and results. Since the systematic review by Andersen et al. covered the time from 1966 to February 2017, only studies published since January 2017 were included in this scoping review. The study protocol with the methodological procedure was registered a priori on Open Science Framework (https://osf.io/gy2u6/).

### Information sources and search

According to Andersen et al. (2019), the electronic databases PubMed, Web of Science (via Clarivate), and NIOSHTIC-2, a database provided by NIOSH) were included in the literature search. The search string of the literature search was based on the search by Andersen et al. [8]. The search string was validated a priori with nine key studies published after 2016. As a result, all key studies listed in PubMed and Web of Science were found using a modified search string. The search in NIOSHTIC-2 was conducted by a NIOSH staff member (Krysten North, MPH, Technical Information Specialist Science Applications Branch). The key studies and the (modified) search strings used in the scoping review are provided in the supplementary material.

Furthermore, a grey literature search was performed. Therefore, the reference lists of the included full texts and additional topic-relevant reviews [8, 9, 12] were screened (backward search). Moreover, a forward search was conducted in Google Scholar. In addition, the ministries responsible for occupational safety and health of the 16 federal German states, the German state occupational physicians of the federal states and the heads of the prevention services of the German accident insurance institutions were contacted for relevant reports. Furthermore, the International Labour Organisation (ILO), the European Agency for Safety and Health at Work (EU-OSHA) and EU-OSHA country-specific “focal points” of the EU member states, European Free Trade Association (EFTA) countries and candidates were contacted (*n* = 40) for relevant publications. Since the focus was on inspection of insurers, the grey literature search was extended to include relevant publications from the occupational safety and health supervisory authorities of Austria and Switzerland that have a similar inspection system.

### Study selection and data collection

Results of the literature search were imported into an Endnote reference management system database. Duplicates references were removed at import automatically and afterwards manually. The literature selection was standardised according to pre-defined criteria in a title-abstract and full-text screening with a preceding pilot phase. The literature review was mainly carried out by one person; a second person independently screened 20% of the title abstracts and full texts. The results were compared and showed substantial agreement, with a Cohen’s kappa of 0.78 [13]. All disagreements regarding inclusion were resolved through discussion between the two reviewers. Further, in the event of uncertainties in the decision on inclusion or exclusion, the second person also checked the corresponding abstracts and full texts. The decisions of the two persons were compared with each other. Any discrepancies in the assessments were discussed and documented. For excluded full texts, reasons for exclusion were documented.

Data extraction into standardised tables was done by one person; a second person checked 20% of the data extractions for completeness and plausibility. The following information was extracted: general study characteristics (study name, country, study design, and study period); study population (recruitment method or data source, inclusion criteria, number of companies invited and participating, response rate, and occupational groups considered); intervention characteristics (description of inspections, study groups, and timing of measurements); outcome information (outcome name, definition, assessment method, and timing of measurements); results (statistical methods used, description of model or tests, and estimates); and other information (funding sources, conflicts of interest, and additional comments). The data extraction table was piloted in advance.

### Study selection

**Fig. 1:**
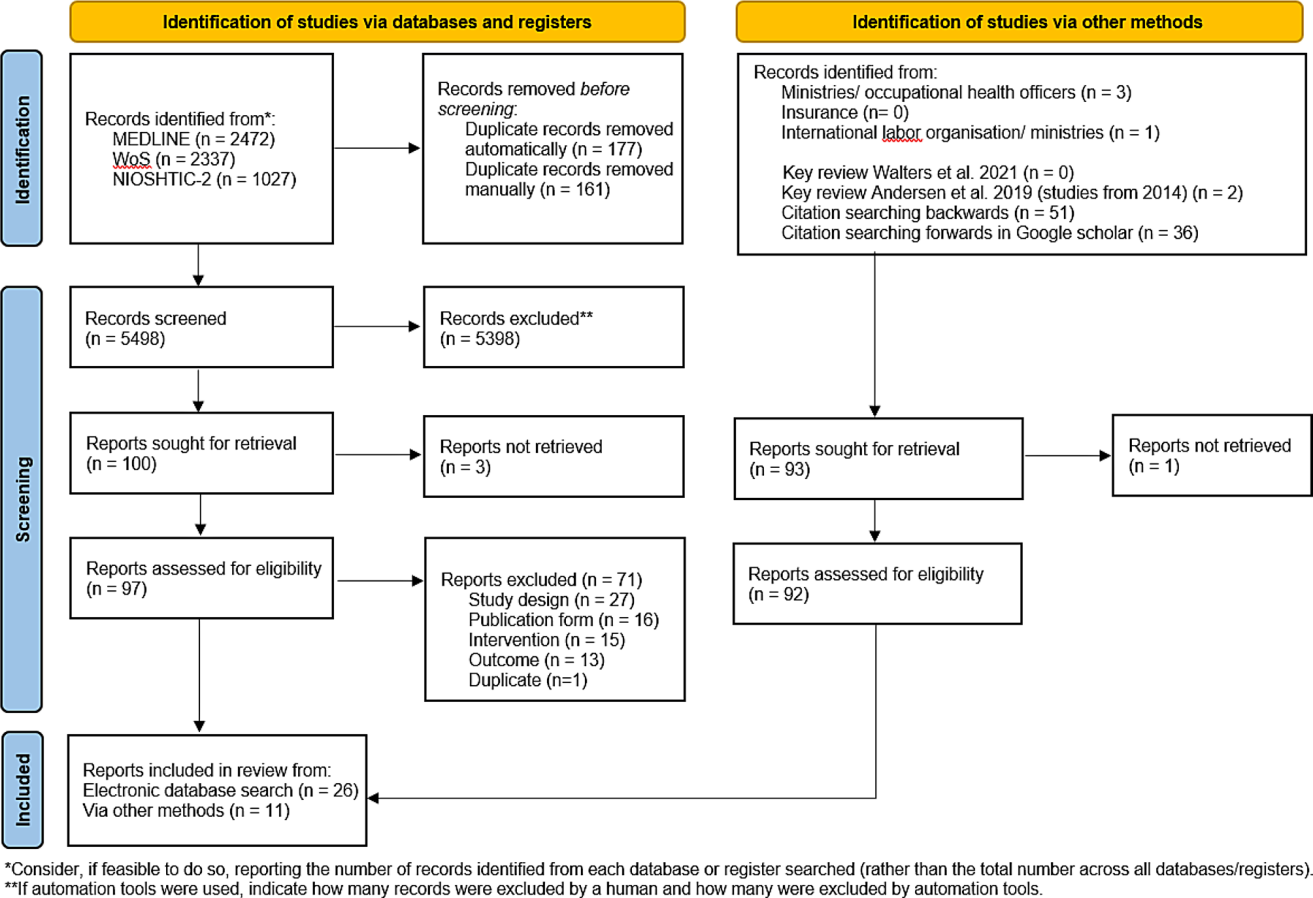
PRISMA 2020 flow diagram of literature search

After removal of duplicates, a total of 5498 publications from the electronic database search were included in the title-abstract review and screened for eligibility (search date to October 2, 2024 for PubMed/Web of Science or August 19, 2024 for NIOSHTIC-2 database). The full-text screening included 97 available articles from the electronic database search. A total of 26 articles from electronic database search were included in the scoping review. Most publications were excluded because the requirements for the inclusion criteria regarding study design (*n* = 27), publication form (*n* = 16), intervention (*n* = 15), or outcome (*n* = 13) were not met. In addition, 92 publications found through other methods, such as forward and backward search, were checked for inclusion and exclusion criteria of which 11 articles were included. Altogether, we identified and extracted 37 articles, i.e. 31 publications on epidemiological studies and six publications on systematic reviews. The PRISMA flow chart of literature search is shown in Fig. 1. Full data extraction tables of included studies are shown in the supplementary material.

### Statistical analysis

A descriptive overview is provided (see also data extraction tables and final report). Scoping reviews usually do not include meta-analyses; however, they are not limited to this aspect [14, 15]. The aim was to summarise the findings of the studies included in the scoping review by meta-analysis. Therefore, studies from the scoping review were combined with the studies included in the systematic review by Andersen et al. [8]. The suitability of the publications for meta-analysis was checked. In cases where different publications used the same database and time period to investigate the effect of inspections on the same outcome, the publication that was either the most comprehensive or the most recent was chosen for the meta-analysis. Thus, the number of studies that was included in the meta-analysis was lower than the number originally included in the meta-analysis by Andersen et al. [8]. This was done to avoid duplicate data in the meta-analysis. We used the outcome and intervention definition as well as the calculated risk estimates (odds ratios (OR)) of the individual studies as published by Andersen et al. [8]. In accordance with Andersen et al. [8], a meta-analysis was performed for the outcomes injuries and compliance. We did not run a meta-analysis for compensation claims. This is because studies used (acute) work accidents but also occupational diseases and disorders (which are characterized by different latency periods).

To quantitatively synthesize the effect estimates of inspections on the risk of work-related injuries and non-compliance, meta-analyses were conducted using random-effects models. Depending on the structure of the data, either two- or three-level meta-analyses were applied. Three-level models were used when multiple effect sizes were reported within a single study, for example, for different subgroups (e.g., injured workers vs. their co-workers) or divergent outcome definitions (e.g., injuries related to OSHA standards vs. injuries not attributable to OSHA standards). In such cases, correlated effect sizes may occur due to shared control groups, which can be adequately accounted for by using a three-level model. This approach allows for the separate estimation of variance within and between studies, leading to more robust estimates of the overall effect and heterogeneity measures [16, 17]. In the analysis relative risks (RR) and 95% confidence intervals were calculated. All analyses were conducted using the metafor package in the software R statistics [18–20], version 2025.05.0 Build 496, applying the restricted maximum likelihood (REML) method.

## Results

Reports on inspections conducted by accident insurance institutions were not provided upon request for data protection reasons. The results of the studies described here pertain exclusively to inspections carried out by government authorities. A comprehensive description of the studies is provided in the final report (available in German at https://www.baua.de/DE/Angebote/Publikationen/Berichte/F2592.) Detailed information is also presented in the data extraction tables in the supplementary materials.

### Systematic reviews

We included six publications on four systematic reviews [8, 9, 21–24]. One systematic review [21, 22] focussed on construction work, while the others had no limitation in occupations considered.

The two systematic reviews that performed a meta-analysis generally found an effect of inspections on occupational health and safety [8, 24]. The results of the meta-analysis by Andersen and colleagues [8] showed a statistically significant negative (i.e. in a causal interpretation: protective) influence of inspections on work-related injuries (OR = 0.65, 95% CI 0.73–0.93), non-compliance with occupational health and safety regulations (OR = 0.65, 95% CI 0.50–0.83) and on compensation claims (OR = 0.96, 95% CI 0.94–0.98). Dyreborg et al. (2022) examined the effectiveness of various interventions, including enforcement/compliance with legislation, on the risk of occupational accidents in relation to the length of observation period. They find high evidence for a small effect for the association between enforcement of laws and regulations and accident risk for a follow-up period of 36 months or longer (OR = 0.95, 95% CI 0.93–0.97).

Furthermore, on the basis of an “evidence and gap map”, Bondebjerg et al. [9, 23] concluded that there is evidence for the effectiveness of inspections. Van der Molen et al. [21, 22] did not find sufficient evidence that non-fatal accidents at work could be prevented by inspections based on results of one study. Data extraction tables of the systematic reviews are presented in the supplement.

### Epidemiological and intervention studies

We included 17 publications from Europe [25–41] including Norway (*n* = 5), Germany (*n* = 4), Italy (*n* = 2), Spain (*n* = 2), Austria (*n* = 1) Greece (*n* = 1), Switzerland (*n* = 1) and European countries (*n* = 1). Fourteen publications came from Northern America, i.e. the US (*n* = 9) and Canada (*n* = 5) [42–54]. Data extraction of single studies is presented in the supplementary material. To investigate the relationship between inspections and occupational safety, some studies focused on occupational groups with a high risk of injury, such as employees in nursing homes [30–32, 54], construction/ transport/ manufacturing [44, 45], general manufacturing [25, 26, 47, 50, 52], mining [35, 55], and the construction industry [48]. Other studies did not focus on a specific occupational group but may still have included high-risk establishments, as these are typically prioritised for inspections, e.g. “Site-Specific Targeting (SST) Program” conducted by the OSHA [27, 28, 36, 37, 39, 40, 42, 43, 49, 51, 53]. Most studies examined the effect of labour inspections on (fatal and non-fatal) injuries [25, 26, 36, 37, 48–51, 54]. Other health outcomes were physician certified sick leave due to musculoskeletal disorders (MSD) and psychological diagnoses [31] and absenteeism [39]. Four publications used compensation claims [42, 44–46]. There was no study investigating the effect of inspections on occupational diseases. Tatsaki et al. [40] investigated the effect of occupational injuries on sanctions. Nine publications analysed the effectiveness of inspections in implementing measures and complying with legal requirements [27–29, 32, 33, 35, 38, 41, 42]. The behaviour of companies with regard to repeated violations was examined in three studies [47, 52, 53]. In addition, one study investigated the influence of OSHA inspections on future OSHA inspections [53].

The results of the individual studies generally indicate an association between labour inspections and a reduction in work-related injuries [25, 26, 36, 37, 49–51, 54, 56]. In the included randomised controlled trial (RCT), there was a tendency towards a decrease in sick days and sick time due to MSD diagnoses and an increase in sick days and sick time due to psychological diagnoses during visits; however, the results were not statistically significant [31]. Based on a single item question, one study observed that inspections were associated with higher absenteeism rates [39]. There were heterogenous results for the effect of inspections on compensation claims [44, 45]: A reduction in the claims rate was only observed for transportation companies in the second year, where more than one inspection was carried out. For construction companies, on the other hand, there was an increase in claims in the first year, and there was no association for the manufacturing industry. There was no differentiation by cause of illness in the mentioned analysis. Another publication found an association between labour inspections and compensation claims for acute injuries only when outliers were excluded [46].

There were also improvements in the implementation of measures or legal requirements in the workplace as a result of labour inspections [27, 29, 32, 33, 35, 38, 43], with two studies showing improvements in both the intervention group and the control group, which consisted of companies that were not inspected during the study period [28, 41].

### Synthesis of results

A meta-analysis was performed for the effect of inspections on the risk of work-related injuries and compliance with workplace safety regulations. Therefore, suitable studies from the literature update and the systematic review by Andersen et al. [8] were included the meta-analysis.

### Injuries

We included 10 publications from Andersen et al. [8] in the meta-analysis [25, 57–65] and two studies from the literature update [37, 49]. A summary of all studies included in the meta-analysis is given in Table 1 which also includes the definition of outcomes and inspection type which differed slightly between studies.

**Fig. 2:**
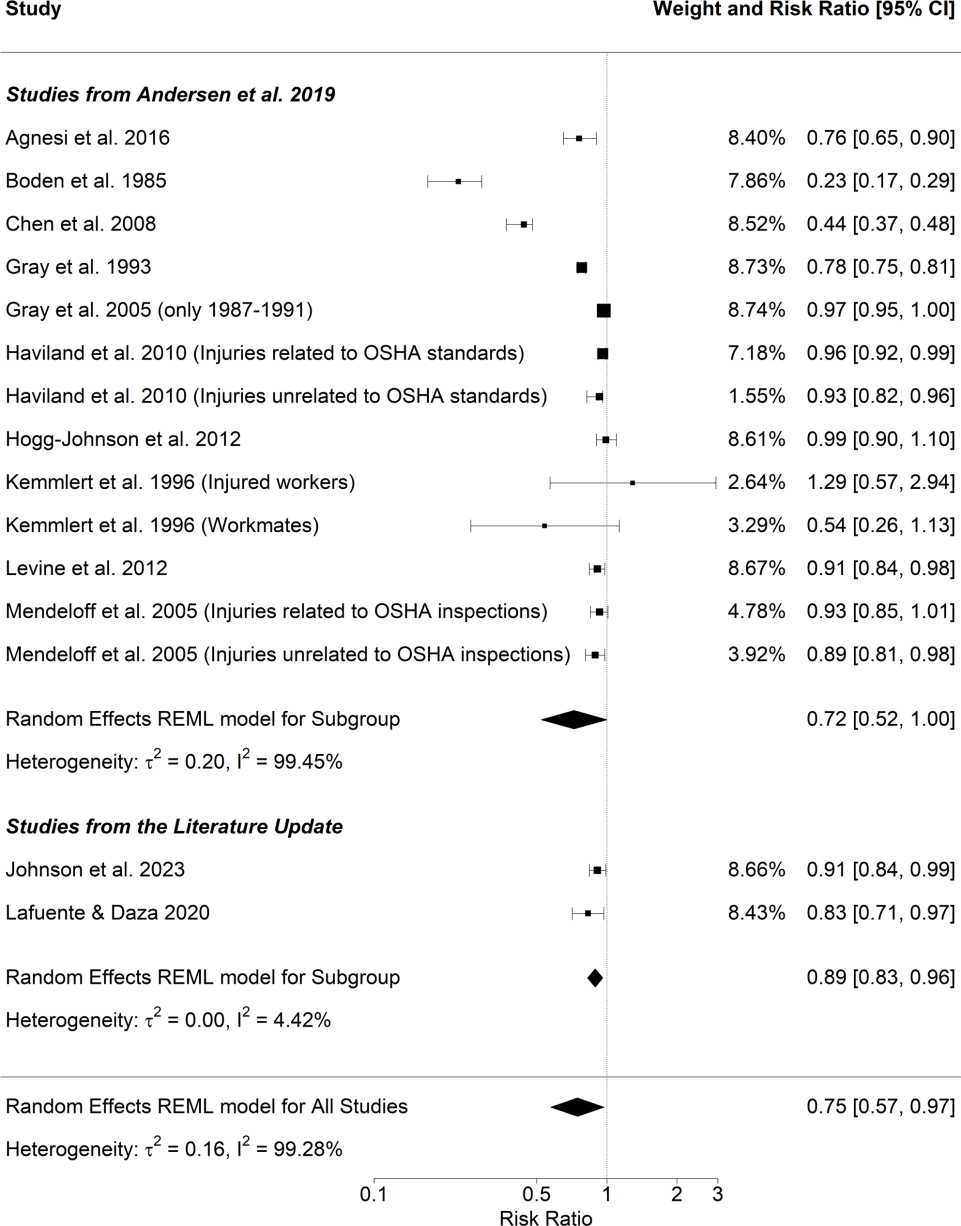
Meta-analysis for the association between inspections and injuries

The pooled risk estimate for the effect of inspections on the risk of injuries was 0.75 (95% CI 0.57–0.97; Fig. 2), indicating a 25% lower risk of injury in inspected workplaces compared with non-inspected workplaces. Heterogeneity was very high (I^2^ = 99.28%). Sub-group analyses, in which studies from Andersen et al. [1] and the literature update were investigated separately, also align with the pooled analysis results. The risk estimates were RR = 0.72 (95% CI 0.52–1.00) for the meta-analysis based only on the studies from Andersen et al. [1], and RR = 0.89 (95% CI 0.83–0.96) for the later published studies from the literature update.

**Table 1:** Studies included in the meta-analysis on the association between inspection and injuries

Review	First author and year of publication	Country	Period	Occupations considered	Outcome	Intervention
Andersen	Agnesi 2016	Italy	2001–2007	Manufacturing	Injuries with absence^1^	Workplace inspection^1^ after accident^1^
Andersen	Boden 1985	USA	1973–1975	Coal mines	Disabling accidents^1^	Mine safety inspection^1^
Andersen	Chen 2008	USA	1996–2003	Truck companies	Truck crash injuries^1^	Monitoring of a motor carrier safety review^1^
Andersen	Gray 1993	USA	1979–1985	Manufacturing	Injuries^1^	Penalty inspection^1^
Andersen	Gray 2005	USA	1987–1991	Manufacturing	Lost workday injuries^1^	Penalty inspection^1^
Andersen	Haviland 2010	Pennsylvania, USA	1998–2005	No restriction	Injuries related to OSHA standards^1^	Penalty inspection^1^
					Injuries unrelated to OSHA standards^1^	Penalty inspection^1^
Andersen	Hogg Johnson 2012	Canada	2002–2008	No restriction	Injuries^1^	Inspection^1^
Andersen	Kemmlert 1996	Sweden	1985	No restriction	Injured workers^1^	Inspection^1^
					Workmates^1^	Inspection^1^
Andersen	Levine 2012	California, USA	1996–2006	No restriction	Injury count^1^	Inspections^1^
Andersen	Mendeloff 2005	USA	1990–1998	No restriction	Injuries related to OSHA standards^1^	Inspection^1^
					Injuries unrelated to OSHA standards^1^	Inspection^1^
Update	Johnson 2023	USA	2001–2010	No restriction	Injuries per 100 FTE	Planned inspection
Update	Lafuente 2020	Europe	2008–2015	No restriction	Injuries	Inspection per employee

^1^ Definition according to Fig. 2 in Andersen et al. [8]

### Compliance

To investigate the effect of labour inspections on compliance four studies [66–69] from Andersen et al. [8] and one study [53] from the literature update were available for meta-analysis. A description of studies included is given in Table 2.

**Fig. 3:**
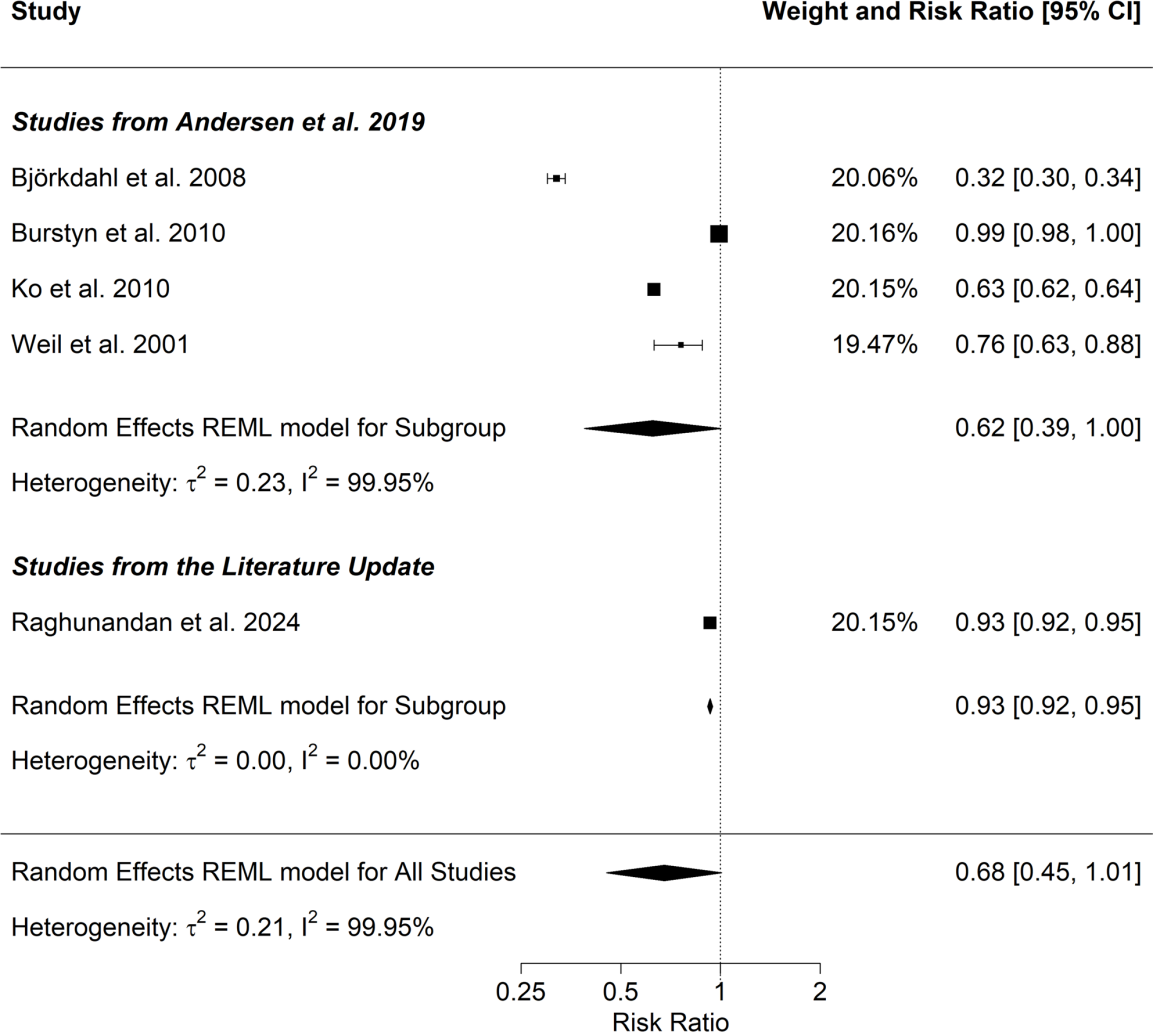
Meta-analysis on the effect of inspections on the risk of non-compliance

Pooled estimates show that labour inspections reduce the likelihood of non-compliance (RR = 0.68, 95% CI 0.45–1.01; Fig. 3); the results almost approached statistical significance. Heterogeneity was very high (I^2^ = 99.95%). Similar results were obtained in the subgroup analysis which only contained the studies from Andersen et al. [8] (RR = 0.62, 95% CI 0.39–1.00).

**Table 2:** Studies included in the meta-analysis on the association between inspection and compliance

Review	First author and year of publication	Country	Period	Occupations considered	Outcome	Intervention
Andersen	Björkdahl 2008	Sweden	2005	Industry sector	Noise awareness and compliance^1^	Inspections in a noise regulation campaign^1^
Andersen	Burstyn 2010	Canada	2003–2006	Industry sector	Compliance^1^	Supportive inspection^1^
Andersen	Ko 2010	USA	1972–2006	Manufacturing	Compliance, violations, all^1^	Inspection^1^
Andersen	Weil 2001	USA	1987–1993	Construction	Compliance, all^1^	Inspection^1^
Update	Raghundan 2024	USA	2002–2016	No restriction	Violation	Inspection

^1^ Definition according to Fig. 2 in Andersen et al. [8]

## Discussion

In summary, this scoping review included six systematic reviews, one RCT, and 30 observational studies published between 2017 and 2024, of which three were included in the pooled analysis together with the studies identified by Andersen et al. [8]. Studies often investigated the association between inspections and injuries and compliance in high-risk occupations. Overall, the scoping review with meta-analysis shows that labour inspections were associated with a reduced risk of work-related injuries and non-compliance in inspected workplaces compared with non-inspected workplaces, indicating a protective effect of inspections on occupational safety and health. Our results reinforce the findings of previous (systematic) reviews that inspections are effective in reducing work-related injuries and increase compliance [9–12].

The results for the association between labour inspections and compensation claims were ambiguous: In two study that examined the effect of labour inspections on compensation claims one and two years later, the outcomes, injuries and musculoskeletal disorders, were not analysed separately [44, 45]. MSD often have a long latency period between the onset of occupational exposure and diagnosis. Therefore, it is unlikely that workplace inspections will reduce compensation claims due to MSD in the first or second year after the inspection. This is supported by observations from the RCT study: This study observed only a tendency for a decrease in sick leave days and periods for musculoskeletal diagnoses certified by a physician after 18 months in the intervention group [23]. The small, not statistically significant effects observed may be due to the short duration of the study. Therefore, longer study periods are necessary to estimate the effect of inspection on occupational diseases, such as MSD.

The limitation of short study periods, often encountered in primary studies, can be overcome by using official databases. The majority of studies included in this review used official data from sources such as the OSHA, which has provided comprehensive data since 1972. In Germany, however, the necessary data is unavailable and violations are not made public. Accident insurance providers not yet provided anonymous data. Consequently, it is impossible to investigate the impact of inspections on work-related injuries and compliance using official data in Germany. As most of the included studies came from the USA, the results on the effect of labour inspections cannot readily be transferred to Germany, as it has a different legal basis. Therefore, access to occupational health and safety data, ideally as continuous monitoring with open access to anonymised data, is essential for future research into occupational health and safety in Germany as well as in other countries.

Although, according to our results, labour inspections are effective in the protection of occupational safety and health, the results of the scoping review also suggest that various factors at the company level (experience, duration, size, sector), at the enforcement level (scope, level of sanctions, consultation insurance, exchange of information and personal characteristics of the inspectors) and at the governmental level can modify the effectiveness of labour inspections:

Effectiveness of inspections might be influenced at the company level: Repeated violations of occupational safety regulations have been shown to become more frequent after inspections in smaller companies with up to 20 employees, but less frequent in large companies with 250 or more employees [52]. Small businesses with fewer than 10 employees and medium-sized businesses with 11 to 100 employees are more likely to have occupational safety deficiencies than larger businesses, which tend to employ occupational safety specialists and company doctors. Larger companies are inspected more frequently, which may be reflected in the number of violations and orders issued, despite often having established structures in place to ensure occupational safety. In addition, the selection of inspected industries or companies can influence the effect of inspections. One approach is to prioritise key industries and companies with comparatively high accident rates, but not exclusively, as this could negatively impact occupational safety in establishments just below the threshold for high-risk establishments. For example, this is done in the “Site-Specific Targeting (SST) Program” conducted by the OSHA in the US, where inspection list selection criteria are provided for high-rate establishments, upward trending establishments, low rate establishments and non-responders [70].

At the enforcement level, studies have shown that the effectiveness of enforcing safety regulations increases with high penalties, but not low ones [39, 44, 61]. Furthermore, labour inspections involving orders have been found to be more effective in implementing risk management measures and reducing work-related injuries than inspections without orders [35, 38]. Andersen et al. [3] also found that inspections involving orders or higher penalties were associated with fewer violations and better compliance with regulations. However, the results regarding the impact of orders and penalties cannot be generalised; orders or fines may only be necessary in companies that do not respond to the audit letter following an inspection. Nevertheless, the results suggest that the expected consequences of violating occupational safety regulations can influence future compliance with these regulations. Furthermore, the results of a US study show that a combination of inspections and consultation visits might lead to a larger reduction in fatalities than inspections alone [40]. Here, it was inspections (and consultations) rather than higher average penalties that were associated with lower fatality rates. Two further studies found that workplace inspections followed by an additional consultation session for managers were more effective at prompting the implementation of safety measures [21, 35]. Therefore, labour inspections involving consultations, together with the prospect of severe penalties, could improve occupational safety and health.

Personal factors of the inspectors may also influence inspection effects. One study showed that the effectiveness of occupational safety and health could be increased by personal factors and the ability of supervisors or inspectors to explain the changes necessary to comply with regulations [35]. In addition, inspectors may pay more attention to existing violations and to standards with high risk potential and therefore report them more frequently [47, 52]. Thus, the educational background and qualifications of inspectors may affect the implementation and thematic focus of inspections [71]. In addition, to date there is no standardized way on how to measure OSH [72] leading to difference in the implementation and methods of inspections. Thus, labour inspectors should be provided with (standardized) strategies for monitoring and enforcing OSH legislation. This would also increase comparability of inspection effects within and between countries.

At the governmental level the following has been observed: A study from the US shows that states that exempt small firms from the requirement to purchase workers’ compensation insurance, and states with longer waiting periods for workers compensation, have higher fatality rates [48]. Additionally, companies that had committed violations in one US state subsequently committed fewer violations in that state, but more in others [53]. The authors describe this as ‘information friction’. One study based on OSHA data showed that labour inspections at one company location do not necessarily lead to improvements in occupational safety at another company location [50]. Therefore, labour inspections conducted at a regional level could result in a company with multiple locations shifting its OSH priorities. This may favour more comprehensive inspections on the national level. Also, sharing information within and between authorities helps to increase compliance. Additionally, the results of a US study indicate that there were fewer re-violations under democratic governments, which is potentially due to stricter OSHA enforcement practices [52]. This observation is supported by current developments in the US under the Republican administration. Legislation has been reintroduced to abolish OSHA [73]. Under the current US government, a number of OSHA and Mine Safety and Health Administration (MSHA) offices have been closed down, almost all NIOSH employees have been dismissed, and OSHA programs such as the firefighter health program have been cut. This is intended to reduce the spending. Yet, the social and economic costs of work-related injuries and illnesses are already high, estimated at between $174 billion and $348 billion per year. Thus, the number of work-related injuries and cases of non-compliance might increase in the near future resulting in even greater social and economic costs.

A reduction in inspectorate staff is being observed worldwide. Against the background of the positive impact of labour inspections by the state labour inspectorate, it is critical to view the fact that the number of inspectors in the state labour inspectorates in Germany fell from 3,600 in 1991 to 1,620 in 2023. The number of state occupational physicians also fell during this period from 126 in 1991 to 47 in 2023. During the same period, the number of company inspections of the labour inspectorates fell from 624,014 in 1991 to 133,564 in 2023. The number of inspectors from the accident insurance institutions also fell, albeit less significantly, from 2,543 in 1991 to 2,160 in 2023. The number of inspections of companies carried out by the supervisors of the accident insurance institutions fell from 543,157 in 1991 to 502,859 in 2023 [7, 74]. A similar trend is also being observed in other countries. According to the ILO, the number of inspectors has decreased between 2009 and 2022 (or the closest available year) in 43% of 75 countries while employment increased [75]. The data also shows that, of the 75 countries for which data was available, 87% conducted fewer visits than liable to inspection, and in 71% of countries it was less than half of the workplaces liable to inspection. Moreover, inspectors are responsible for larger areas leading to work intensification [76]. In addition to their occupational safety duties, German inspectors have become increasingly responsible for other areas, such as in emission control and product safety. It is estimated that 40% of supervisory capacity is devoted to activities in legal areas outside of occupational safety [77]. Around the world, the worsening of working conditions for inspectors has been recognised, which may lead to inspections being carried out by less skilled personnel with insufficient equipment and material resources [75]. Therefore, working as an inspector may involve various occupational hazards. A recent study showed that inspectors are exposed to ergonomic, psychosocial, organisational, physical, chemical and biological risk factors at work. Their work is characterised by high job demands, time pressure, and exposure to harassment, increasing the risk of psychological disorders such as burnout [78–80]. Consequently, the deteriorating working conditions of inspectors may also reduce the effectiveness of inspections.

### Strenghts and limitations

This study has the following limitations: The literature update did not include an assessment of risk of bias. This was because the review was performed as a scoping review which usually does not include a quality assessment [81, 82]. Although, there was no overall risk assessment, the primary studies tended to be of rather low risk, because for instance of outcome definition [39, 44, 45], missing data on results [28], and low response rates [27, 39]. Additionally, some studies only interviewed the person who knew the most about OSH, which may have biased the observed results [39, 41]. For both meta-analyses, there was high heterogeneity between the studies, with an I² value approaching 100%. This may be due to differences in the definition of outcomes, settings, regulations and the content of the intervention between the studies. This is a problem that was already recognised by Andersen and colleagues [3]. The comparability of the results may also be limited due to wide variation in occupational safety systems and regulations around the world. Thus, the pooled estimated must be interpreted with caution. Furthermore, we also did not conduct a GRADE (Grading of Recommendations, Assessment, Development and Evaluation) assessment of the quality of evidence and strength of recommendations for the results of the meta-analyses. Andersen et al. [8] observed a moderate evidence for the effect of inspections on injuries and compliance, and low evidence for compensation claims. For future research more homogenised data would be desirable to investigate the effect of labour inspections on occupational safety and health, for instance by considering the ILO guide on harmonisation of labour inspection statistics [83]. Additionally, publication bias was not assessed, in line with common practice for scoping reviews [14, 82]. In their systematic review, Andersen and colleagues did not observe a major risk of publication bias for work-related injuries but a large heterogeneity for compliance [8]. The risk of publication bias on the effect of inspections on OSH has to be addressed in future research. Moreover, this study has the substantial limitation that it included only one RCT and predominantly observational studies. RCTs are considered the “gold standard” for generating reliable evidence [84]. Therefore, the results obtained from the pooled analysis of mainly observational studies need to be verified by RCTs.

Although this scoping review provides evidence that labour inspections reduce work-related injuries and non-compliance, the transferability of these findings to other countries is constrained by differences in inspection regimes, enforcement practices, and labour market structures. Most included studies originate from the United States, where inspections operate within a federal regulatory framework. Moreover, the availability and application of enforcement instruments, as well as the balance between different inspection approaches, vary across countries. Differences in inspector training, resources, and the use of consultative measures may further influence inspection outcomes. Consequently, cross-country transfer of evidence requires careful consideration of institutional and structural contexts, as well as improved access to harmonised and longitudinal inspection data, which should be included in future research.

Furthermore, the included studies were conducted prior to the COVID-19 pandemic. Therefore, the observed association between inspections and occupational safety and health needs to be verified in the context of post-pandemic changes to the work environment. The pandemic has accelerated remote work, flexible schedules, and decentralised work arrangements [85], which may reduce the visibility of occupational hazards and complicate traditional inspection approaches. In addition, post-pandemic resource constraints faced by both companies and regulatory authorities may limit the frequency and intensity of inspections [86, 87]. Consequently, the magnitude and mechanisms of the protective effect of labour inspections could differ in the post-pandemic context, underscoring the need for adaptive inspection strategies, innovative monitoring tools, and updated research that reflects new work environments and organisational practices. The strengths of this scoping review include a sensitive literature search and an extensive grey literature search. Furthermore, the studies included in this review were extracted into detailed data extraction tables, providing a structured overview of all relevant aspects, including general information, population, exposure, outcomes and results. In addition, the literature searches were quantitatively synthesised through meta-analysis together with studies from the systematic review by Andersen et al. [8]. Thus, this study provides information on the effect of labour inspections on OSH based on almost 60 years of research.

## Conclusion

This study strengthens the empirical evidence that workplace inspections might have a protective effect on occupational safety and health, as inspected workplaces exhibit a lower risk of injuries and non-compliance. These findings underscore the central role of inspections in occupational health and safety and highlight their importance for policymakers. To increase their effectiveness, policy should adopt a comprehensive approach that includes targeting high-risk industries, strengthening coordination and communication within and between well-resourced authorities, and fostering sustained engagement with companies. In addition, the strategic use of consultations, enforcement orders, and substantial penalties can further enhance compliance and reinforce the overall impact of OSH policy.

## Supplementary Information

Below is the link to the electronic supplementary material.

Supplementary Material 1

## Data Availability

No datasets were generated or analysed during the current study.

## Abbreviations

AFL: CIO–American Federation of Labour–Congress of Industrial Organisation
BAuA: Bundesanstalt für Arbeitsschutz und Arbeitsmedizin (Federal Institute for Occupational Safety and Health)
CI: Confidence Interval
COVID: Coronavirus Disease
EFTA: European Free Trade Association
GRADE: Grading of Recommendations, Assessment, Development and Evaluation
ILO: International Labour Organisation
MSD: Musculoskeletal Disorders
NIOSH: National Institute for Occupational Safety and Health
OR: Odds Ratio
OSH: Occupational Safety and Health
OSHA: Occupational Safety and Health Administration
PICO: Population–Intervention–Comparison/Context–Outcome
RCT: Randomized Controlled Trial
REML: Restricted Maximum Likelihood
RR: Relative risk
SST: Site-Specific Targeting (SST)
USA: United States of America
