# Correction: Scoping review on the effect of labour inspections on occupational health and safety: a meta-analytic update

**DOI:** 10.1186/s12995-026-00502-y

**Published:** 2026-03-06

**Authors:** Melanie Schubert, Ulrich Bolm-Audorff, Johan Hviid Andersen, Gabriela Petereit-Haack, David Reissig, Andreas Seidler

**Affiliations:** 1 Institute and Policlinic of Occupational and Social Medicine, Faculty of Medicine, Technische Universität Dresden, Dresden, Germany; 2 Department of Occupational Medicine, Danish Ramazzini Centre, Regional Hospital West Jutland – University Clinic, Herning, Denmark; 3 Division of Occupational Health, Department of Occupational Safety, Regional Government of South Hesse, Wiesbaden, Germany

**Correction: J Occup Med Toxicol 21, 5 (2026)**

10.1186/s12995-026-00497-6

In the Discussion section of this article, the ninth paragraph on page 10 contains errors in the reported numbers. The correct and incorrect version is presented below, and the original article has been updated.

**Incorrect version**

Section: **Discussion**

A reduction in inspectorate staff is being observed worldwide. Against the background of the positive impact of labour inspections by the state labour inspectorate, it is critical to view the fact that the number of inspectors in the state labour inspectorates in Germany fell from 3,600 in 1991 to 2,257 in 2023. The number of state occupational physicians also fell during this period from 126 in 1991 to 20 in 2023.

**Correct version**

A reduction in inspectorate staff is being observed worldwide. Against the background of the positive impact of labour inspections by the state labour inspectorate, it is critical to view the fact that the number of inspectors in the state labour inspectorates in Germany fell from 3,600 in 1991 to **1,620** in 2023. The number of state occupational physicians also fell during this period from 126 in 1991 to **47** in 2023.

