# Supplementary material for: Scoping review on the effect of labour inspections on occupational health and safety: a meta-analytic update
Source: J Occup Med Toxicol. 2026 Feb 12;21:5. doi: 10.1186/s12995-026-00497-6 (PMC12903292; doi:10.1186/s12995-026-00497-6)
Supplement: Supplementary file 1 — Supplementary Material 1 [file 12995_2026_497_MOESM1_ESM.pdf]

1 Key studies

Key study	Found in Pubmed	Found in WoS<br>Original search string Andersen etl al.<br>*Adjusted search string*
1. Andersen JH, Malmros P, Ebbehoej NE, Flachs EM, Bengtsen E, Bonde JP. Systematic literature review on the effects of occupational safety and health (OSH) interventions at the workplace. Scand J Work Environ Health 2019;45(2):103-13.	yes	yes<br>*yes*
2. Bondebjerg A, Filges T, Pejtersen JH, Kildemoes MW, Burr H, Hasle P, et al. Occupational health and safety regulatory interventions to improve the work environment: An evidence and gap map of effectiveness studies. Campbell Syst Rev 2023;19(4):e1371.	yes	no (in WoS)<br>*yes*
3. Tompa E, Kalcevich C, Foley M, McLeod C, Hogg-Johnson S, Cullen K, et al. A systematic literature review of the effectiveness of occupational health and safety regulatory enforcement. Am J Ind Med 2016;59(11):919-33.	No (in Pubmed database)<br>-> search string adjusted with keywords used in publication<br>-> yes	no (in WoS)<br>*yes*
4. van der Molen HF, Basnet P, Hoonakker PL, Lehtola MM, Lappalainen J, Frings-Dresen MH, et al. Interventions to prevent injuries in construction workers. Cochrane Database Syst Rev 2018;2(2):Cd006251.	yes	no (in WoS)<br>*yes*
5. Dyreborg, J., Lipscomb, H. J., Nielsen, K., Törner, M., Rasmussen, K., Frydendall, K. B., Bay, H., Gensby, U., Bengtsen, E., Guldenmund, F., & Kines, P. (2022). Safety interventions for the prevention of accidents at work: A systematic review. Campbell Systematic Reviews, 18(e1234), 1–187. https://doi.org/10.1002/cl2.1234	yes	no (in WoS)<br>*yes*
6. Li, L., & Singleton, P. (2019). The effect of workplace inspections on worker safety. ILR Review, 72(3), 718–748. https://doi.org/10.1177/0019793918801575	No (not in Medline database)	no (in WoS)<br>*yes*
7. Agnesi R, Fedeli U, Bena A, Farina E, Sarto F, Veronese M, et al. Statutory prevention of work injuries in Italy: an effectiveness evaluation with interrupted time series analysis in a sample of 5000 manufacturing plants from the Veneto region. Occup Environ Med 2016;73(5):336-41.	yes	no (in WoS)<br>*yes*
8. Farina E, Bena A, Fedeli U, Mastrangelo G, Veronese M, Agnesi R. Public injury prevention system in the Italian manufacturing sector: What	yes	no (in WoS)<br>*yes*

	types of inspection are more effective? Am J Ind Med 2016;59(4):315-21.		
9.	Dahl, Ø., Rundmo, T., & Olsen, E. (2022). The impact of business leaders'formal health and safety training on the establishment of robust occupational safety and health management systems: Three studies based on data from labour inspections. International Journal of Environmental Research and Public Health, 19(1269), 1–14. https://doi.org/10.3390/ijerph19031269	no (in Pubmed database)<br>-> search string adjusted with keywords used in publication -> yes	no (in WoS)<br>yes

2 Search string (modified from Andersen et al. 2019)

(Andersen JH, Malmros P, Ebbehoej NE, Flachs EM, Bengtsen E, Bonde JP. Systematic literature review on the effects of occupational safety and health (OSH) interventions at the workplace. Scand J Work Environ Health. 2019 Mar 1;45(2):103-113. doi: 10.5271/sjweh.3775. Epub 2018 Oct 29. PMID: 30370910.)

2.1 PubMed

Topic	keywords
#1<br>Regulatory focus	"accidents, occupational"[MeSH Terms] OR "occupational accidents"[Title/Abstract] OR "occupational diseases"[MeSH Terms] OR "occupational exposure"[MeSH Terms] OR "occupational diseases"[Title/Abstract] OR "occupational health"[MeSH Terms] OR "health and safety"[Title/Abstract] OR "safety and health"[Title/Abstract] OR "occupational safety"[Title/Abstract] OR OHS[Title/Abstract] OR OSH[Title/Abstract] OR "working environment"[Title/Abstract] OR "occupational injury"[Title/Abstract] OR "occupational injuries"[Title/Abstract] OR "law enforcement"[MeSH Terms] OR inspector*[Title/Abstract] OR "government regulation"[MeSH Terms] OR "OSH systems"[Title/Abstract]
#2<br>Setting	workplace[MeSH Terms] OR work[MeSH Terms] OR worker*[Title/Abstract] OR work-site[Title/Abstract] OR worksite*[Title/Abstract] OR enterprise[Title/Abstract] OR employee*[Title/Abstract] OR self-employed[Title/Abstract] OR violation[Title/Abstract] OR breaches[Title/Abstract] OR offence*[Title/Abstract] OR compliance[Title/Abstract]
#3<br>Regulatory focus AND setting	#1 AND #2
#4<br>Policy levers	fined[Title] OR prosecution[Title/Abstract] OR inspection*[Title/Abstract] OR inspectorate*[Title/Abstract] OR "improvement notices"[Title/Abstract] OR "mandatory consultancy"[Title/Abstract] OR "deviation report"[Title/Abstract] OR "policy levers"[Title/Abstract] OR "regulatory levers"[Title/Abstract] OR "ohs services"[Title/Abstract] OR smiley*[Title/Abstract] OR "practice guidelines as topic"[MeSH Terms] OR ((guideline*[Title/Abstract]) AND safety[Title/Abstract]) OR "osh management system"[Title/Abstract] OR "osh management systems"[Title/Abstract] OR "safety management system"[Title/Abstract] OR "safety management systems"[Title/Abstract] OR "OHSAS 18001" OR "OHSMS BS 8800" OR (audit*[Title/Abstract] AND (safety[Title/Abstract] OR health[Title/Abstract])) OR accreditation[MeSH Terms] OR enforcement[Title/Abstract] OR "health and safety training"[Title/Abstract] OR (order*[Title/Abstract] AND (safety[Title/Abstract] OR health[Title/Abstract])) OR (sanction*[Title/Abstract] AND (safety[Title/Abstract] OR health[Title/Abstract])) OR (incentive*[Title/Abstract] AND (safety[Title/Abstract] OR health[Title/Abstract])) OR ((financ*[Title/Abstract] OR econom*[Title/Abstract]) AND (safety[Title/Abstract] OR health[Title/Abstract])) OR "regulatory effectiveness"[Title/Abstract] OR regulation[Title/Abstract] OR "management systems"[Title/Abstract]
#5<br>Regulatory focus AND	#3 AND #4

setting AND Policy levers	
#6 context (conditions & outcomes)	"statistics and numerical data"[MeSH Subheading] OR "controlled before after studies"[MeSH Terms] OR "before and after study"[Title/Abstract] OR "before and after studies"[Title/Abstract] OR (accident*[Title/Abstract] AND report*[Title/Abstract]) OR (injuries[Title/Abstract] AND report*[Title/Abstract]) OR "key performance indicators"[Title/Abstract] OR “OSH monitoring system”[Title/Abstract] OR “OSH surveillance system”[Title/Abstract] OR “insurance claim”[Title/Abstract] OR "accident prevention"[MeSH Terms] OR "accidents, occupational"[MeSH Terms] OR "occupational injuries"[MeSH Terms] OR "occupational diseases"[MeSH Terms] OR "occupational disorder"[Title/Abstract] OR “occupational health and safety”[Title/Abstract] OR “safety and health”[Title/Abstract] OR absence [Title/Abstract] OR “sick leave” [MeSH Terms]
#7 Regulatory focus AND setting AND policy levers AND context (conditions & outcomes)	#5 AND #6

2.2 Web of Science Core Collection

Topic	keywords
#1	TS=(“occupational accident” OR “occupational disease” OR “occupational exposure” OR “occupational health” OR “occupational injury” OR “occupational injuries” OR “health and safety” OR “occupational health and safety” OR “accident prevention” OR “work injury” OR “work injuries” OR “safety and health” OR “injury prevention”)
#2	TS=(OSH OR law enforcement OR inspect* OR prosecution OR “mandatory consultancy” OR “deviation reports” OR "policy levers" OR "regulatory levers" OR "OHS services" OR “osh management system*” OR “safety management system” OR "OHSAS 18001" OR “OHSMS BS 8800" OR “regulatory effectiveness” OR “management system” OR “OSH monitoring system” OR “surveillance system” OR “insurance claim”<br>OR “regulatory intervention” OR “effectiveness study” OR “effectiveness studies” OR “regulatory effectiveness” OR “safety intervention” OR “work inspection” OR “statutory prevention” OR “OSH system” OR “effectiveness evaluation”)
#3	#1 AND #2

2.3 NIOSHTIC-2

(AB like '%occupational accident%' or kw like '%occupational accident%' or ab like '%occupational exposure%' or kw like '%occupational exposure%' or ab like '%occupational health%' or kw like '%occupational health%' or ab like '%occupational injur%' or kw like '%occupational injur%' or ab like '%occupational safety%' or kw like '%occupational safety%' or ab like '% osh %' or kw like '% osh %') and (ab like '%workplace%' or kw like '%workplace%' or ab like '%work site%' or kw like '%work site%' or ab like '%worksite%' or kw like '%worksite%' or ab like '%worker%' or kw like '%worker%' or ab like '%enterprise%' or kw like '%enterprise%' or ab like '%employe%' or kw like '%employe%' or ab like '%self-employed%' or kw like '%self-employed%' or ab like '%violation%' or kw like '%violation%' or ab like '%breaching%' or kw like '%breaching%' or ab like '%trades%' or kw like '%trades%')
AND
(ab like '%fine%' or kw like '%fine%' or ab like '%prosecution%' or kw like '%prosecution%' or ab like '%bans%' or kw like '%bans%' or ab like '%statistic%' or kw like '%statistic%' or ab like '%regulatory lever%' or kw like '%regulatory lever%' or ab like '%policy lever%' or kw like '%policy lever%' or ab like '%OHS service%' or kw like '%OHS service%' or ab like '%guidance%' or kw like '%guidance%' or ab like '%guideline%' or kw like '%guideline%' or ab like '%inspection%' or kw like '%inspection%' or ab like

'%inspectorate%' or kw like '%inspectorate%' or ab like '%OHSAS 18001%' or kw like '%OHSAS 18001%' or ab like '%OHSMS BS 8800%' or kw like '%OHSMS BS 8800%' or ab like '%audit%' or kw like '%audit%' or ab like '%accreditation%' or kw like '%accreditation%' or (ab like '%enforcement%' or kw like '%enforcement%' AND (ab like '%law%' or kw like '%law%')) or ab like '%safety training%' or kw like '%safety training%' or ab like '%investigations%' or kw like '%investigations%' or ab like '%sanction%' or kw like '%sanction%' or ab like '%economic incentive%' or kw like '%economic incentive%' or ab like '%financial incentive%' or kw like '%financial incentive%')
AND
(AB like '%statistic%' or KW like '%statistic%' or AB like '%before-after%' or KW like '%before-after%' or (AB like '%accident%' or KW like '%accident%' AND (AB like '%report%' or KW like '%report%')) or AB like '%key performance indicator%' or KW like '%key performance indicator%' or AB like '%insurance claim%' or KW like '%insurance claim%' or AB like '%prevent%' or KW like '%prevent%' or AB like '%outcome%' or KW like '%outcome%' or AB like '%outcome%' or KW like '%outcome%' or AB like '%improved working conditions%' or KW like '%improved working conditions%' or AB like '%occupational accident%' or KW like '%occupational accident%' or AB like '%occupational injur%' or KW like '%occupational injur%' or AB like '%occupational fatalit%' or KW like '%occupational fatalit%' or AB like '%occupational disease%' or KW like '%occupational disease%' or AB like '%occupational disorder%' or KW like '%occupational disorder%' or AB like '%frequency%' or KW like '%frequency%' or AB like '%incidence rate%' or KW like '%incidence rate%' or AB like '%absence%' or KW like '%absence%' or AB like '%impact%' or KW like '%impact%' or AB like '%costs%' or KW like '%costs%' or AB like '%hazards%' or KW like '%hazards%' or (AB like '%workplace%' or KW like '%workplace%' AND (AB like '%health and safety%' or KW like '%health and safety%')) or AB like '%return to work%' or KW like '%return to work%' or ab like '%conclusion%')

*Searches #27 and #32 from Andersen et al (2019) disregarded; these return 0 results in NIOSHTIC-2.

3 Publications that could not be obtained during the literature search

3.1 Electronic database search

1. Canciani, D., & Canciani, D. (2019). *The Politics and Practice of Occupational Health and Safety Law Enforcement*. Basel, Switzerland: Springer International Publishing. https://doi.org/10.1007/978-3-319-98509-1 (Ausschlussgrund: Buch)
2. Oanh, T. T. D. (2021). Sanctions against administrative violations of occupational and health in enterprises. Journal of Law and Political Sciences; 32: 86-104. ISSN: 2518-5551 J9 - J LAW POLIT SCI (Ausschlussgrund: Medium in keiner Bibliothek vorhanden; nicht in Online-Ausgabe gefunden.)
3. Rosenman, K. D. (2024) Expanded program in occupational injury and illness surveillance. https://reporter.nih.gov/search/UAFstRwo_0ugkz_NbpYHGw/project-details/10318414 (Ausschlussgrund: Projektwebsite)

3.2 Other methods

1. Abildgaard, J. S., Rexen, T. S., & Sørensen, J. (2021). 'Dialogue and guidance'—A policy intervention for improved OHS among workplaces with WEA violations. ClinicalTrials.gov. https://clinicaltrials.gov/study/NCT05039398 (nur Verweis zur Registrierung; Autoren angeschrieben, aber keine Rückmeldung erhalten)

4 Included studies

1. Agnesi R, Fedeli U, Bena A, Farina E, Sarto F, Veronese M, Mastrangelo G. Statutory prevention of work injuries in Italy: an effectiveness evaluation with interrupted time series analysis in a sample of 5000 manufacturing plants from the Veneto region. Occup Environ Med. 2016 May;73(5):336-41. doi: 10.1136/oemed-2015-103227. Epub 2016 Mar 17. PMID: 26989139.
2. Andersen JH, Malmros P, Ebbehoej NE, Flachs EM, Bengtsen E, Bonde JP. Systematic literature review on the effects of occupational safety and health (OSH) interventions at the workplace. Scand J Work Environ Health. 2019 Mar 1;45(2):103-113. doi: 10.5271/sjweh.3775. Epub 2018 Oct 29. PMID: 30370910.
3. Beck D, Lenhardt U. Consideration of psychosocial factors in workplace risk assessments: findings from a company survey in Germany. Int Arch Occup Environ Health. 2019 Apr;92(3):435-451. doi: 10.1007/s00420-019-01416-5. Epub 2019 Feb 13. PMID: 30756179; PMCID: PMC6420464.
4. Bondebjerg A, Filges T, Pejtersen JH, Kildemoes MW, Burr H, Hasle P, Tompa E, Bengtsen E. Occupational health and safety regulatory interventions to improve the work environment: An evidence and gap map of effectiveness studies. Campbell Syst Rev. 2023 Dec 11;19(4):e1371. doi: 10.1002/cl2.1371. PMID: 38089568; PMCID: PMC10712440.
5. Bondebjerg A, Filges T, Pejtersen JH, Viinholt BCA, Burr H, Hasle P, Tompa E, Birkefoss K, Bengtsen E. PROTOCOL: Occupational health and safety regulatory interventions to improve the work environment: An evidence and gap map of effectiveness studies. Campbell Syst Rev. 2022 Apr 12;18(2):e1231. doi: 10.1002/cl2.1231. PMID: 36911348; PMCID: PMC9005925.
6. Brückner B, Hellbach R, Sommer S, Stamm R, Engelberg M, Hammer A, Koall J, Winecke M, Rissler J. GDA-Wirkungsprojekt Ergebnisbericht. 2018. Geschäftsstelle der Nationalen Arbeitsschutzkonferenz, Bundesanstalt für Arbeitsschutz und Arbeitsmedizin. Berlin. Deutschland.
7. Dahl Ø, Rundmo T, Olsen E. The Impact of Business Leaders' Formal Health and Safety Training on the Establishment of Robust Occupational Safety and Health Management Systems: Three Studies Based on Data from Labour Inspections. Int J Environ Res Public Health. 2022 Jan 24;19(3):1269. doi: 10.3390/ijerph19031269. PMID: 35162292; PMCID: PMC8834684.
8. Dyreborg J, Lipscomb HJ, Nielsen K, Törner M, Rasmussen K, Frydendall KB, Bay H, Gensby U, Bengtsen E, Guldenmund F, Kines P. Safety interventions for the prevention of accidents at work: A systematic review. Campbell Syst Rev. 2022 Jun 1;18(2):e1234. doi: 10.1002/cl2.1234. PMID: 36911341; PMCID: PMC9159701.
9. Fan, D., Yeung, A. C., Yiu, D. W., & Lo, C. K. (2022). Safety regulation enforcement and production safety: The role of penalties and voluntary safety management systems. *International Journal of Production Economics*, *248*, 108481. https://doi.org/10.1016/j.ijpe.2022.108481
10. Farina E, Bena A, Fedeli U, Mastrangelo G, Veronese M, Agnesi R. Public injury prevention system in the Italian manufacturing sector: What types of inspection are more effective? Am J Ind Med. 2016 Apr;59(4):315-21. doi: 10.1002/ajim.22576. Epub 2016 Feb 22. PMID: 26901855.
11. Finnanger Garshol B, Knardahl S, Emberland JS, Skare Ø, Johannessen HA. Effects of the Labour Inspectorate Authority's regulatory tools on psychosocial and biomechanical work factors in Norwegian home care services: a cluster randomised controlled trial. Occup Environ Med. 2022 Dec;79(12):807-815. doi: 10.1136/oemed-2022-108470. Epub 2022 Sep 27. PMID: 36167785; PMCID: PMC9685717.

12. Finnanger Garshol B, Knardahl S, Emberland JS, Skare Ø, Johannessen HA. Effects of the Labor Inspection Authority's regulatory tools on physician-certified sick leave and employee health in Norwegian home-care services - a cluster randomized controlled trial. Scand J Work Environ Health. 2024 Jan 1;50(1):28-38. doi: 10.5271/sjweh.4126. Epub 2023 Oct 30. PMID: 37903341; PMCID: PMC10924761.
13. Gray, W. B., & Mendeloff, J. (2023). Preventing construction deaths: The role of public policies. Regulation & Governance, 17(3), 726-754. https://doi.org/10.1111/rego.12486
14. Gründler R & Schulz R. Prävention macht stark – auch Deinen Rücken. Anlage 9: Bericht zur Ergebnisevaluation. Im Auftrag der GDA – Arbeitsprogramm MSE. 2018. WissensImpuls. Dresden. Deutschland.
15. Indregard AR, Knardahl S, Emberland JS, Skare Ø, Johannessen HA. Effectiveness of the Labour Inspection Authority's regulatory tools for work environment and employee health: study protocol for a cluster-randomised controlled trial among Norwegian home-care workers. BMJ Open. 2019 Nov 26;9(11):e031226. doi: 10.1136/bmjopen-2019-031226. PMID: 31772092; PMCID: PMC6886903.
16. Johannessen HA, Knardahl S, Emberland JS, Skare Ø, Finnanger Garshol B. Do regulatory tools instigate measures to prevent work-related psychosocial and ergonomic risk factors? A process evaluation of a Labour inspection authority trial in the Norwegian home-care services. BMC Res Notes. 2022 Nov 18;15(1):349. doi: 10.1186/s13104-022-06244-4. PMID: 36401320; PMCID: PMC9673432.
17. Johnson, M. S., Levine, D. I., & Toffel, M. W. (2023). Improving regulatory effectiveness through better targeting: Evidence from OSHA. *American Economic Journal: Applied Economics*, *15*(4), 30-67. doi: 10.1257/app.20200659
18. Kohlenprat, B. Mineralische Stäube im Bergbau. Abschlussbericht zur Schwerpunktation 2020 – 2022. (2023). Bundesministerium für Arbeit und Wirtschaft (BMAW). Sektion II – Arbeitsrecht und zentral – Arbeitsinspektorat. Wien. Österreich.
19. Lafuente, E., & Abad, J. (2021). Territorial efficiency: Analysis of the role of public work safety controls. *Safety science*, *134*, 105074. https://doi.org/10.1016/j.ssci.2020.105074
20. Lafuente, E., & Daza, V. (2020). Work inspections as a control mechanism for mitigating work accidents in Europe. Tec Empresarial, 14(1), 26-37. http://dx.doi.org/10.18845/te.v14i1.4953
21. Lamers J, Baars S. & Arbeitsprogrammleitung MSE. Abschlussbericht zum GDA-Arbeitsprogramm „Prävention macht stark – auch Deinen Rücken“ (Arbeitsprogramm MSE). 2018. Geschäftsstelle der Nationalen Arbeitsschutzkonferenz, Bundesanstalt für Arbeitsschutz und Arbeitsmedizin. Berlin.
22. Lee, J. M., & Taylor, L. O. (2019). Randomized safety inspections and risk exposure on the job: Quasi-experimental estimates of the value of a statistical life. *American Economic Journal: Economic Policy*, *11*(4), 350-374. doi: 10.1257/pol.20150024
23. Li, L. (2023). Helping Nurses or Hurting Patients? The Effect of Workplace Inspections in Nursing Facilities. *American Journal of Health Economics*, *9*(2), 205-230. https://doi.org/10.1086/722606
24. Li, L. (2022). Workplace Safety and Worker Productivity: Evidence from the MINER Act. ILR Review, 75(1), 117-138. https://doi.org/10.1177/0019793920931495 (Original work published 2020)
25. Li, L., & Singleton, P. (2018). The Effect of Workplace Inspections on Worker Safety. ILR Review, 72(3), 718-748. https://doi.org/10.1177/0019793918801575 (Original work published 2019)
26. Macpherson, R. A., & McLeod, C. B. (2022, July 6). An impact evaluation of the Infrastructure Health and Safety Association Certificate of Recognition (COR™) program. Partnership for Work, Health and Safety, School of Population and Public Health, University of British Columbia, Final report, pp. 1–52. https://www.ihsa.ca/pdfs/cor/impact-evaluation-ihsa-cor-program-full-report.pdf:

27. Macpherson, R. A., Yousefi, M., & McLeod, C. B. (2021). Determining hazard management changes in workplaces following workplace safety inspections by WorkSafeBC in British Columbia, Canada. *Safety Science*, *140*, 105298. https://doi.org/10.1016/j.ssci.2021.105298
28. McLeod CB, Macpherson RA, He A, Amick BC, Koehoorn M, Tompa E. The impact of regulatory workplace safety inspections on workers' compensation claim rates. Am J Ind Med. 2024 Oct;67(10):877-887. doi: 10.1002/ajim.23640. Epub 2024 Jul 25. PMID: 39051851.
29. McLeod, C., Macpherson, R., He, A., III, B. A., Tompa, E., & Koehoorn, M. (2021). The effectiveness of regulatory inspections in reducing work injuries in Alberta. Final report. https://open.alberta.ca/dataset/346a2597-dbe3-418d-846e-804ddd72f387/resource/78743157-717f-4d35-959f-4e337554ed47/download/jet-ohs-futures-effectiveness-reg-inspections-reducing-work-injuries-alberta-report.pdf
30. McLeod, K. V. (2019). *Understanding regulatory workplace safety inspections in British Columbia, Canada: Theory and evaluation* (Doctoral dissertation, University of British Columbia). https://dx.doi.org/10.14288/1.0386024
31. Mendeloff, J., Gray, W. B., Armour, P., & Neuhauser, F. (2021). The re-occurrence of violations in occupational safety and health administration inspections. *Regulation & Governance*, *15*(4), 1454-1479. https://doi.org/10.1111/rego.12315
32. Payá Castiblanque R. The Impact of the Direct Participation of Workers on the Rates of Absenteeism in the Spanish Labor Environment. Int J Environ Res Public Health. 2020 Apr 5;17(7):2477. doi: 10.3390/ijerph17072477. PMID: 32260481; PMCID: PMC7177909.
33. Raghunandan, A., & Ruchti, T. G. (2024). The impact of information frictions within regulators: evidence from workplace safety violations. *Journal of Accounting Research*, *62*(3), 1067-1120. https://doi.org/10.1111/1475-679X.12541
34. Tatsaki, E., Sgourou, E., Katsakiori, P., Konsta, I., & Gerasimou, S. (2019). The impact of occupational accidents and gross domestic product on the sanctions imposed by the Greek OSH Inspectorate. *Safety Science*, *115*, 349-352. Doi: https://doi.org/10.1016/j.ssci.2019.02.032
35. van der Molen HF, Basnet P, Hoonakker PL, Lehtola MM, Lappalainen J, Frings-Dresen MH, Haslam R, Verbeek JH. Interventions to prevent injuries in construction workers. Cochrane Database Syst Rev. 2018 Feb 5;2(2):CD006251. doi: 10.1002/14651858.CD006251.pub4. PMID: 29400395; PMCID: PMC6491133.
36. van der Molen, H.F. *et al.* (2019). Effectiveness of Interventions for Preventing Injuries in the Construction Industry: Results of an Updated Cochrane Systematic Review. In: Bagnara, S., Tartaglia, R., Albolino, S., Alexander, T., Fujita, Y. (eds) Proceedings of the 20th Congress of the International Ergonomics Association (IEA 2018). IEA 2018. Advances in Intelligent Systems and Computing, vol 825. Springer, Cham. https://doi.org/10.1007/978-3-319-96068-5_49
37. Weissbrodt, R., Arial, M., Graf, M., Iff, S., & Giauque, D. (2018). Preventing psychosocial risks at work: An evaluation study of labour inspectorate interventions. *Safety science*, *110*, 355-362. https://doi.org/10.1016/j.ssci.2018.08.024

5 Excluded studies

5.1 Intervention (n=15)

1. Antonelli, M. A., Castaldo, A., Forti, M., Marrocco, A., & Salustri, A. (2024). An alternative approach to understanding occupational safety and health (OSH): an empirical insurance cost perspective in Italy. *Applied Economics Letters*, 1–8. https://doi.org/10.1080/13504851.2024.2308564

2. Arewa AO, Theophilus S, Ifelebuegu A, Farrell P. Analysis of Penalties Imposed on Organisations for Breaching Safety and Health Regulations in the United Kingdom. Saf Health Work. 2018 Dec;9(4):388-397. doi: 10.1016/j.shaw.2018.01.004. Epub 2018 Feb 2. PMID: 30559986; PMCID: PMC6284184.
3. Behera, R. K., & Hassan, M. I. (2019). Regulatory interventions and industrial accidents: A case from India for 'Vision Zero'goals. *Safety science*, *113*, 415-424. https://doi.org/10.1016/j.ssci.2018.12.013
4. Bianchini, A., Donini, F., Pellegrini, M., & Saccani, C. (2017). An innovative methodology for measuring the effective implementation of an Occupational Health and Safety Management System in the European Union. *Safety Science*, *92*, 26-33.
5. https://doi.org/10.1016/j.ssci.2016.09.012
6. Bilgrami, A., Cutler, H., & Sinha, K. (2025). Higher penalties, broader definitions, and national standards: Did harmonized Australian workplace health and safety laws reduce workers' compensation receipt?. *Industrial Relations: A Journal of Economy and Society*, *64*(1), 77-102. https://doi.org/10.1111/irel.12357
7. Brouwer DH, Rees D. Can the South African Milestones for Reducing Exposure to Respirable Crystalline Silica and Silicosis be Achieved and Reliably Monitored? Front Public Health. 2020 Apr 7;8:107. doi: 10.3389/fpubh.2020.00107. PMID: 32318535; PMCID: PMC7154115.
8. Elsebaei, M. A., Elnawawy, O., Othman, A. A. E., & Badawy, M. (2021). A framework to activate the health and safety regulations in the Egyptian construction industry. *Journal of Engineering, Design and Technology*, *19*(5), 1158-1191.doi: 10.1108/JEDT-05-2020-0194
9. Hanson E, Boland M. Safety climate at agricultural cooperatives. J Safety Res. 2020 Dec;75:150-154. doi: 10.1016/j.jsr.2020.09.002. Epub 2020 Sep 22. PMID: 33334472.
10. Jafari A, De Azevedo Drummond P, Bhimani S, Nishigaya D, Ding AA, Farrell A, Maass KL. Enhancing detection of labor violations in the agricultural sector: A multilevel generalized linear regression model of H-2A violation counts. PLoS One. 2024 May 17;19(5):e0302960. doi: 10.1371/journal.pone.0302960. PMID: 38758737; PMCID: PMC11101028.
11. Jhang WG. The Vulnerability of Occupational Health and Safety to Deregulation: The Weakening of Information Regulations during the Economic Crisis in Korea. New Solut. 2018 May;28(1):151-168. doi: 10.1177/1048291117743781. Epub 2017 Nov 23. PMID: 29169305.
12. Karanikas, N., & Hasan, S. M. T. (2022). Occupational Health & Safety and other worker wellbeing areas: Results from labour inspections in the Bangladesh textile industry. *Safety Science*, *146*, 105533. https://doi.org/10.1016/j.ssci.2021.105533
13. Kim KW. Effect of an occupational health and safety management system based on KOSHA 18001 on industrial accidents. Work. 2021;68(2):449-460. doi: 10.3233/WOR-203385. PMID: 33522993.
14. Moon J. Missed reporting of nonfatal occupational injuries: estimation using the International Labor Organization datasets. Int Arch Occup Environ Health. 2022 Aug;95(6):1343-1356. doi: 10.1007/s00420-022-01892-2. Epub 2022 Jun 9. PMID: 35680671.
15. Sarazin P, Burstyn I, Kincl L, Friesen MC, Lavoué J. Characterization of the Selective Recording of Workplace Exposure Measurements into OSHA's IMIS Databank. Ann Work Expo Health. 2018 Mar 12;62(3):269-280. doi: 10.1093/annweh/wxy003. PMID: 29415273; PMCID: PMC6354673.

5.2 Outcome (n=13)

1. Appling XS, Lee P, Hedberg CW. Understanding the Relation between Establishment Food Safety Management and Salmonella Risk Factor Violations Cited during Routine Inspections. J Food Prot. 2019 Feb;82(2):339-343. doi: 10.4315/0362-028X.JFP-18-358. PMID: 30688536.

2. BinKhadim, S. A., & Zubair, M. (2024). A preliminary safety inspection methodology in the UAE using AIMS-PSA for risk assessment of APR1400 LOCA transients. *Progress in Nuclear Energy*, *169*, 105069.https://doi.org/10.1016/j.pnucene.2024.105069
3. Chao TK, Sun C, Beach JR. Developing a tool for identifying high-risk employers for inspection. Occup Med (Lond). 2017 Dec 2;67(8):609-614. doi: 10.1093/occmed/kqx136. PMID: 29016940.
4. Fagan KM, Hodgson MJ. Under-recording of work-related injuries and illnesses: An OSHA priority. J Safety Res. 2017 Feb;60:79-83. doi: 10.1016/j.jsr.2016.12.002. Epub 2016 Dec 16. PMID: 28160817.
5. Johnson, M. S. *2020.* "Regulation by Shaming: Deterrence Effects of Publicizing Violations of Workplace Safety and Health Laws." American Economic Review 110 (6): 1866–1904. DOI: 10.1257/aer.20180501
6. Makofske, M. P. (2019). Inspection regimes and regulatory compliance: How important is the element of surprise?. *Economics Letters*, *177*, 30-34.
7. https://doi.org/10.1016/j.econlet.2019.01.001
8. Makofske, M. P. (2021). Spoiled food and spoiled surprises: Inspection anticipation and regulatory compliance. *Journal of Economic Behavior & Organization*, *190*, 348-365. https://doi.org/10.1016/j.jebo.2021.07.036
9. Park S, Johnson MD, Hong O. Analysis of Occupational Safety and Health Administration (OSHA) noise standard violations over 50 years: 1972 to 2019. Am J Ind Med. 2020 Jul;63(7):616-623. doi: 10.1002/ajim.23116. Epub 2020 May 4. PMID: 32367510.
10. Sadeh, H., Mirarchi, C., Shahbodaghlou, F., & Pavan, A. (2023). Predicting the trends and cost impact of COVID-19 OSHA citations on US construction contractors using machine learning and simulation. *Engineering, Construction and Architectural Management*, *30*(8), 3461-3479. https://doi.org/10.1108/ECAM-10-2021-0953
11. Schell-Busey, N. Do extralegal variables impact the post-inspection process of the occupational safety and health administration?. *Crime Law Soc Change* 68, 187–216 (2017). **https://doi.org/10.1007/s10611-017-9681-7**
12. Steel J, Godderis L, Luyten J. Productivity estimation in economic evaluations of occupational health and safety interventions: a systematic review. Scand J Work Environ Health. 2018 Sep 1;44(5):458-474. doi: 10.5271/sjweh.3715. Epub 2018 Feb 6. PMID: 29405241.
13. Steel, E., Malinen, S. K., & Näswall, K. (2025). Improving work-related psychosocial risk management: A mixed methods evaluation of the factors influencing inspectors' practice. *Safety Science*, *181*, 106659. https://doi.org/10.1016/j.ssci.2024.106659

5.3 Form of publication (n=16)

1. Dobrolyubova, E. I. & Maslennikova, E. V. Evaluating the effectiveness of state regulatory enforcement and inspection activities in occupational safety: A sociological approach. TOMSK STATE UNIVERSITY JOURNAL, 2022: 475: 68-75 doi: 10.17223/15617793/475/9
2. Ho SH. Labour Inspection and Occupational Health during and post pandemic period. Saf Health Work. 2022 Jan;13:S76. doi: 10.1016/j.shaw.2021.12.977. Epub 2022 Feb 5. PMCID: PMC8817417.
3. King JC, Franklin RC, McBain-Rigg KE, et al. P4.017 Queensland Agriculture, Forestry and Fishing Work Health and Safety Prosecutions: Repercussions Injury Prevention 2021;27:A72. doi: 10.1136/injuryprev-2021-safety.220
4. Lazzarotto W, Pasqualini O, Farina E, Bena A. Efficacia dei controlli nei cantieri sulla riduzione degli infortuni: studio pilota di uno SPreSAL del Piemonte [Effectiveness of inspections in the construction sites on reducing innjuries: a pilot study in a Health Unit of Piedmont Region (Northern Italy)]. Epidemiol Prev. 2017 Mar-Apr;41(2):109-115. Italian. doi: 10.19191/EP17.2.P109.030. PMID: 28627152.

5. Li P, Li ML, Gao Y, Wang X. [Analysis on the detection of suspected occupational diseases and occupational contraindications for benzene workers in Tianjin]. Zhonghua Lao Dong Wei Sheng Zhi Ye Bing Za Zhi. 2022 Apr 20;40(4):283-287. Chinese. doi: 10.3760/cma.j.cn121094-20201012-00573. PMID: 35545596.
6. Magoha L, Nyanza EC, Asori M, Thomas DSK. Informal welders' occupational safety and environmental health risks in northwestern Tanzania. PLOS Glob Public Health. 2024 Feb 28;4(2):e0002923. doi: 10.1371/journal.pgph.0002923. PMID: 38416749; PMCID: PMC10901300.
7. Ruggiero N, Magna B, Cornaggia N, Rosa AM, Ferrero O, Mazzieri M, Consonni D, Cantoni S. Efficacia dei Servizi di Prevenzione e Sicurezza negli Ambienti di Lavoro (Servizi PSAL) neella riduzione degli infortune professionali in Regione Lombardia [Effectiveness of Health and Safety at Work Services (PSAL) in reducing occupational injuries in Lombardy Region]. Med Lav. 2018 Feb 20;109(2):110-124. doi: 10.23749/mdl.v109i2.6472. PMID: 29701627; PMCID: PMC7682179.
8. Samant, Y., Mattila-Wiro, P., Husberg, W., Falk, M., Knudesen, A. & Eyjolfur, S. Work today and in the future: Perspectives on Occupational Safety and Health challenges and opportunities for the Nordic labour inspectorates. Safety and Health at Work. 2022; 13. ISSN: 2093-7997 J9 - SAF HEALTH WORK-KR
9. Savina, A.D. & Antonenko, N. S. Implementing a risk-based approach in labour control and supervision activities. VOPROSY GOSUDARSTVENNOGO I MUNITSIPALNOGO UPRAVLENIYA-PUBLIC ADMINISTRATION ISSUES. 2024; 2: 157-182. Doi: 10.17323/1999-5431-2024-0-2-157-182
10. Spiegel A, Cummings KJ, Flattery J, Harrison R, Heinzerling A. Self-reported silica exposures and workplace protections among engineered stone fabrication workers in California. Am J Ind Med. 2022 Dec;65(12):1022-1024. doi: 10.1002/ajim.23432. Epub 2022 Oct 10. PMID: 36214615.
11. Surber SJ. OSHA Enforcement to Protect Health Care Workers From Violence. Am J Public Health. 2021 May;111(5):829-831. doi: 10.2105/AJPH.2021.306195. PMID: 33826390; PMCID: PMC8033990.
12. Tiesman H, Rojek J, Moore P, et al. (2017). 95 Injury prevention and law enforcement officers: how partnerships lead to impact Injury Prevention, 23:A35-A36. doi: 10.1136/injuryprev-2017-042560.95.
13. Vicente NE, Cordero DA Jr. The vital role of safety inspections in safeguarding occupational health in the post-COVID-19 era. J Public Health (Oxf). 2024 May 29;46(2):e306-e307. doi: 10.1093/pubmed/fdad231. PMID: 37955587.
14. Zaman, R. (2019). OSHA Implements Weighting System for Workplace Safety and Health Inspections. *COATINGSTECH*, *16*(11), 24-24. ISSN: 1547-0083 J9 - COATINGSTECH
15. Zhang WW, Li Y, Xiao H. [Research on supervision strategy of physical examination institutions after abolishing administrative approval]. Zhonghua Lao Dong Wei Sheng Zhi Ye Bing Za Zhi. 2018 Dec 20;36(12):941-942. Chinese. doi: 10.3760/cma.j.issn.1001-9391.2018.12.018. PMID: 30812088.
16. Zorina E. A., Metelkov A. N., Trapeznikova M. M. and Medvedeva A. A. (2023). Criminal and legal aspects of violation of labour rights in modern Russian and foreign legislation. VESTNIK TOMSKOGO GOSUDARSTVENNOGO UNIVERSITETA-PRAVO-TOMSK STATE UNIVERSITY JOURNAL OF LAW, 50, 43-61. doi: 10.17223/22253513/50/4

5.4 Study design (n=27)

1. Almond, P., & Esbester, M. (2018). Regulatory inspection and the changing legitimacy of health and safety. *Regulation & Governance*, *12*(1), 46-63. https://doi.org/10.1111/rego.12155

2. Arbury S, Collins NR, Magtahas J, Holmes M, Hodgson MJ. OSHA Workplace Violence Enforcement. J Occup Environ Med. 2022 Apr 1;64(4):e211-e216. doi: 10.1097/JOM.0000000000002482. Epub 2022 Jan 11. PMID: 35019893.
3. Blanc, F., Ottimofiore, G., & Myers, K. (2022). From OSH regulation to safety results: Using behavioral insights and a “supply chain” approach to improve outcomes–The experience of the health and safety Executive. *Safety Science*, *145*, 105491. https://doi.org/10.1016/j.ssci.2021.105491
4. Crook B, Willerton L, Smith D, Wilson L, Poran V, Helps J, McDermott P. Legionella risk in evaporative cooling systems and underlying causes of associated breaches in health and safety compliance. Int J Hyg Environ Health. 2020 Mar;224:113425. doi: 10.1016/j.ijheh.2019.113425. Epub 2019 Dec 24. PMID: 31978741.
5. Gómez-García, A. R., Chica, M. L. V., & García-Arroyo, J. A. (2023). Relationship between the territorial distribution of labor inspectors and work accident injuries: Clustering Ecuadorian provinces into four management scenarios. *Safety Science*, *158*, 105956. https://doi.org/10.1016/j.ssci.2022.105956
6. Hamid, A. R. A., Razak, A. R. A., Yusof, A. M., Jaya, R. P., Zakaria, R., Aminudin, E., ... & Rashid, I. A. (2019). Noncompliance of the occupational safety and health legislation in the Malaysian construction industry. In *IOP Conference Series: Earth and Environmental Science* (Vol. 220, No. 1, p. 012043). IOP Publishing. Doi: 10.1088/1755-1315/220/1/012043
7. Kamenska, I., Bokshyts, O., Prokhorchuk, P., Kalenskyi, A., Yevdokhovych, B., Bloshchynskyi, I., & Prokhorchuk, O. (2023). Labour Inspection as one of the main mechanisms for controlling occupational injuries. *Revista Romaneasca pentru Educatie Multidimensionala*, *15*(4), 88-101.
8. Kekkonen P, Reiman A, Väyrynen S, Rajala HK. Occupational safety and health in shared workplaces according to workplace inspection reports. Int J Occup Saf Ergon. 2021 Jun;27(2):504-516. doi: 10.1080/10803548.2019.1600314. Epub 2019 May 22. PMID: 30932739.
9. Keefe AR, Demers PA, Neis B, Arrandale VH, Davies HW, Gao Z, Hedges K, Holness DL, Koehoorn M, Stock SR, Bornstein S. A scoping review to identify strategies that work to prevent four important occupational diseases. Am J Ind Med. 2020 Jun;63(6):490-516. doi: 10.1002/ajim.23107. Epub 2020 Mar 29. Erratum in: Am J Ind Med. 2020 Dec;63(12):1169. doi: 10.1002/ajim.23189. PMID: 32227359.
10. Kica J, Rosenman KD. Multi-source surveillance for work-related crushing injuries. Am J Ind Med. 2018 Feb;61(2):148-156. doi: 10.1002/ajim.22800. Epub 2017 Dec 4. PMID: 29205424.
11. Konstantinou E, Grendova K, Maragaki E, Klimešová M, Ošmerová L, Evanthia S. Health and Safety Inspections in Workplaces: a Review Among Three European Countries. Mater Sociomed. 2020 Sep;32(3):235-242. doi: 10.5455/msm.2020.32.235-242. PMID: 33424455; PMCID: PMC7780825.
12. Li X, Zhang X, Liu Q, Zhang Y, Gu X, Qiu Z. Research on Coal Mine Building Compliance Inspection System Based on Accident Causation and BIM in China. Int J Environ Res Public Health. 2022 Dec 8;19(24):16466. doi: 10.3390/ijerph192416466. PMID: 36554347; PMCID: PMC9778409.
13. Liebman A, Franko E, Reyes I, Keifer M, Sorensen J. An overview and impact assessment of OSHA large dairy local emphasis programs in New York and Wisconsin. Am J Ind Med. 2018 Jun 21. doi: 10.1002/ajim.22868. Epub ahead of print. PMID: 29926466.
14. Lim S, Oh AR, Won JH, Chon JJ. Improvement of inspection system for reduction of small-scale construction site accident in Korea. Ind Health. 2018 Nov 21;56(6):466-474. doi: 10.2486/indhealth.2018-0033. Epub 2018 Jun 23. PMID: 29937474; PMCID: PMC6258752.
15. Linnan LA, Leff MS, Martini MC, Walton AL, Baron S, Hannon PA, Abraham J, Studer M. Workplace health promotion and safety in state and territorial health departments in the United States: a national mixed-methods study of activity, capacity, and growth

opportunities. BMC Public Health. 2019 Mar 12;19(1):291. doi: 10.1186/s12889-019-6575-x. PMID: 30866884; PMCID: PMC6417036.
16. Lösch R, Amler N, Drexler H. Arbeits- und Gesundheitsschutz und Betriebliches Eingliederungsmanagement in Deutschland – Ein systematisches Review zum Umsetzungsstand gesetzlicher Vorgaben [Occupational Safety and Health and Company Integration Management in Germany - A Systematic Review on Implementation of Legal Requirements]. Gesundheitswesen. 2022 May;84(5):422-437. German. doi: 10.1055/a-1354-6227. Epub 2021 Mar 15. PMID: 33723831.
17. Lotfollahzadeh, A., Ghaderi, A., Grossi, B., & Zeghardi, B. S. (2018). Presentation of a New Inspection Structure to Solve HSE problems in industrial towns. *Pakistan Journal of Medical and Health Sciences*, *12*(4), 1836-1838.
18. Lowe, B. D., Albers, J., Hayden, M., Lampl, M., Naber, S., & Wurzelbacher, S. (2020). Review of construction employer case studies of safety and health equipment interventions. *Journal of construction engineering and management*, *146*(4), 04020012. doi: https://doi.org/10.1061/(ASCE)CO.1943-7862.000178
19. Lyutov, N., & Voitkovska, I. (2021). Labour Inspections in Post-Soviet Countries: Reform or Collapse?. *International Journal of Comparative Labour Law and Industrial Relations*, *37*(2/3). https://doi.org/10.54648/ijcl2021013
20. Michaels D, Barab J. The Occupational Safety and Health Administration at 50: Protecting Workers in a Changing Economy. Am J Public Health. 2020 May;110(5):631-635. doi: 10.2105/AJPH.2020.305597. Epub 2020 Mar 19. PMID: 32191515; PMCID: PMC7144438.
21. Nielsen, K. J. (2016). A comparison of inspection practices within the construction industry between the Danish and Swedish Work Environment Authorities. *Construction Management and Economics*, *35*(3), 154–169. https://doi.org/10.1080/01446193.2016.1231407
22. Popple, S., Way, K., Johnstone, R., Croucher, R., & Miller, P. (2023). A comparative analysis of Inspector responses to complaints about psychosocial and physical hazards. *Regulation & governance*, *17*(1), 234-249. https://doi.org/10.1111/rego.12447
23. Rikhotso O, Morodi TJ, Masekameni DM. Occupational Health Hazards: Employer, Employee, and Labour Union Concerns. Int J Environ Res Public Health. 2021 May 19;18(10):5423. doi: 10.3390/ijerph18105423. PMID: 34069469; PMCID: PMC8159080.
24. Surasi, K., Ballen, B., Weinberg, J. L., Materna, B. L., Harrison, R., Cummings, K. J., & Heinzerling, A. (2022). Elevated exposures to respirable crystalline silica among engineered stone fabrication workers in California, January 2019–February 2020. *American Journal of Industrial Medicine*, *65*(9), 701-707. https://doi.org/10.1002/ajim.23416
25. Tang, K. H. D. (2020). A comparative overview of the primary Southeast Asian safety and health laws. *International Journal of Workplace Health Management*, *13*(6), 601-632. doi: https://doi.org/10.1108/IJWHM-10-2019-0132
26. Viscusi, W. K., & Cramer, R. J. (2023). How regulations undervalue occupational fatalities. *Regulation & Governance*, *17*(1), 250-271. Doi: https://doi.org/10.1111/rego.12445
27. Weissbrodt, R., & Giauque, D. (2017). Labour inspections and the prevention of psychosocial risks at work: A realist synthesis. *Safety Science*, *100*, 110-124. https://doi.org/10.1016/j.ssci.2017.02.012

5.5 Duplicate (n=1)

1. Finnanger Garshol B, Knardahl S, Emberland JS, Skare Ø, Johannessen HA. Effects of the Labor Inspection Authority's regulatory tools on physician-certified sick leave and employee health in Norwegian home-care services - a cluster randomized controlled trial. Scand J Work Environ Health. 2024 Jan 1;50(1):28-38. doi: 10.5271/sjweh.4126. Epub 2023 Oct 30. PMID: 37903341; PMCID: PMC10924761.

6 Data extraction tables

6.1 Systematic reviews

<table>
<tr><th>General</th><th>Characteristics of systematic review</th><th>Results
Descriptive and quantitative summary</th><th>Comments</th></tr>
<tr><td>First Author, Year
(#)

Extracted by:

Checked by:</td><td>Search period:

Database searched: (electronic databases searched, grey literature)

Eligibility criteria: (description of inclusion criteria)

Number of studies on inspections included: n=

Number of studies on inspections published since 2017 included: n=</td><td>Descriptive summary: (descriptive statistics, study quality)

Quantitative (meta-analytical) results: (prevalence/ Incidence, Relative Risk, Odds Ratio, Risk of bias, GRADE (Grading of Recommendations, Assessment, Development, and Evaluations))

Conclusion of systematic review: (as stated by authors of the review)</td><td>Funding:
(source)

Conflict of Interest stated:

Other comments:</td></tr>
<tr><td>Andersen, 2019
#179

Extracted by:
MS

Checked by:
UBA</td><td>Search period: 1966–2017 (February)

Database searched: PubMed, Web of science, HSELINE and NIOSHTIC-2 from OSH UPDATE

Grey literature: HSELINE, NIOSHTIC-2 and OSH UPDATE

Eligibility criteria: a) English or Scandinavian, published between 1966 and February 2017 in a peer-reviewed journal, report or on a website (grey literature); b) investigating work environment issues; c) intervention involving OSH authorities, such as inspections,</td><td>Descriptive summary:
<u>Quality of included studies:</u> (used tool from Tompa et al.[1]) 14 high and 9 medium quality studies
<u>Location of included studies:</u> USA (n=17), Italy (n=2), Sweden (n=2) and Canada (n=2)
<u>Outcomes:</u> injuries (n=14), worker compensation claims (n=2), compliance with OSH regulation and violations (n=5)
Noise awareness and compliance (n=1), reducing workload and employment status (n=1)
<u>Inspection:</u> inspection sequence (n=3), general inspection deterrence and specific deterrence with/without penalties (n=1), consultative activities (n=3), and supportive inspection (n=1)

[1]Tompa E, Trevithick S, McLeod C. Systematic review of the prevention incentives of insurance and regulatory mechanisms for occupational health and safety. Scand J Work Environ Health. 2007 Apr;33(2):85-95. doi: 10.5271/sjweh.1111. PMID: 17460796.

Quantitative (meta-analytical) results:

Effect of inspection on injuries, compliance and compensation claims (random effect model)<table><tr><th>Outcome</th><th>OR</th><th>95% CI</th></tr></table></td><td>Funding:
Danish Working Environment Authority (J.nr. 20150077671)

Conflict of Interest stated:
yes

Other comments:-</td></tr>
</table>

<table>
<tr><th>General</th><th>Characteristics of systematic review</th><th>Results
Descriptive and quantitative summary</th><th>Comments</th></tr>
<tr><td></td><td>training, campaigns or the introduction of technical device; d) studying relevant outcome e.g., injuries, fatalities, occupational exposure, sick leave, work-related symptoms and disease and compliance; e) study design: control group or a reference such as before-after comparisons or other types of controlled designs (but also inclusion of before-after design without a control group); f) providing quantitative measures or data for calculation of risk estimates and 95% confidence interval

Number of studies on inspections included: n=23

Number of studies on inspections published since 2017 included: n=0</td><td><table><tr><td>Injuries</td><td>0.83</td><td>0.73–0.93</td></tr><tr><td>Compliance</td><td>0.65</td><td>0.50–0.83</td></tr><tr><td>Compensation claims</td><td>0.96</td><td>0.94–0.98</td></tr></table>CI=confidence interval; OR= odds ratio

- initial inspections, follow-up inspections, and complaint and accident- prompted inspections -> higher compliance rates in comparison to any other type of inspections (no quantitative results shown)
-inspections with citations or with more penalties -> fewer injuries and higher compliance (no quantitative results shown)

<u>Risk of Bias:</u> (funnel plots) large heterogeneity for compliance; no publication bias for injuries and compensation claims)
<u>GRADE:</u> (modified version used; strength of evidence: strong (++++), moderately strong (+++0), limited (++00) and insufficient (+000)) ->moderately strong evidence for the effect of inspections on injuries and compliance with OHS legislation, and low evidence for compensation claims

Conclusion of systematic review:
“The results from this review indicate that [..] workplace inspections with or without penalties are indeed effective means to improve the working environment.”</td><td></td></tr>
<tr><td>
First Author, Year
(#)
Bondebjerg, 2023
#2202

(study protocol: Bondebjerg, 2022, #2658)

Extracted by:
MS

Checked by:</td><td>Search period: until January 2022; hand search from 2015-December 2022

Database searched: Academic Search, EconLit, PsycINFO, SocINDEX, CINAHL, International Bibliography of the Social Sciences, Sociological Abstracts, Science Citation Index Expanded, Social Sciences Citation Index, MEDLINE, ERIC

Grey literature: Hand search in 16 journals; dissertations from EBSCO Open Dissertations, working papers/conference proceedings (n=5), websites of governmental</td><td>Descriptive summary:
evidence and gap map (EGM)
<u>Study design of included studies</u> (n= 21 studies): RCTs: n=5 (one ongoing study), NRSs: n=14 (one study is both an RCT and a NRS -> counted in both categories), systematic reviews: n=3
<u>Study quality of primary studies:</u> not determined
<u>Study quality of systematic reviews:</u> AMSTAR: results not specified: “three systematic reviews received a ‘critically low’ rating, one received a ‘low’ rating, and two did not receive a full rating due to them not containing studies eligible for this EGM”
<u>Outcome</u>: injuries (n=15 studies, one study is counted as both an NRS and an RCT), compliance (n=11, one study is counted as both an NRS and an RCT), health (n=3, one study is counted as both an NRS and an RCT), work-related exposures (n=3), and finally sickness absence (n=1).
<u>Intervention:</u> inspection and sanctions

Quantitative (meta-analytical) results: No quantitative analysis

Conclusion of systematic review:</td><td>Funding:
internal source: VIVE, The Danish Center for Social Science Research, Denmark; external source: no support

Conflict of Interest stated:
yes</td></tr>
</table>

General	Characteristics of systematic review	Results<br>Descriptive and quantitative summary	Comments
	institutions, international organisations, online research repositories and Google (n=23), EGMs and systematic reviews (n=10), trial registries (n=4), citation-tracking (forward and backwards), contact of experts<br><br>**Eligibility criteria:** a) focus: formulation of regulatory standards, incentives for compliance, inspection by regulatory agencies, enforcement by regulatory agencies (sanctions), information, guidance, and consulting, and training initiatives; b) study design: Randomised controlled trials (RCTs), Non-randomised studies (NRS) with comparison of two or more groups of participants, at least two organisations/companies/workplaces in both the treatment and comparison group, only studies with sharp regression discontinuity designs (RDD) systematic reviews, exclusion of studies: with instrumental variables, fuzzy regression RDD, qualitative research; c) only interventions from environment regulatory authorities or regulatory agencies and other organisations authorised as regulators; d) workers aged >15 years with workplace in OECD countries; e) outcome: workplace compliance with regulatory standards (intermediate outcome); work-related exposures	"To sum up, there is an evidence base available concerning the effects of inspection activity on injury incidence. "<br>"One suggestion based on the evidence located with this map would be to carry out a focused systematic review and meta-analysis of the effects of inspection activity since this domain is relatively well-populated by primary effect studies."	**Other comments:**-

<table>
<tr><th>General</th><th>Characteristics of systematic review</th><th>Results
Descriptive and quantitative summary</th><th>Comments</th></tr>
<tr><td></td><td>(intermediate outcome); health (final outcome); incidence of work-related injuries (final outcome), sickness absence (final outcome), f) hand search: studies published in English, German, French, Danish, Norwegian, and Swedish

Number of studies on inspections included: n=21

Number of studies on inspections published since 2017 included: n=5</td><td></td><td></td></tr>
<tr><td>First Author, Year
(#)

Dyreborg, 2022
#2026

Extracted by:
MS

Checked by:</td><td>Search period: until July 2015

Database searched: PubMed, Embase, CINAHL, OSH ROM (NIOSHTIC, HSELINE, CIS-DOC), PsycINFO, EconLit, Web of Science, ProQuest

Grey literature search: websites of organisations (n=8), OSH ROM and Google (first 100 hits).

Eligibility criteria: a) study design: RCTs (including studies with cluster randomization), quasi randomized studies, controlled before and after studies, studies with least three measures (if at least one measure was before the intervention), single group study designs; b) participants: working
Populations; c) intervention: all types of safety interventions addressing accidents at work; d) outcome:</td><td>Descriptive summary:
Effects of enforcement: n=7 studies (representing 12 interventions) assessed effects of inspections and enforcement of laws and regulations
<u>Study design:</u> RCT: n=1, nRCTs: n=6
<u>Study quality:</u> Risk of bias assessed (EPOC Review group)

Quantitative (meta-analytical) results:
Summary of the effect of enforcement and compliance.
Tab. 20: Results of meta-analysis for outcome injuries
<table>
<tr><td rowspan="2">Number of safety interventions
Types of safety interventions and follow-up periods</td><td colspan="3">Quality assessment</td><td rowspan="2">Level of evidence[a]
RCT & CBA</td><td rowspan="2">Strength of effect
RCT & CBA</td><td colspan="2">Meta-analysis Estimate (95% CI)</td><td rowspan="2">I^{2b}</td></tr>
<tr><td>High</td><td>Moderate</td><td>Low</td><td>RCT</td><td>CBA</td></tr>
<tr><td colspan="9">Enforcement of laws and regulations</td></tr>
<tr><td>Short-term (−12 months)</td><td>1</td><td></td><td></td><td>limited</td><td>little</td><td></td><td>OR=0.86 (0.77-0.95)</td><td>NA</td></tr>
<tr><td>Medium-term (12–36 months)</td><td>2</td><td></td><td>4</td><td>moderate</td><td>None to little</td><td>0.99 (0.89-1.10)</td><td>OR=0.95 (0.93-0.97)</td><td>NA/ 0%</td></tr>
<tr><td>Long-term (36–month)</td><td>4</td><td></td><td></td><td>strong</td><td>little</td><td></td><td>0.96 (0.93-0.98)</td><td>0%</td></tr>
<tr><td colspan="9">Enforcement of laws w/penalties</td></tr>
<tr><td>Medium-term (12–36 months)</td><td>2</td><td></td><td></td><td>moderate</td><td>None to little</td><td></td><td>0.95 (0.92-0.98)</td><td>0%</td></tr>
</table></td><td>Funding:
internal: National Research Centre for the Working Environment Denmark,
external: Working Environment Research Fund, Denmark (Grant project number: 48-2010-09)

Conflict of Interest stated:
yes

Other comments:-</td></tr>
</table>

<table>
<tr><th>General</th><th>Characteristics of systematic review</th><th>Results
Descriptive and quantitative summary</th><th>Comments</th></tr>
<tr><td></td><td>primary: incidence of accidental work injuries causing physical harm, Number of lost working days due to injury events and cases of work disability, secondary: knowledge, attitudes, changes in workplace norms, climate, or culture; e) no language or date restrictions

Number of studies on inspections included: n=7

Number of studies on inspections published since 2017 included: n=0</td><td>CBA= controlled before-after study; CI= confidence interval; NA=not applicable; RCT=randomized-controlled trial
[a]Level of evidence= modified version of Tompa, Trevithick, et al. (2007): strong=effect size supported by ≥3 high-quality studies reporting consistent findings; moderate= effect size supported by ≥2 high-quality studies or≥3 medium quality reporting consistent findings, limited= effect size supported by ≥1 high-quality studies or≥2 medium quality reporting consistent findings
[b]= I2, Heterogeneity

Conclusion of systematic review: “We conclude limited evidence for a little effect of enforcement and compliance efforts at short-term follow-up and strong evidence for a little effect at long-term follow-up and moderate evidence of no or little effect at medium-term follow-up.”</td><td></td></tr>
<tr><td>First Author, Year
(#)

Van der Molen, 2018
#103

Update
Van der Molen, 2019
#3162

Extracted by:
MS

Checked by:</td><td>Search period: until April 2017

Database searched: Cochrane Injuries Group's specialised register, Cochrane Central Register of Controlled Trials (CENTRAL; 2017 Issue 3), MEDLINE (from 1966), Embase (from 1988), PsycINFO (from 1983)

Eligibility criteria: a) study population: construction workers (company or self-employed workers); b) outcome: primary outcome: fatal or non-fatal occupational injuries, secondary outcome: lost working days, behavioural chnages; c) study design: Randomised controlled trials (RCTs), cluster-randomised controlled trials (cRCTs), controlled before-after (CBA) and interrupted time series (ITS) studies, randomised and non-randomised ITS and CBA; ITS: at least 3 time points; CBA:</td><td>Descriptive summary:
-safety inspections:
one ITS study on safety inspections and sanctions for violations on non-fatal injuries in Italy
-risk of bias of ITS studies assessed according to guideline by EPOC Review Group

Quantitative (meta-analytical) results:
Inspection versus no inspection of construction site:
Tab. Summary of findings 5
Population: workers of reconstruction site
Settings: reconstruction site after earthquake
Intervention: accidents in building site
Comparison: no inspection
<table>
<tr><th>Outcomes</th><th>Impact[a] (95% CI)</th><th>No of studies</th><th>Quality of the evidence (GRADE)</th></tr>
<tr><td>Fatal injuries</td><td>No available data</td><td></td><td></td></tr>
<tr><td>Non-fatal injuries
Change in level</td><td>Effect size 0.07 (−2.83 to 2.97)</td><td>1 ITS</td><td>⊕⊕⊖⊖
Very low[b]</td></tr>
<tr><td>Non-fatal injuries
Change in slope</td><td>Effect size 0.63 (−0.35 to 1.61)</td><td>1 ITS</td><td>⊕⊕⊖⊖
Very low[b]</td></tr>
</table>
CI=confidence interval; ITS=interrupted time series.
[a]Effect size interpretation: <0.2: small effect small effect, 0.2-0.8 moderate effect, > 0.8: large effect; negative sign means decrease in injuries
[b]Observational studies start with low-quality evidence; we downgraded with two levels: 1 for risk of bias and 1 for imprecision (1 study)

GRADE Working Group grades of evidence
High quality: further research is very unlikely to change our confidence in the estimate of effect.</td><td>Funding:
performed for Cochrane Database of Systematic Reviews

Conflict of Interest stated:
NR

Other comments:-</td></tr>
</table>

General	Characteristics of systematic review	Results<br>Descriptive and quantitative summary	Comments
	outcome measured before and after in intervention and control group (also searching before-after studies without a control group and retrospective cohort studies for discussion); d) no restriction of language or publication status<br><br>**Number of studies on inspections included:** n=1<br><br>**Number of studies on inspections published since 2017 included:** n=0	Moderate quality: further research is likely to have an important impact on our confidence in the estimate of effect and may change the estimate.<br>Low quality: further research is very likely to have an important impact on our confidence in the estimate of effect and is likely to change the estimate.<br>Very low quality: we are very uncertain about the estimate.<br><br>**Conclusion of systematic review:** "Nor is there sufficient evidence in the included studies that regionally oriented safety interventions such as campaigning, training, inspections or new occupational health services are effective for reducing non-fatal injuries in construction workers."	

6.2 Epidemiological studies

General	Study	Population	Exposure	Outcome	Results	Comments
**First Author, Year**<br>**#**<br><br>**Extracted by:**<br><br>**Checked by:**	**Study name:**<br><br>**Country:**<br><br>**Study design:** *(e.g. cross-sectional, cohort, case-cohort, case-control, experimental*	**Recruitment method/ Data source:**<br><br>**Matching criteria:** *(case-cohort, case control only)*<br><br>**# invited companies:**<br><br>**# companies at baseline:** *(all individuals participating at baseline)*<br><br>**Response:** *(number of invited/ number participated at baseline in %)*<br><br>**# companies at follow –up:**	**Description of intervention:**<br><br>**Study groups:**<br><br>**Time of measurement:**	**Outcome name:**<br><br>**Outcome definition and assessment:**<br><br>**Time of measurement:**	*Description as stated in the paper.*<br>*(Calculation performed by the review team, should be noted. Unpublished results obtained by personal communication should be noted.)*<br><br>**Descriptive Statistics:**<br><br>**Statistical methods used** *(Models used; adjustment sets)*:<br><br>**Unadjusted/ adjusted estimates with precision (e.g. 95% confidence interval) for each outcome** *(with number analysed)*:<br><br>**Short summary of findings:**	**Funding:** *(source)*<br><br>**Conflict of Interest stated:**<br><br>**Other comments:**

<table>
<tr><th>General</th><th>Study</th><th>Population</th><th>Exposure</th><th>Outcome</th><th>Results</th><th>Comments</th></tr>
<tr><td></td><td>study, lab study)

Time of Study: (month, year(s))</td><td>Loss-to-follow-up: (e.g. number of participated at baseline / number participated at follow-up X in %):

Work characteristics: (e.g. industry, job type)</td><td></td><td></td><td></td><td></td></tr>
<tr><td>Weissbrodt, 2018 #2920

Extracted by: MS

Checked by: UBA</td><td>Study name: -

Country: Switzerland

Study design: before-after study with control group

Time of Study: 2014-2018</td><td>Recruitment method/ Data source:
Swiss cantonal labour inspectorates participated in inspection campaign
Inspected=Firms in which inspection was planned and announced to researchers: n=486; Non-inspected=stratified random sample drawn from federal Business and Enterprise Register: n=429
-> 2 questionnaire surveys at 1-year interval: questionnaire answered by person best knowing about OSH (either vis structured phone interview or paper version); a manager in almost 90% of cases

Matching criteria:
Control group: stratified by language region, size, sector and structure (single – multi-branch companies) to match intervention group.</td><td>Description of intervention: inspection campaign on psychological risk at work from State Secretariat for Economic Affairs (SECO): SECO organised specific courses for inspectors, gave guidance and events for inspectors, employers and OHS specialists

Study groups:
<u>Intervention group:</u> Inspectors</td><td>Outcome name: Preventing psychological risks (PSR)

Outcome definition and assessment: Questionnaire survey (intervention group: short online survey if psychological issues were addressed during visit by inspector + type of action)

Scales of questionnaire (scores 0-12):
- OHS management (8 items)
-Worker participation (6 items)</td><td>Descriptive Statistics:
No difference in branch, size and economic situation between inspected and non-inspected companies. Addressing PSR in 97.2% of workplace visits. Prevention measures against PSR in 49.4%

Tab1: Comparison of the median scores between the inspected and non-inspected companies on the 6 single-factor scales, at the first and second surveys (N=346).
<table>
<tr><td>Scales</td><td colspan="2">Comparison of median scores 1rst survey</td><td colspan="2">Comparison of median scores 2nd survey</td></tr>
<tr><td></td><td>Non-Insp</td><td>Insp</td><td>Non-Insp</td><td>Insp</td></tr>
<tr><td>OHS management</td><td>8.0</td><td>9.0</td><td>8.5</td><td>10.0*</td></tr>
<tr><td>Worker participation</td><td>5.3</td><td>6.7*</td><td>6.7</td><td>7.3*</td></tr>
<tr><td>Willingness to prevent PSR</td><td>9.6</td><td>9.6</td><td>8.8</td><td>10.4*</td></tr>
<tr><td>Specific PSR management</td><td>4.4</td><td>6.0*</td><td>5.6</td><td>7.2*</td></tr>
<tr><td>Improvement of working conditions</td><td>7.3</td><td>8.0</td><td>8.0</td><td>8.0</td></tr>
<tr><td>Ability in PSR prevention</td><td>4.0</td><td>5.5*</td><td>4.5</td><td>7.0*</td></tr>
</table>
OHS=occupational health and safety. PSR= psychosocial risks.* p < .05

Statistical methods used: regression models Dependent variables = scores from second survey. Predictors = interaction terms between visits, economic sector (secondary vs tertiary) and company size (<100 vs ≥ 100 employers). Economic situation was excluded (reason: p>0.25)</td><td>Funding:
State Secretariat for Economic Affairs and the Federal Coordination Commission for Occupational Safety, Switzerland

Conflict of Interest stated: yes

Other comments:
No ethical approval (reason: no personal data collected, SECO has legal prerogative to collect information at workplace)
- only person best knowing OSH was interviewed
- baseline differences between inspected and non-inspected firms: higher scores for intervention group for worker participation, specific</td></tr>
</table>

<table>
<tr><th>Genera l</th><th>Study</th><th>Population</th><th>Exposure</th><th>Outcome</th><th>Results</th><th>Comments</th></tr>
<tr>
<td></td>
<td></td>
<td># invited companies:
Inspected: n=287
Non-inspected: n=405

companies at baseline:
Inspected: n=208
Non-inspected: n=197

Response:
Inspected: 72.5%
Non-inspected: 48.6%

companies at follow –up:
firms participating in 2nd survey
Inspected: n=185
Non-inspected: n=161

Loss-to-follow-up:
Inspected: 11.1%
Non-inspected: 18.3%

Work characteristics:
economic sector (secondary (industry*) and tertiary: service sector*)
<table>
<tr><td></td><td colspan="2">inspected</td><td colspan="2">Non-inspected</td></tr>
<tr><td></td><td>II</td><td>III</td><td>II</td><td>III</td></tr>
<tr><td>< 100</td><td>6.0 %</td><td>20. 5%</td><td>6.6 %</td><td>20 .2 %</td></tr>
<tr><td>≥ 100</td><td>3.2 %</td><td>23. 7%</td><td>3.6 %</td><td>16 .2 %</td></tr>
</table>
economic sector: 2= secondary; 3 = tertiary</td>
<td>conducted systematic visits. Inspectors informed investigators of every planned visit -> company could be contacted before visit
<u>Control group:</u> No intervention performed during study period

Time of measurement:
1 visit during study
(for 1 year: inspectors informed investigators in advance of every planned visit ->company could be contacted beforehand)</td>
<td>- Willingness to prevent (5 items)
- Specific PSR management (10 items)
- Improvement of working conditions (6 items)
- Ability in PSR prevention (8 items)

Time of measurement:
one-year interval</td>
<td>Unadjusted/ adjusted estimates with precision (e.g. 95% confidence interval) for each outcome:

<u>Tab. 2: robust regression models (</u>Increase per 1 unit of scale)

OHS management:
<table>
<tr><td></td><td>Scores 2nd survey with standard error</td></tr>
<tr><td>Score 1rst survey</td><td>0.63*** (0.05)</td></tr>
<tr><td>Interaction terms</td><td></td></tr>
<tr><td>NI/tertiary/≥100</td><td>0.65 (0.38)</td></tr>
<tr><td>NI/secondary/<100</td><td>0.91* (0.40)</td></tr>
<tr><td>NI/secondary/≥100</td><td>1.79*** (0.41)</td></tr>
<tr><td>I/tertiary/<100</td><td>2.02*** (0.37)</td></tr>
<tr><td>I/tertiary/≥100</td><td>1.61*** (0.33)</td></tr>
<tr><td>I/secondary/<100</td><td>1.90** (0.41)</td></tr>
<tr><td>I/secondary/≥100</td><td>1.86*** (0.41)</td></tr>
<tr><td>Intercept</td><td>2.66*** (0.41)</td></tr>
<tr><td>Robust R^2_w</td><td>0.75</td></tr>
</table>
No. of observations: 346. Standard errors are reported in parentheses. *p<0.05, **p<0.01, ***p <0.001. OHS: occupational health and safety. PSR: psychosocial risks. NI: non-inspected. I: inspected. Reference value for interaction terms: NI/tertiary/<100.

Worker participation:
<table>
<tr><td></td><td>Scores 2nd survey with standard error</td></tr>
<tr><td>Score 1rst survey</td><td>0.69*** (0.05)</td></tr>
<tr><td>Interaction terms</td><td></td></tr>
<tr><td>NI/tertiary/≥100</td><td>0.29 (0.38)</td></tr>
<tr><td>NI/secondary/<100</td><td>-0.46 (0.50)</td></tr>
<tr><td>NI/secondary/≥100</td><td>1.49** (0.51)</td></tr>
<tr><td>I/tertiary/<100</td><td>0.08 (0.37)</td></tr>
<tr><td>I/tertiary/≥100</td><td>0.81* (0.38)</td></tr>
<tr><td>I/secondary/<100</td><td>0.61 (0.57)</td></tr>
<tr><td>I/secondary/≥100</td><td>0.70 (0.57)</td></tr>
<tr><td>Intercept</td><td>2.31*** (0.34)</td></tr>
<tr><td>Robust R^2_w</td><td>0.34 (0.63)</td></tr>
</table>
No. of observations: 346. Standard errors are reported in parentheses. *p<0.05, **p<0.01, ***p <0.001. OHS: occupational health and safety. PSR: psychosocial risks. NI: non-inspected. I: inspected. Reference value for interaction terms: NI/tertiary/<100.

Willingness to prevent
<table>
<tr><td></td><td>Scores 2nd survey with standard error</td></tr>
<tr><td>Score 1rst survey</td><td>0.53*** (0.4)</td></tr>
<tr><td>Interaction terms</td><td></td></tr>
<tr><td>NI/tertiary/≥100</td><td>0.42 (0.38)</td></tr>
<tr><td>NI/secondary/<100</td><td>-1.46** (0.53)</td></tr>
</table></td>
<td>PSR management and ability in PSR prevention</td>
</tr>
</table>

<table>
<tr><th>General</th><th>Study</th><th>Population</th><th>Exposure</th><th>Outcome</th><th>Results</th><th>Comments</th></tr>
<tr>
<td></td>
<td></td>
<td>* https://www.bfs.admin.ch/bfs/en/home/statistics/work-income/employment-working-hours/labour-force-characteristics/economic-sector.html</td>
<td></td>
<td></td>
<td>
<table>
<tr><td>NI/secondary/≥100</td><td>0.01 (0.41)</td></tr>
<tr><td>I/tertiary/<100</td><td>0.96** ((0.33)</td></tr>
<tr><td>I/tertiary/≥100</td><td>1.05*** (0.30)</td></tr>
<tr><td>I/secondary/<100</td><td>0.94 (0.54)</td></tr>
<tr><td>I/secondary/≥100</td><td>1.15 (0.80)</td></tr>
<tr><td>Intercept</td><td>4.23*** (0.49)</td></tr>
<tr><td>Robust R^2_w</td><td>0.58</td></tr>
</table>
No. of observations: 346. Standard errors are reported in parentheses. *p<0.05, **p<0.01, ***p <0.001. OHS: occupational health and safety. PSR: psychosocial risks. NI: non-inspected. I: inspected. Reference value for interaction terms: NI/tertiary/<100.

Specific PSR management
<table>
<tr><td></td><td>Scores 2nd survey with standard error</td></tr>
<tr><td>Score 1rst survey</td><td>0.72*** (0.04)</td></tr>
<tr><td>Interaction terms</td><td></td></tr>
<tr><td>NI/tertiary/≥100</td><td>0.81 (0.49)</td></tr>
<tr><td>NI/secondary/<100</td><td>-0.32 (0.45)</td></tr>
<tr><td>NI/secondary/≥100</td><td>1.08* (0.48)</td></tr>
<tr><td>I/tertiary/<100</td><td>0.73 (0.43)</td></tr>
<tr><td>I/tertiary/≥100</td><td>1.36** (0.50)</td></tr>
<tr><td>I/secondary/<100</td><td>-0.39 (0.51)</td></tr>
<tr><td>I/secondary/≥100</td><td>1.32 (0.94)</td></tr>
<tr><td>Intercept</td><td>1.75*** (0.34)</td></tr>
<tr><td>Robust R^2_w</td><td>0.77</td></tr>
</table>
No. of observations: 346. Standard errors are reported in parentheses. *p<0.05, **p<0.01, ***p <0.001. OHS: occupational health and safety. PSR: psychosocial risks. NI: non-inspected. I: inspected. Reference value for interaction terms: NI/tertiary/<100.

Improvement of working conditions
<table>
<tr><td></td><td>Scores 2nd survey with standard error</td></tr>
<tr><td>Score 1rst survey</td><td>0.51*** (0.05)</td></tr>
<tr><td>Interaction terms</td><td></td></tr>
<tr><td>NI/tertiary/≥100</td><td>0.13 (0.46)</td></tr>
<tr><td>NI/secondary/<100</td><td>-1.10 (0.62)</td></tr>
<tr><td>NI/secondary/≥100</td><td>-0.05 (0.66)</td></tr>
<tr><td>I/tertiary/<100</td><td>0.18 (0.41)</td></tr>
<tr><td>I/tertiary/≥100</td><td>0.70 (0.39)</td></tr>
<tr><td>I/secondary/<100</td><td>0.60 (0.71)</td></tr>
<tr><td>I/secondary/≥100</td><td>0.70 (0.83)</td></tr>
<tr><td>Intercept</td><td>3.84*** (0.52)</td></tr>
<tr><td>Robust R^2_w</td><td>0.37</td></tr>
</table>
No. of observations: 346. Standard errors are reported in parentheses. *p<0.05, **p<0.01, ***p <0.001. OHS: occupational health and safety. PSR: psychosocial risks. NI: non-inspected. I: inspected. Reference value for interaction terms: NI/tertiary/<100.
</td>
<td></td>
</tr>
</table>

<table>
<tr><th>General</th><th>Study</th><th>Population</th><th>Exposure</th><th>Outcome</th><th>Results</th><th>Comments</th></tr>
<tr><td></td><td></td><td></td><td></td><td></td><td>
Ability in PSR prevention
<table>
<tr><th></th><th>Scores 2nd survey with standard error</th></tr>
<tr><td>Score 1rst survey</td><td>0.64*** (0.05)</td></tr>
<tr><td>Interaction terms</td><td></td></tr>
<tr><td>NI/tertiary/≥100</td><td>1.22** (0.40)</td></tr>
<tr><td>NI/secondary/<100</td><td>-0.36 (0.54)</td></tr>
<tr><td>NI/secondary/≥100</td><td>0.38 (1.29)</td></tr>
<tr><td>I/tertiary/<100</td><td>1.53*** (0.42)</td></tr>
<tr><td>I/tertiary/≥100</td><td>2.28*** (0.40)</td></tr>
<tr><td>I/secondary/<100</td><td>1.35* (0.67)</td></tr>
<tr><td>I/secondary/≥100</td><td>1.70* (0.72)</td></tr>
<tr><td>Intercept</td><td>1.33*** (0.30)</td></tr>
<tr><td>Robust R^2_w</td><td>0.65</td></tr>
</table>
No. of observations: 346. Standard errors are reported in parentheses. *p<0.05, **p<0.01, ***p <0.001. OHS: occupational health and safety. PSR: psychosocial risks. NI: non-inspected. I: inspected. Reference value for interaction terms: NI/tertiary/<100.

Results of multiple regression analysis for single items of scales (no data shown): inspection statistically significant effects on: charter or rules on PSRs[1], role clarity for PSR management[1] (p < .001); changes to the contractual requirements of some employees to reduce stress[2] (p < .01); confidential counselling[1], conflict resolution procedure[1], and recording of working hours[3] (p < .05) (scales: 1=specific PSR management scale, 2=improvement of working condition scale, 3=not included in a scale)

Short summary of findings:
- Inspections particularly improved OHS management practices and perceived workplace ability
- Inspected large companies from tertiary sector improved on almost every scale; strongest increases on specific PSR management and perceived ability for prevention.
- Improvements also in some non-inspected categories -> especially on OHS management practices in large companies from secondary sector, and on the perceived ability for large tertiary firms.
- Increase of willingness to prevent PSR only in inspected companies.
- Almost no effect of inspection on worker participation and general improvement of working conditions.
</td><td></td></tr>
<tr><td>First Author, Year</td><td>Study name: -</td><td>Recruitment method/ Data source:</td><td>Description of intervention:</td><td>Outcome name: Number of work accidents</td><td>Descriptive Statistics:
Tab.1: Descriptive statistics</td><td>Funding: Spanish ministry of economy, industry and</td></tr>
</table>

<table>
<tr><th>General</th><th>Study</th><th>Population</th><th>Exposure</th><th>Outcome</th><th>Results</th><th>Comments</th></tr>
<tr>
<td>Lafuente 2021
#2690

Extracted by: MS

Checked by: UBA</td>
<td>Country: Spain

Study design: ecological study (annual reports)

Time of Study: 2003-2012</td>
<td>Data from 50 Spanish provinces (NUTS-3 level), i.e. 500 province-year observations;
-data for number of work accidents from General Office of Statistics of the Spanish Ministry of Employment and Social Security
-data on OSH interventions at province level from the Annual Report of Occupational Health and Safety from Spanish Ministry of Employment and Social Security
Workers aged 18-65 years

Matching criteria: NA

invited companies: NA

companies at baseline: NA

Response: NA

companies at follow –up: NA

Loss-to-follow-up: NA

Work characteristics: no restriction</td>
<td>Inspections (Number), Economic sanctions

Study groups: NA

Time of measurement: 2003-2012</td>
<td>Outcome definition and assessment: Number of work accidents reported by organisations

Time of measurement: 2003-2012</td>
<td>
<table>
<tr><th>Year</th><th>Safety inspections (thousands)</th><th>Safety sanctions s (millions of 2011 euro)</th><th>Work accidents (thousand)</th></tr>
<tr><td>2003</td><td>9.24</td><td>1.79</td><td>18.97</td></tr>
<tr><td>2004</td><td>9.38</td><td>1.74</td><td>19.06</td></tr>
<tr><td>2005</td><td>9.75</td><td>2.01</td><td>19.53</td></tr>
<tr><td>2006</td><td>9.72</td><td>2.04</td><td>20.26</td></tr>
<tr><td>2007</td><td>8.56</td><td>2.28</td><td>19.97</td></tr>
<tr><td>2008</td><td>7.16</td><td>2.19</td><td>17.32</td></tr>
<tr><td>2009</td><td>7.19</td><td>1.50</td><td>13.90</td></tr>
<tr><td>2010</td><td>7.59</td><td>1.33</td><td>12.89</td></tr>
<tr><td>2011</td><td>7.33</td><td>1.20</td><td>11.60</td></tr>
<tr><td>2012</td><td>7.46</td><td>1.08</td><td>9.40</td></tr>
<tr><td>Total</td><td>8.34</td><td>1.72</td><td>16.29</td></tr>
</table>

Tab.3: Descriptive statistics: Total efficiency score
<table>
<tr><th></th><th>Safety inspections (thousand)</th><th>Economic sanctions (millions of 2011 euro)</th></tr>
<tr><td>Period 2003–2007</td><td>9.33</td><td>1.97</td></tr>
<tr><td>Period 2008–2012</td><td>7.35</td><td>1.46</td></tr>
<tr><td>Variation rate</td><td>-21.3%</td><td>-25.8%</td></tr>
<tr><td>Minimum</td><td>-58.3%</td><td>-53.4%</td></tr>
<tr><td>Maximum</td><td>26.4%</td><td>38.4%</td></tr>
</table>

Statistical methods used: directional distance function (DDF) equation model proposed with: input vector (x): (x1) number of workers aged between 18 and 65 years old, (x2) number of businesses, (x3) number of safety inspections (control or monitoring mechanism for reducing work accidents) and (x4) safety sanctions imposed to businesses expressed in millions of constant 2011 euro
- group A: provinces that abandoned the persistence area during economic crisis period: Burgos (Castile Leon), Jaen (Andalusia), Seville (Andalusia), Coruna (Galicia), Lugo (Galicia), Ourense (Galicia), Ciudad Real (Castile La Mancha), and Guadalajara (Castile La Mancha)
- group B: Communities of Aragon (Huesca, Teruel and Zaragoza), Asturias, Basque Country (´Alava and Guipúzcoa), Cantabria, Castile Leon (Salamanca, Segovia, Soria and Zamora), Catalonia (Girona, Lleida and Tarragona), Madrid, Navarra, and Valencia (Alicante and Castellon)
</td>
<td>competitiveness (grant: ECO2017-86305-C4-2-R)

Conflict of Interest stated: no

Other comments:
- also includes GDP (production of desirable) as outcome</td>
</tr>
</table>

General	Study	Population	Exposure	Outcome	Results	Comments
					- group C: Communities of Andalusia (Almeria and Huelva), Canary Islands (Las Palmas and Sta. Cruz Tenerife), and Castile La Mancha (Toledo)<br><br>**Unadjusted/ adjusted estimates with precision (e.g. 95% confidence interval) for each outcome:**<br>- keeping input levels fixed -> economic (labour and business stock) and OHS (inspections and economic sanctions) inputs -> simultaneously expand GDP and lower their work accidents by 8.54%<br>- higher inefficiency during low economic growth between 2008-2012 than in time period of economic growth (10.3% versus 8.06%, Wilcoxon-signed rank test: Z=3.242, p=0.0012)<br>- Group A: major decline in efficiency level between growth period (average inefficiency level=5.3%) and period of economic slowdown (average inefficiency level=14.7%); below-average reduction inspections and sanctions between growth and crisis period (variation in inspections = -4.6%, variation in sanctions =- 9.5%), compared to the values reported for the rest of Spain (variation in inspections = -23.4%, variation in safety economic sanctions = -27.3%) BUT reduction in rate of work accidents in group A (28.33%) is ten percentage points below average reduction observed in the rest of Spanish provinces (38.29%)<br>- Group B: inefficiency level below 10%; inefficiency level remained mostly unchanged between the growth (average inefficiency = 2.32%) and slowdown (average inefficiency = 2.37%) period<br>- Group C: inefficient provinces in both, the growth (average inefficiency = 20.77%) and the economic slowdown (average inefficiency = 21.86%) periods<br>- Group A: fall in the proportion of construction establishments (-3.08%) between 2008 and 2012 is lower than the fall in safety interventions (variation in inspections = - 4.62%, variation in economic sanctions = -9.52%) + lower reduction in proportion of construction establishments than in the group of consistently efficient (Group B) and consistently inefficient (Group C) provinces (-5.31% and -6.35%, respectively)<br>- at the province level results indicate that the changes in OHS interventions introduced by Spanish policy makers in 2008 had a heterogeneous impact on territorial efficiency: Efficiency declines	

<table>
<tr><th>General</th><th>Study</th><th>Population</th><th>Exposure</th><th>Outcome</th><th>Results</th><th>Comments</th></tr>
<tr><td></td><td></td><td></td><td></td><td></td><td>were not more pronounced in provinces that introduced drastic cuts in OHS policy controls (i.e., safety inspections and sanctions for safety violations).

Short summary of findings:
- provinces with strong decrease in OHS controls (safety controls and sanctions) have higher inefficiency levels, but heterogenous effect on efficiency levels
- “Results suggest that effective OHS policy is not implicitly linked to the mere deployment of more OHS policy controls”</td><td></td></tr>
<tr><td>**First Author, Year**
Lafuente, 2020
#2922

Extracted by: MS

Checked by:</td><td>**Study name:** -

Country: 24 European Countries: Austria, Bulgaria, Croatia, Cyprus, Czech Republic, Estonia, Finland, France, Germany, Hungary, Ireland, Latvia, Lithuania, Malta, Norway, Poland,</td><td>**Recruitment method/ Data source:**
- data on number of work accidents from EUROSTAT databases
- data on number of work inspections from International Labour Organization (ILO)

Matching criteria: NA

 # invited: NA

 # baseline:
N=192 observations from (24 countries); 1 observation per country per year

Response: NA

 # follow –up: NA

Loss-to-follow-up: NA

Work characteristics: no restriction</td><td>**Description of intervention:** Rate of labour inspections per worker

Labour inspections = visits (physical presence) of a labour inspector in workplace for carrying out work inspections documented as required by national legislation

Study groups: NA</td><td>**Outcome name:** Rate of work accidents

Outcome definition and assessment:
Rate of work accidents = total number of accidents at work (including injuries and fatal accidents) divided by total number of workers in the economy
-> number of work accidents: is the sum of all accidents during course of work with >3 calendar days of absence from work and fatalities (accidents that lead to death of employee)</td><td>**Descriptive Statistics:**
Tab. 1: Descriptive statistics (2008-2015)
<table>
<tr><td colspan="2">Number of work accidents (thousands)</td></tr>
<tr><td>N</td><td>192</td></tr>
<tr><td>Mean</td><td>94.5724</td></tr>
<tr><td>Standard Deviation</td><td>179.5099</td></tr>
<tr><td>Minimum</td><td>0.9000</td></tr>
<tr><td>Maximum</td><td>782.2500</td></tr>
<tr><td colspan="2">Work accident rate (accidents per worker)</td></tr>
<tr><td>N</td><td>192</td></tr>
<tr><td>Mean</td><td>0.0083</td></tr>
<tr><td>Standard Deviation</td><td>0.0064</td></tr>
<tr><td>Minimum</td><td>0.0003</td></tr>
<tr><td>Maximum</td><td>0.0263</td></tr>
<tr><td colspan="2">Work inspections per worker</td></tr>
<tr><td>N</td><td>192</td></tr>
<tr><td>Mean</td><td>0.0102</td></tr>
<tr><td>Standard Deviation</td><td>0.0063</td></tr>
<tr><td>Minimum</td><td>0.0005</td></tr>
<tr><td>Maximum</td><td>0.0359</td></tr>
</table>

Statistical methods used: Fixed-effects regression model with robust standard errors to correct for autocorrelation of errors to constant country-specific effects
Model 1 includes GDP per worker, the capital-to-labour ratio, and the rate of work inspections per worker as independent Variables. Model 2 + human capital

Increase according to equation:
In Rate of work accidents$_{it}$ = $A + \alpha$ln GDP per worker$_{it}$ + βln Capital-to-labour ratio$_{it}$ + δln Inspections per worker$_{it}$ + ψln Human capital ratio$_{it}$ + $\lambda T_t + \pi_i + \varepsilon_{it}$</td><td>**Funding:** NR

Conflict of Interest stated: no

Other comments:
- study investigated the effect of macroeconomic variables on work accidents</td></tr>
</table>

<table>
<tr><th>General</th><th>Study</th><th>Population</th><th>Exposure</th><th>Outcome</th><th>Results</th><th>Comments</th></tr>
<tr><td></td><td>Portugal, Romania, Slovak Republic, Slovenia, Spain, Sweden, Switzerland, and the United Kingdom

Study design: ecological study

Time of Study: 2008-2015</td><td></td><td>Time of measurement: 2008-2015</td><td>Time of measurement: 2008-2015</td><td>(T–1) = dummy variables ruling out potential time effects linked to unobserved changes in economic and environmental conditions common to all countries(i) + error term: a time-invariant effect controlling for country-specific unobserved heterogeneity that is uncorrelated with parameters (π), and a normally distributed error (ε) that varies cross-countries and cross-time (t)

Unadjusted/ adjusted estimates with precision (e.g. 95% confidence interval) for each outcome:
Tab 2: Association between inspections per worker and rate of work accidents:
Model 1: estimate = -0.1903** (0.0797 robust standard error)
Model 2: estimate = -0.1774** (0.0856 robust standard error)

Short summary of findings:
A 10% increase (decrease) in the rate of inspections per worker is associated with a fall (rise) of 1.68% in the rate of work accidents.</td><td></td></tr>
<tr><td>First Author, Year
Tatsaki, 2019
#2819

Extracted by: MS

Checked by:</td><td>Study name: -

Country: Greece

Study design: ecological study (annual reports)

Time of Study:</td><td>Recruitment method/ Data source:
Data of work accidents and sanctions from official website of Greek Ministry of Labour, Social Insurance and Social Solidarity. Annual reports of Greek Labour Inspectorate are posted there.

Matching criteria: NA

invited companies: NA

companies at baseline: NA</td><td>Description of intervention: Work accidents (includes fatal and non-fatal)

Study groups: NA

Time of measurement:</td><td>Outcome name: Sanctions (including fines, temporary closing down and lawsuits)

Outcome definition and assessment: Sanctions by authority

Time of measurement: Annually 2000-2013</td><td>Descriptive Statistics:
Fig. 1: Reported work accidents and sanctions
<table>
<tr><th></th><th># of reported sanctions</th><th># of reported work accidents</th></tr>
<tr><td>2000</td><td>899</td><td>4.032</td></tr>
<tr><td>2001</td><td>2.247</td><td>5.155</td></tr>
<tr><td>2002</td><td>5.260</td><td>6.021</td></tr>
<tr><td>2003</td><td>6.299</td><td>6.329</td></tr>
<tr><td>2004</td><td>5.719</td><td>6.333</td></tr>
<tr><td>2005</td><td>4.459</td><td>6.043</td></tr>
<tr><td>2006</td><td>4.930</td><td>6.255</td></tr>
<tr><td>2007</td><td>4.648</td><td>6.561</td></tr>
<tr><td>2008</td><td>4.047</td><td>6.657</td></tr>
<tr><td>2009</td><td>3.588</td><td>6.381</td></tr>
<tr><td>2010</td><td>3.219</td><td>5.721</td></tr>
<tr><td>2011</td><td>2.171</td><td>5.203</td></tr>
<tr><td>2012</td><td>1.892</td><td>4.858</td></tr>
<tr><td>2013</td><td>1.825</td><td>5.126</td></tr>
</table></td><td>Funding: NR

Conflict of Interest stated: no

Other comments:
- analysis the relationship between work accidents, sanctions and gross domestic product (GDP)
- special monitoring program of the Athens Olympic</td></tr>
</table>

<table>
<tr><th>General</th><th>Study</th><th>Population</th><th>Exposure</th><th>Outcome</th><th>Results</th><th>Comments</th></tr>
<tr>
<td></td>
<td>2000-2013</td>
<td>Response: NA

companies at follow –up: NA

Loss-to-follow-up: NA

Work characteristics: no restriction</td>
<td>Annually 2000-2013</td>
<td></td>
<td>Statistical methods used: Linear regression, dependent variable: sanctions
Sanctions =−5934.345+2.616 * Occupational accidents−2.776 * 10^{-8} * GDP

Unadjusted/ adjusted estimates with precision (e.g. 95% confidence interval) for each outcome:

Tab: 4 Coefficients for work accidents
<table>
<tr><td>Unstandardized Coefficients B</td><td>2.616</td></tr>
<tr><td>Std. Error</td><td>0.289</td></tr>
<tr><td>Standardized Coefficients Beta</td><td>1.220</td></tr>
<tr><td>t</td><td>9.049</td></tr>
<tr><td>Sign.</td><td><0.001</td></tr>
</table>

Short summary of findings:
“every case of occupational accident increases sanctions by 2.616”</td>
<td>Games’ projects during 2000-2003
- dependent variable in model is sanctions</td>
</tr>
<tr>
<td>First Author, Year
Beck, 2019
#421

Extracted by: MS

Checked by: UBA</td>
<td>Study name: -

Country: Germany

Study design: Cross-sectional

Time of Study: June, 8th 2015 – August, 18th 2015 (from</td>
<td>Recruitment method/ Data source:
Data from evaluation of the German Joint Occupational Safety and Health Strategy (Gemeinsame Deutsche Arbeitsschutzstrategie – GDA); stratified random sample of 6.500 companies with min of 1 employee in mid-2015
(Sleik et al. 2015: sample population divided into 4 employee size classes (1-9, 10-49, 50-249, ≥250) and according to 16 federal states)

Matching criteria: NA</td>
<td>Description of intervention:
Question if company had been visited by a competent OSH authority or accident insurance organisation since January 2013 and whether psychosocial risk assessment</td>
<td>Outcome name:
Workplace risk assessment (WRA): inclusion of psychological factors

Outcome definition and assessment:
-questionnaire survey (computer-assisted telephone interview – CATI) of highest-ranked company member with responsibilities in OSH coordination;</td>
<td>Descriptive Statistics:
Interviewed persons: 46% company owners or managing directors, 23% safety engineers and 31% company members (mostly managers) with OSH coordination responsibilities

Tab. 1: Overview sample (unw = unweighted, w = weighted)
<table>
<tr><th></th><th>N (unw)</th><th>% (w) (95 %CI)</th></tr>
<tr><td colspan="3">Inspection by OSH authority concerning psychosocial risk assessment (n=5.803)</td></tr>
<tr><td>No</td><td>4.348</td><td>88 (87-90)</td></tr>
<tr><td>Yes</td><td>1.455</td><td>12 (10-13)</td></tr>
<tr><td colspan="3">WRA carried out (6.355)</td></tr>
<tr><td>No</td><td>1.439</td><td>46 (44-49)</td></tr>
<tr><td>Yes</td><td>4.916</td><td>54 (51-57)</td></tr>
<tr><td colspan="3">Results of WRA documented (n = 4.878)</td></tr>
<tr><td>No</td><td>256</td><td>14 (12-17)</td></tr>
<tr><td>Party</td><td>96</td><td>4 (3-6)</td></tr>
<tr><td>Yes</td><td>4.526</td><td>82 (79-84)</td></tr>
<tr><td colspan="3">Needs for improvement identified (n = 4.729)</td></tr>
<tr><td>No</td><td>1.730</td><td>52 (49-56)</td></tr>
<tr><td>Yes</td><td>2.999</td><td>48 (44-51)</td></tr>
<tr><td colspan="3">Measures taken (n = 2.991)</td></tr>
</table></td>
<td>Funding:
Gemeinsame Deutsche Arbeitsschutz-strategie – GDA

Conflict of Interest stated: yes

Other comments:
- very low response (15%)</td>
</tr>
</table>

<table>
<tr><th>General</th><th>Study</th><th>Population</th><th>Exposure</th><th>Outcome</th><th>Results</th><th>Comments</th></tr>
<tr>
<td></td>
<td>Sleik et al. 2015)</td>
<td>

# invited companies: n = 42.279 (from Sleik et al. 2015)

# companies at baseline: n=6500

Response: 15.4% (from Sleik et al. 2015)

# companies at follow –up: NA

Loss-to-follow-up: NA

Work characteristics:
Tab. 1: Overview sample (unw=unweighted, w=weighted)

<table>
<tr><th></th><th>N (unw)</th><th>% (w) (95 %CI)</th></tr>
<tr><td colspan="3">Number of employees (n=6.500)</td></tr>
<tr><td>1-9</td><td>1.690</td><td>69 (67-71)</td></tr>
<tr><td>10-49</td><td>1.891</td><td>25 (24-27)</td></tr>
<tr><td>50-249</td><td>1.838</td><td>5 (4-5)</td></tr>
<tr><td>≥250</td><td>1.081</td><td>1 (1-1)</td></tr>
<tr><td colspan="3">Sector I (n=6.500)</td></tr>
<tr><td>Production/ agriculture</td><td>2.001</td><td>23 (21-25)</td></tr>
<tr><td>Services</td><td>4.499</td><td>77 (75-79)</td></tr>
<tr><td colspan="3">Sector II (n=6.465)</td></tr>
<tr><td>Private</td><td>5.198</td><td>92 (90-.93)</td></tr>
<tr><td>Public</td><td>1.267</td><td>8 (7-10)</td></tr>
</table>

*Sleik K, Baier E, Künzel S et al (2015): GDA Dachevaluation Betriebs- und Beschäftigten-befragung 2015. TNS Infratest Sozialforschung.

</td>
<td>

was subject of visit (yes; no; do not know; n/a)

Study groups: reference group = no inspection since January 2013

Time of measurement: From January 2013 to June – August 2015

</td>
<td>

all information on the WRA is based on the responses from the companies surveyed. The study authors did not have access to the WRA and did not verify the validity of the companies' responses
- basic question: Are risk assessments being carried out at the workplaces in your company (yes; no; do not know; no answer (n/a))?"
-Pattern A—Inactive with regard to WRA: Carrying out WRAs not confirmed (response 'no', 'do not know' or 'n/a')
-Pattern B—WRA without consideration of psychosocial Factors: Carrying out WRAs confirmed (response 'yes'), but no

</td>
<td>

<table>
<tr><td>No</td><td>43</td><td>1 (1-2)</td></tr>
<tr><td>Not yet but scheduled</td><td>133</td><td>4 (2-5)</td></tr>
<tr><td>Yes</td><td>2.815</td><td>95 (93-97)</td></tr>
<tr><td colspan="3">Measures checked for effectiveness (n = 2.783)</td></tr>
<tr><td>No</td><td>391</td><td>23 (19-27)</td></tr>
<tr><td>Not yet but scheduled</td><td>526</td><td>20 (17-24)</td></tr>
<tr><td>Partly</td><td>32</td><td>0 (0-1)</td></tr>
<tr><td>Yes</td><td>1.834</td><td>57 (52-61)</td></tr>
</table>

Tab.3: WRA implementation patterns by company characteristics: Inspections by OSH authority concerning psychological risk assessment (n = 5.803)

<table>
<tr><th></th><th colspan="4">Inspections by OSH authority concerning psychological risk assessment</th></tr>
<tr><td></td><td colspan="2">No</td><td colspan="2">Yes</td></tr>
<tr><td></td><td>N (unw)</td><td>% (w) (95 %CI)</td><td>N (unw)</td><td>% (w) (95 %CI)</td></tr>
<tr><td>Pattern A</td><td>1304</td><td>52 (49-55)</td><td>78</td><td>20 (14-28)</td></tr>
<tr><td>Pattern B</td><td>1609</td><td>30 (28-33)</td><td>328</td><td>30 (24-37)</td></tr>
<tr><td>Pattern C</td><td>1435</td><td>18 (16-20)</td><td>1049</td><td>50 (34-56)</td></tr>
</table>

Statistical methods used: Multinomial logistic regression with unweighted data; odds ratios (OR) indicate the chance that analysis restricted to companies ≥5 employees

Unadjusted/ adjusted estimates with precision (e.g. 95% confidence interval) for each outcome:

Tab.5: Results of multinomial regression: only results extracted for: Inspection by OSH authority concerning psychosocial risk assessment

<table>
<tr><th></th><th colspan="2">WRA Pattern B</th><th colspan="2">WRA Pattern C</th></tr>
<tr><td></td><td>OR</td><td>95% CI</td><td>OR</td><td>95% CI</td></tr>
<tr><td>No</td><td>1</td><td></td><td>1</td><td></td></tr>
<tr><td>Yes</td><td>1.2</td><td>0.8-1.6</td><td>3.4</td><td>2.4-4.7</td></tr>
</table>

N = 4271 companies with ≥5 employees
(other variables in model: number of employees, sector, economic situation, magnitude of psychological risks, work council, safety specialist assistance, occupational health assistance, level of knowledge about legal requirements in OSH, OSH training of managers, attitude towards OSH benefits)

</td>
<td></td>
</tr>
</table>

General	Study	Population	Exposure	Outcome	Results	Comments
				consideration of hazards related to psychosocial factors (response ‘no’, ‘not applicable’, ‘do not know’ or ‘n/a’).<br>-*Pattern C—WRA considering psychosocial factors:* Both implementation of WRAs and consideration of psychosocial factors were confirmed (response category ‘yes’).<br>-*(Differentiation of Pattern C in C.*1 & C2)<br><br>**Time of measurement:**<br>June – August 2015	**Short summary of findings:**<br>“After company size, the availability of safety specialist assistance, the availability of occupational health specialist assistance and having had an inspection visit by OSH authorities<br>show the strongest effects on psychosocial risk assessment occurrence (= WRA pattern C), with ORs larger than 3.”	
**First Author, Year**<br>Finnager Garshol, 2024<br>#3548<br><br>Indregard et al. 2019	**Study name:**<br>Effects of the Labour Inspection Authority’s Regulatory Tools	**Recruitment method/ Data source:**<br>- In January 2019, 422 municipalities with home-care services with 3->4.000 employees -> eligible municipalities: >20-<100 care workers<br>- Exclusion of municipalities with recent labour	**Description of intervention:**<br><u>Labour inspections:</u><br>Standardized inspection routines of Norwegian Labour Inspection	**Outcome name:**<br>Physician-certified sick leave: total number of sick leave, number of sick leave periods<br><br>General Health<br>Mental distress<br>Musculoskeletal complaints	**Descriptive Statistics:**<br>- no significant group differences on age, gender, marital status, educational background, type of employment or leadership but difference in mean percentage of full-time equivalent (FTE) employment between guidance (lower) than control group.<br>Of 1667 participants, 467 did not gave consent for collection of registry data.<br>-> not giving consent: younger, less education, and less FTE employment<br><br>Tab.1: Characteristics<br><table><tr><td></td><td>Inspection</td><td>Guidance</td><td>Control</td></tr></table>	**Funding:**<br>STAMI and NLIA<br><br>**Conflict of Interest stated:** yes<br><br>**Other comments:**<br>- Clinicial Trials ID: NCT0355163<br>- Regional Committees for

<table>
<tr><th>General</th><th>Study</th><th>Population</th><th>Exposure</th><th>Outcome</th><th>Results</th><th>Comments</th></tr>
<tr>
<td>#389 (study protocol)

Extracted by: MS

Checked by:</td>
<td>on Work Environment and Health in the Norwegian Home-care Services project (EAVH)

Country: Norway

Study design: Cluster-randomized controlled trial with 2 follow-ups

Time of Study: January 2019 – June 2021</td>
<td>inspections (i.e. in 2017-2018)
- N=187 eligible municipalities
-> thereof 132 randomly assigned to 1 of 4 study groups
- Randomization according to random numbers assigned to each municipality -> assigning the first 33 to one group, the next 33 to another group, etc.
- Invitation of municipalities via letters and e-mails
- n=1.202 consented to collection of registry data: control: n=478, inspection: n=368 and guidance: n=356

Matching criteria: NA

# invited companies:
n=132 municipalities
<u>control:</u> n=33 -> acceptance of study invitation: n=29
<u>inspection:</u> n=33 -> acceptance of study invitation: n=28
<u>guidance:</u> n=33 -> acceptance of study invitation: n=24
<u>online risk assessment:</u> n=33 -> acceptance of study invitation: n=23
-> online risk assessment group relocated to other study groups (control: n=6,</td>
<td>Authority (NLIA): participating home-care services notice and information 3 weeks prior to inspection. Inspections carried out by trained inspectors. Standardized checklist operationalizing relevant legislation (the Internal Control Regulation and the Working Environment Act) used during inspections. Checking compliance with legislation & focused on exposures related to psychosocial, organizational, and</td>
<td>Outcome definition and assessment:
<u>Web-based questionnaire</u> (developed by National Institute of Occupational Health (STAMI) (paper-based version on request)
General health: “How would you rate your health in general? 0=very bad, 1=bad, 2=moderate, 3=good, and 4=yery good
Mental distress: Hopkins Symptom Checklist (HSCL-5) (5 items, 1=not at all to 4=extremely); symptoms previous week
Musculoskeletal complaints: 6 items adapted from Steingrimsdottir et al. 2004; symptoms refer to last 4 weeks: headache; neck</td>
<td>
<table>
<tr><th></th><th>N(%) or mean (SD)</th><th>N(%) or mean (SD)</th><th>N(%) or mean (SD)</th></tr>
<tr><td colspan="4">Gender</td></tr>
<tr><td>Male</td><td>22 (4.3)</td><td>22 (4.6)</td><td>28 (4.2)</td></tr>
<tr><td>Female</td><td>495 (95.7)</td><td>457 (95.4)</td><td>645 (95.8)</td></tr>
<tr><td>Age</td><td>46.1 (11.6)</td><td>44.7 (12.2)</td><td>45.3 (12.1)</td></tr>
<tr><td colspan="4">Employment</td></tr>
<tr><td>Permanent</td><td>482 (94.1)</td><td>434 (92.7)</td><td>619 (93.5)</td></tr>
<tr><td>Temporary</td><td>16 (3.1)</td><td>16 (3.4)</td><td>18 (2.7)</td></tr>
<tr><td>Substitute/ on-call</td><td>11 (2.2)</td><td>16 (3.4)</td><td>23 (3.5)</td></tr>
<tr><td>Other</td><td>3 (0.6)</td><td>2 (0.4)</td><td>2 (0.3)</td></tr>
<tr><td>Percentage FTE</td><td>79.0 (22.1)</td><td>78.3 (22.6)</td><td>81.2 (21.5)</td></tr>
<tr><td colspan="4">Leader responsibilities</td></tr>
<tr><td>Top tier leader</td><td>89 (17.7)</td><td>92 (19.7)</td><td>106 (16.3)</td></tr>
<tr><td>Middle tier leader</td><td>44 (8.8)</td><td>37 (7.9)</td><td>48 (7.4)</td></tr>
<tr><td>No leadership responsi.</td><td>370 (73.6)</td><td>337 (72.3)</td><td>496 (76.3)</td></tr>
<tr><td>Consent for sick leave data</td><td>368 (71.2)</td><td>356 (74.3)</td><td>478 (71.0)</td></tr>
</table>

Tab.2: Sick leave data
<table>
<tr><th></th><th>Inspection</th><th>Guidance</th><th>Control</th></tr>
<tr><td></td><td>N(%) or Δ % or mean (SD)</td><td>N(%) or Δ % or mean (SD)</td><td>N(%) or Δ % or mean (SD)</td></tr>
<tr><td colspan="4">IDs ≥1 physician certified sick leave periods pre-intervention</td></tr>
<tr><td>Musculoskeletal & psychological</td><td>67 (18.2)</td><td>75 (21.1)</td><td>132 (27.6)</td></tr>
<tr><td>Musculoskeletal</td><td>46 (12.5)</td><td>60 (16.9)</td><td>99 (20.7)</td></tr>
<tr><td>Psychological</td><td>28 (7.6)</td><td>20 (5.6)</td><td>41 (8.6)</td></tr>
<tr><td colspan="4">IDs ≥1 physician certified sick leave periods post-intervention</td></tr>
<tr><td>Musculoskeletal & psychological</td><td>94 (25.5)</td><td>109 (30.6)</td><td>154 (32.2)</td></tr>
<tr><td>Musculoskeletal</td><td>72 (19.6)</td><td>82 (23.0)</td><td>118 (24.7)</td></tr>
<tr><td>Psychological</td><td>33 (9.0)</td><td>36 (10.1)</td><td>46 (9.6)</td></tr>
<tr><td>Change in %-points pre- to post-inter-vention musculoskeletal & psychological</td><td>7.3</td><td>9.6</td><td>4.6</td></tr>
</table>
</td>
<td>Medical and Health Research Ethics (REC Southeast) (2018/2003/ REK Sør-Øst C)
data handling and storage approved by the Norwegian Centre for Data Research (566128)
- drop-outs were younger, had less education, lower percentage of full-time equivalent employment and were more often listed as “other care staff”</td>
</tr>
</table>

<table>
<tr><th>General</th><th>Study</th><th>Population</th><th>Exposure</th><th>Outcome</th><th>Results</th><th>Comments</th></tr>
<tr>
<td></td>
<td></td>
<td>inspection: n=6, guidance: n=11)

# companies at baseline:
n=96 municipalities with n=3.985 employees of which 1.669 participated at baseline
control: n=673 employees from 35 municipalities
inspection: n=517 employees from 30 municipalities
guidance: n=479 employees from 31 municipalities

Response:
Municipalities: 73%
Employees: 42%

companies at follow –up:
of employees
6-month follow-up:
control: n=363
inspection: n=285
guidance: n=269

12-months follow-up:
control: n=220
inspection: n=185
guidance: n=172

Physician-certified sick leave:
control: n=478
inspection: n=368
guidance: n=356

Loss-to-follow-up:
Drop-out rate of employees: 65%</td>
<td>mechanical work
Guidance: 5-7 home-care services assigned to one-time workshop based on their geographic location. Workshops lead by 2 trained labour inspectors from the NLIA led. Invitation of manager, safety representative, and employee representative. Information topic “work environment and employee health”. Participants asked to prepare presentations of specific</td>
<td>pain; back pain; pain in the should or upper arm; pain in the lower arm, wrist, or hands; pain in the hips, legs, knees, or feet (1=not troubled, 2=a little troubled, 3=intensely troubled and 4=very troubled); general pain intensity in the preceding week (11-point rating scale, 0=no pain, 10=worst possible pain)

Sick-leave data:
From Norwegian Labour and Welfare Administration: Diagnoses based on International Classification of Primary Care 2 (ICPC-2): all musculoskeletal and psychological diagnoses (L and P-codes), all musculoskeletal diagnoses (L-codes), and all</td>
<td>
<table>
<tr><td colspan="4">Physician-certified sick leave periods pre-intervention</td></tr>
<tr><td>Musculoskeletal & psychological</td><td>0.27 (0.7)</td><td>0.29 (0.6)</td><td>0.38 (0.7)</td></tr>
<tr><td>Musculoskeletal</td><td>0.18 (0.6)</td><td>0.21 (0.5)</td><td>0.28 (0.7)</td></tr>
<tr><td>Psychological</td><td>0.09 (0.4)</td><td>0.07 (0.3)</td><td>0.10 (0.3)</td></tr>
<tr><td colspan="4">Physician-certified sick leave periods post-intervention</td></tr>
<tr><td>Musculoskeletal & psychological</td><td>0.45 (1.0)</td><td>0.48 (0.9)</td><td>0.47 (0.8)</td></tr>
<tr><td>Musculoskeletal</td><td>0.31 (0.7)</td><td>0.33 (0.7)</td><td>0.35 (0.7)</td></tr>
<tr><td>Psychological</td><td>0.14 (0.6)</td><td>0.14 (0.5)</td><td>0.11 (0.4)</td></tr>
<tr><td colspan="4">Physician-certified sick leave days pre-intervention</td></tr>
<tr><td>Musculoskeletal & psychological</td><td>11.90 (43.6)</td><td>16.08 (64.6)</td><td>26.22 (74.0)</td></tr>
<tr><td>Musculoskeletal</td><td>9.05 (41.7)</td><td>12.25 (55.4)</td><td>19.82 (66.5)</td></tr>
<tr><td>Psychological</td><td>2.86 (13.8)</td><td>4.82 (34.5)</td><td>6.39 (35.3)</td></tr>
<tr><td colspan="4">Physician-certified sick leave days post-intervention</td></tr>
<tr><td>Musculoskeletal & psychological</td><td>30.75 (82.1)</td><td>32.11 (84.1)</td><td>34.52 (86.2)</td></tr>
<tr><td>Musculoskeletal</td><td>20.58 (67.1)</td><td>21.84 (68.6)</td><td>23.57 (75.2)</td></tr>
<tr><td>Psychological</td><td>10.17 (50.6)</td><td>10.27 (51.2)</td><td>10.94 (46.8)</td></tr>
</table>

Statistical methods used: Intent-to-treat analysis

Linear mixed models: independent variables: time, time x group and employment percentage
(time=waves data collection, full-time equivalent included because of differences between guidance and control group)

Mixed-binomial regression for analysis of physician-certified sick leave: municipalities included as random effects
-> all analysis adjusted for baseline outcome variables

Unadjusted/ adjusted estimates with precision (e.g. 95% confidence interval) for each outcome:

Tab.3: Mean (SD) at baseline
<table>
<tr><th></th><th>Inspection Mean (SD)</th><th>Guidance Mean (SD)</th><th>Control Mean (SD)</th></tr>
</table>
</td>
<td></td>
</tr>
</table>

<table>
<tr><th>General</th><th>Study</th><th>Population</th><th>Exposure</th><th>Outcome</th><th>Results</th><th>Comments</th></tr>
<tr>
<td></td>
<td></td>
<td>(no drop-out for registry data)
<u>control:</u> 67.3%
<u>inspection:</u> 64.2%
<u>guidance:</u> 64.1%

Work characteristics: home-care service workers</td>
<td>challenges for employees related to own work environments. Inspectors instructed to provide advice on these challenges based on relevant OSH legislation and regulations
Control: “care as usual”, no inspection/ no workshop

Study groups:
2 intervention groups: 1) labour inspection and 2) guidance; 1 control group (originally 3 intervention groups planned but</td>
<td>psychological diagnoses (P-codes): total number of days of sick leave: counted and added together all days of sick leave due to the diagnoses of interest for 18 months post-interventions
number of sick leave periods: total number of sick leaves due to the diagnoses of interest for 18 months post-interventions

Days and sick leaves due to L and/or P diagnoses in 12 months preceding interventions to measure baseline sickness absence

Time of measurement:
<u>Questionnaire data:</u> baseline, at 6 and at 12 months after intervention</td>
<td>
<table>
<tr><td>General health (0-4)</td><td>2.09 (0.82)</td><td>2.10 (0.81)</td><td>2.09 (0.82)</td></tr>
<tr><td>Mental distress (1-4)</td><td>1.35 (0.51)</td><td>1.38 (0.51)</td><td>1.43 (0.54)</td></tr>
<tr><td>General pain (0-10)</td><td>3.18 (2.35)</td><td>3.28 (2.41)</td><td>3.30 (2.34)</td></tr>
<tr><td>Headache (1-4)</td><td>1.83 (0.88)</td><td>1.89 (0.81)</td><td>1.85 (0.85)</td></tr>
<tr><td>Neck pain (1-4)</td><td>1.90 (0.86)</td><td>1.87 (0.85)</td><td>1.95 (0.91)</td></tr>
<tr><td>Pain in shoulder & upper arm (1-4)</td><td>1.89 (0.88)</td><td>1.94 (0.89)</td><td>1.97 (0.95)</td></tr>
<tr><td>Back pain (1-4)</td><td>1.98 (0.92)</td><td>1.98 (0.84)</td><td>2.02 (0.89)</td></tr>
<tr><td>Pain in hands, wrist or lower arm (1-4)</td><td>1.56 (0.81)</td><td>1.54 (0.81)</td><td>1.64 (0.88)</td></tr>
<tr><td>Pain in lower extremities</td><td>1.95 (0.92)</td><td>1.93 (0.90)</td><td>1.85 (0.90)</td></tr>
</table>

Tab.3: Linear mixed models for health outcomes
<table>
<tr><td></td><td colspan="2">1rst follow-up</td><td colspan="2">2nd follow-up</td><td>ICC</td></tr>
<tr><td></td><td>Inspec-tion</td><td>Guid-ance</td><td>Inspec-tion</td><td>Guid-ance</td><td></td></tr>
<tr><td></td><td>Coef. 95% CI</td><td>Coef. 95% CI</td><td>Coef. 95% CI</td><td>Coef. 95% CI</td><td></td></tr>
<tr><td>General health</td><td>-0.06 (-0.18-0.05)</td><td>-0.10 (-0.22-0.01)</td><td>-0.15 (-0.29- -0.01)</td><td>-0.12 (-0.26-0.02)</td><td>0.019</td></tr>
<tr><td>Mental distress</td><td>-0.07 (-0.15-0.01)</td><td>-0.07 (-0.15-0.01)</td><td>0.03 (-0.06-0.12)</td><td>-0.02 (-0.12-0.07)</td><td><0.001</td></tr>
<tr><td>General pain</td><td>-0.01 (-0.36-0.34)</td><td>-0.14 (-0.49-0.20)</td><td>-0.20 (-0.62-0.22)</td><td>-0.01 (-0.42-0.42)</td><td>0.021</td></tr>
</table>
</td>
<td></td>
</tr>
</table>

<table>
<tr><th>General</th><th>Study</th><th>Population</th><th>Exposure</th><th>Outcome</th><th>Results</th><th>Comments</th></tr>
<tr><td></td><td></td><td></td><td>lower participation rate than expected)

Time of measurement:
May – October 2019</td><td><u>Sick-leave data:</u>
1 January 2018 – 30 June 2021

Steingrímsdóttir OA, Vøllestad NK, Røe C, Knardahl S. Variation in reporting of pain and other subjective health complaints in a working population and limitations of single sample measurements. Pain 2004 Jul;110(12): 130–9.</td><td>
<table>
<tr><td>Headache</td><td>0.04 (-0.08-0.16)</td><td>-0.01 (-0.13-0.11)</td><td>-0.02 (-0.16-0.12)</td><td>0.09 (-0.05-0.24)</td><td>0.011</td></tr>
<tr><td>Neck pain</td><td>0.05 (-0.06-0.17)</td><td>-0.04 (-0.17-0.07)</td><td>-0.06 (-0.21-0.09)</td><td>-0.07 (-0.23-0.08)</td><td><0.001</td></tr>
<tr><td>Pain in shoulder & upper arm</td><td>0.05 (-0.07-0.18)</td><td>-0.01 (-0.13-0.12)</td><td>-0.12 (-0.28-0.03)</td><td>-0.14 (-0.31-0.01)</td><td><0.001</td></tr>
<tr><td>Back pain</td><td>-0.02 (-0.15-0.11)</td><td>-0.12 (-0.25-0.01)</td><td>-0.09 (-0.25-0.06)</td><td>0.02 (-0.13-0.18)</td><td>0.009</td></tr>
<tr><td>Pain in hands, wrist or lower arm</td><td>0.07 (-0.05-0.20)</td><td>0.05 (-0.07-0.17)</td><td>-0.01 (-0.15-0.15)</td><td>-0.02 (-0.18-0.13)</td><td>0.015</td></tr>
<tr><td>Pain in lower extremities</td><td>0.01 (-0.11-0.14)</td><td>-0.10 (-0.23-0.03)</td><td>-0.03 (-0.20-0.12)</td><td>0.10 (-0.05-0.27)</td><td><0.001</td></tr>
</table>
ICC=interclass correlation coefficient; SD=standard deviation; Coef=coefficient; CI=confidence intervals

Tab.4: Mixed negative binomial regression
<table>
<tr><td></td><td>Total number of days of sick leave</td><td>Total number of sick leave products</td></tr>
<tr><td></td><td>IRR (95% CI)</td><td>IRR (95% CI)</td></tr>
<tr><td colspan="3">Musculoskeletal and psychological diagnoses</td></tr>
<tr><td>Inspection</td><td>0.89 (0.50-1.59)</td><td>0.93 (0.72-1.21)</td></tr>
<tr><td>Guidance</td><td>0.98 (0.54-1.76)</td><td>1.05 (0.81-1.36)</td></tr>
<tr><td colspan="3">Musculoskeletal diagnoses</td></tr>
<tr><td>Inspection</td><td>0.82 (0.42-1.60)</td><td>0.86 (0.63-1.16)</td></tr>
<tr><td>Guidance</td><td>0.94 (0.47-1.86)</td><td>0.95 (0.70-1.29)</td></tr>
<tr><td colspan="3">Psychological diagnoses</td></tr>
<tr><td>Inspection</td><td>1.08 (0.35-3.31)</td><td>1.13 (0.68-1.85)</td></tr>
<tr><td>Guidance</td><td>1.16 (0.36-3.69)</td><td>1.32 (0.80-2.18)</td></tr>
</table>

Short summary of findings:
- no statistically significant effects of either intervention on the self-reported employee health outcomes
- fewer sick leave days and periods due to</td><td></td></tr>
</table>

<table>
<tr><th>General</th><th>Study</th><th>Population</th><th>Exposure</th><th>Outcome</th><th>Results</th><th>Comments</th></tr>
<tr><td></td><td></td><td></td><td></td><td></td><td>musculoskeletal diagnoses and more sick leave days and periods due to psychological diagnoses after the interventions for both interventions (but not significant)</td><td></td></tr>
<tr><td>Finager Garshol 2022 #4412</td><td>See above</td><td>See above</td><td>See above</td><td>Outcome name:
Psychosocial work factors

Biomechanical work factors

Outcome definition and assessment:
<u>Psychosocial work factors:</u>
General Questionnaire for Psychological and Social Factors at Work (QPS^{Nordic}): quantitative demands, decision demands,
learning demands, role clarity, role conflict, decision control,
control over work intensity, positive challenges at work, fair
leadership, empowering leadership, support from immediate superior, support from coworkers,</td><td>Descriptive Statistics:

Tab.1: Descriptives job title
<table>
<tr><th></th><th>Inspection</th><th>Guidance</th><th>Control</th></tr>
<tr><td></td><td>N(%) or mean (SD)</td><td>N(%) or mean (SD)</td><td>N(%) or mean (SD)</td></tr>
<tr><td colspan="4">Job title</td></tr>
<tr><td>Nurse</td><td>190 (37.2)</td><td>154 (32.2)</td><td>229 (35.2)</td></tr>
<tr><td>Nurse assistant</td><td>230 (45.0)</td><td>226 (47.2)</td><td>283 (43.5)</td></tr>
<tr><td>Other health professions</td><td>10 (2.0)</td><td>9 (1.9)</td><td>14 (2.2)</td></tr>
<tr><td>Other care staff</td><td>64 (12.5)</td><td>75 (15.7)</td><td>105 (16.1)</td></tr>
<tr><td>Leader</td><td>13 (2.5)</td><td>13 (2.7)</td><td>15 (2.3)</td></tr>
<tr><td>Other</td><td>4 (0.8)</td><td>2 (0.4)</td><td>5 (0.8)</td></tr>
</table>

Unadjusted/ adjusted estimates with precision (e.g. 95% confidence interval) for each outcome:

Tab2+3+4: Baseline mean values
<table>
<tr><th></th><th>Inspection Mean (SD)</th><th>Guidance Mean (SD)</th><th>Control Mean (SD)</th></tr>
<tr><td>Quantitative demands*</td><td>2.96 (0.76)</td><td>2.97 (0.73)</td><td>3.08 (0.78)</td></tr>
<tr><td>Decision demands*</td><td>3.57 (0.65)</td><td>3.58 (0.65)</td><td>3.62 (0.64)</td></tr>
<tr><td>Learning demands*</td><td>2.52 (0.58)</td><td>2.52 (0.60)</td><td>2.54 (0.59)</td></tr>
<tr><td>Role clarity**</td><td>4.37 (0.60)</td><td>4.28 (0.63)</td><td>4.27 (0.70)</td></tr>
<tr><td>Role conflict**</td><td>2.64 (0.79)</td><td>2.58 (0.79)</td><td>2.68 (0.85)</td></tr>
<tr><td>Decision control**</td><td>2.79 (0.73)</td><td>2.75 (0.67)</td><td>2.80 (0.70)</td></tr>
<tr><td>Control over work intensity**</td><td>2.29 (0.85)</td><td>2.23 (0.76)</td><td>2.36 (0.87)</td></tr>
<tr><td>Positive challenges at work**</td><td>4.20 (0.66)</td><td>4.20 (0.60)</td><td>4.19 (0.63)</td></tr>
</table></td><td>See above</td></tr>
</table>

<table>
<tr><th>General</th><th>Study</th><th>Population</th><th>Exposure</th><th>Outcome</th><th>Results</th><th>Comments</th></tr>
<tr><td></td><td></td><td></td><td></td><td>focus on human resources
and predictability in the coming month
->measured with 3-5 items (1 to 5, 1=very seldom or never, 2=seldom, 3=sometimes, 4=often and 5=very often or always)
+ 5 items to measure experience of adverse social behaviour over last 6 months: bullying by coworkers, bullying by superior, violence, threats and unwanted sexual attention (1=never to 5= yes, on a daily basis)

<u>Biomechanical work factors:</u>
1) 5 items developed by Statistics Norway: time spent at work squatting/kneeling, standing, leaning forward without</td><td><table>
<tr><td>Fair leadership**</td><td>4.00 (0.85)</td><td>4.11 (0.79)</td><td>3.99 (0.83)</td></tr>
<tr><td>Empowering leadership**</td><td>3.22 (1.05)</td><td>3.27 (1.00)</td><td>3.18 (1.01)</td></tr>
<tr><td>Support from immediate superior **</td><td>3.81 (1.02)</td><td>3.89 (0.97)</td><td>3.71 (1.02)</td></tr>
<tr><td>Support from coworkers**</td><td>4.27 (0.80)</td><td>4.24 (0.80)</td><td>4.17 (0.82)</td></tr>
<tr><td>Focus on human resources**</td><td>2.95 (0.98)</td><td>3.12 (0.91)</td><td>2.97 (0.96)</td></tr>
<tr><td>Predictability in coming month**</td><td>3.49 (0.97)</td><td>3.50 (0.90)</td><td>3.47 (0.95)</td></tr>
<tr><td>Bullying -coworkers*</td><td>1.16 (0.50)</td><td>1.21 (0.55)</td><td>1.22 (0.58)</td></tr>
<tr><td>Bullying -superior*</td><td>1.11 (0.45)</td><td>1.12 (0.45)</td><td>1.12 (0.42)</td></tr>
<tr><td>Violence*</td><td>1.17 (0.50)</td><td>1.17 (0.51)</td><td>1.15 (0.45)</td></tr>
<tr><td>Threats*</td><td>1.14 (0.43)</td><td>1.17 (0.49)</td><td>1.14 (0.41)</td></tr>
<tr><td>Unwanted sexual attention*</td><td>1.32 (0.68)</td><td>1.41 (0.72)</td><td>1.38 (0.74)</td></tr>
<tr><td>Time spent – squatting/ kneeling*</td><td>2.67 (1.04)</td><td>2.83 (1.06)</td><td>2.85 (1.17)</td></tr>
<tr><td>Time spent – walking or upright position*</td><td>4.64 (1.23)</td><td>4.59 (1.20)</td><td>4.49 (1.35)</td></tr>
<tr><td>Time spent – leaning forward without support*</td><td>3.12 (1.23)</td><td>3.08 (1.27)</td><td>3.09 (1.32)</td></tr>
<tr><td>Lifting in uncomfortable position*</td><td>2.06 (0,.71)</td><td>2.10 (1.27)</td><td>2.03 (0.75)</td></tr>
<tr><td>Lifting more than 10 kg*</td><td>1.97 (0.76)</td><td>2.02 (0.81)</td><td>1.92 (0.77)</td></tr>
<tr><td>Manually moving patients/clients*</td><td>2.09 (0.75)</td><td>2.04 (0.73)</td><td>2.02 (0.74)</td></tr>
<tr><td>Lifting/ support patients/ clients*</td><td>2.15 (0.79)</td><td>2.11 (0.73)</td><td>2.08 (0.75)</td></tr>
<tr><td>Physical exertions without aids*</td><td>1.81 (0.69)</td><td>1.78 (0.68)</td><td>1.75 (0.67)</td></tr>
<tr><td>Physical exertions despite available aids*</td><td>1.55 (0.61)</td><td>1.55 (0.61)</td><td>1.55 (0.69)</td></tr>
<tr><td>Perceived physical intensity/ demand at work*</td><td>3.71 (1.93)</td><td>3.91 (2.15)</td><td>3.86 (2.19)</td></tr>
<tr><td>Appropriate allocation pf physically demanding work**</td><td>2.83 (1.09)</td><td>2.81 (1.10)</td><td>2.79 (1.08)</td></tr>
</table>
*lower score is advantageous; **higher score is advantageous</td><td></td></tr>
</table>

General	Study	Population	Exposure	Outcome	Results	Comments
				support, awkward lifting and heavy lifting items 1, 2 and 3 rated from 1=never to 6=almost all the time items 4 and 5 rated from 1=never to 5=at least 20 times a day 2) 4 items from Smedley et al. 1995: how often in a shift (1) manually transfer clients/ patients between a bed and a chair; (2) manually move clients/patients around on a bed, chair or wheelchair; (3) perform physically demanding tasks without the use of aids; and (4) perform physically demanding tasks without the use of aids, despite them being available 1=never to 5=at least 20 times a day	Tab.2: Linear mixed models for effect of intervention on work factors (table below)	

Tab.2: Linear mixed models for effect of intervention on work factors

	1rst follow-up		2nd follow-up		ICC
	Inspec-tion	Guid-ance	Inspec-tion	Guid-ance	
	Coef. 95% CI	Coef. 95% CI	Coef. 95% CI	Coef. 95% CI	
Quantitative demands*	0.05 (-0.06-0.17)	-0.02 (-0.14-0.08)	0.11 (-0.02-0.24)	-0.01 (-0.13-0.13)	0.104
Decision demands*	0.01 (-0.08-0.09)	-0.02 (-0.11-0.06)	-0.06 (-0.17-0.04)	-0.02 (-0.13-0.08)	0.019
Learning demands*	0.04 (-0.03-0.11)	-0.01 (-0.09-0.06)	0.06 (-0.03-0.16)	-0.01 (-0.10-0.09)	0.008
Role clarity**	0.01 (-0.07-0.10)	0.06 (-0.02-0.14)	-0.05 (-0.16-0.06)	0.03 (-0.07-0.14)	0.006
Role conflict**	-0.01 (-0.13-0.11)	-0.07 (-0.01-0.17)	0.10 (-0.04-0.25)	0.07 (-0.07-0.22)	0.029
Decision control**	-0.07 (-0.16-0.01)	0.08 (-0.01-0.17)	0.01 (-0.10-0.11)	0.16 (0.04-0.27)	0.002
Control over work intensity**	0.01 (-0.09-0.10)	0.14 (0.03-0.24)	-0.01 (-0.14-0.08)	0.16 (0.04-0.29)	0.001
Positive challenges at work**	0.01 (-0.07-0.10)	0.04 (-0.04-0.13)	-0.03 (-0.14-0.08)	0.03 (-0.07-0.15)	0.010
Fair leadership**	-0.05 (-0.19-0.08)	0.11 (-0.02-0.25)	-0.05 (-0.22-0.11)	0.07 (-0.07-0.22)	0.040
Empowering leadership**	-0.01 (-0.18-0.15)	0.10 (-0.06-0.26)	-0.05 (-0.25-0.14)	0.23 (0.03-0.43)	0.053
Support from immediate superior **	-0.06 (-0.22-0.10)	0.08 (-0.08-0.24)	-0.13 (-0.32-0.06)	0.16 (-0.03-0.24)	0.049
Support from coworkers**	-0.01 (-0.11-0.10)	-0.01 (-0.11-0.10)	-0.05 (-0.18-0.08)	0.10 (-0.03-0.36)	0.001
Focus on human resources**	0.01 (-0.14-0.17)	0.02 (-0.13-0.17)	-0.13 (-0.31-0.05)	0.02 (-0.16-0.21)	0.046

General	Study	Population	Exposure	Outcome	Results						Comments
				3) 1 item from Borg et al. 1990: perceived physical intensity while at work (1=not at all; 10=extremely heavy)<br><br>Smedley J, Egger P, Cooper C, et al. Manual handling activities and risk of low back pain in nurses. Occup Environ Med 1995;52:160–3<br><br>Borg G. Psychophysical scaling with applications in physical work and the perception of exertion. Scand J Work Environ Health 1990;16 Suppl 1:55–8.<br><br>**Time of measurement:** baseline, after 6 and 12 months	Predictability in coming month**	0.12 (-0.02-0.27)	0.02 (-0.12-0.17)	0.01 (-0.17-0.18)	-0.05 (-0.24-0.12)	0.033	
					Bullying - coworkers*	0.05 (-0.01-0.13)	0.02 (-0.05-0.10)	0.09 (-0.01-0.19)	-0.01 (-0.10-0.08)	0.001	
					Bullying - superior*	-0.01 (-0.07-0.04)	-0.02 (-0.09-0.06)	0.05 (-0.02-0.13)	-0.01 (-0.09-0.06)	0.001	
					Violence*	0.01 (-0.07-0.09)	0.03 (-0.05-0.11)	0.01 (0.09-0.10)	0.01 (-0.05-0.11)	0.035	
					Threats*	-0.05 (-0.11-0.01)	-0.03 (-0.0-0.03)	-0.04 (-0.12-0.03)	-0.04 (-0.12-0.03)	0.006	
					Unwanted sexual attention*	0.01 (-0.10-0.12)	0.05 (-0.05-0.16)	0.06 (-0.07-0.19)	0.08 (-0.04-0.22)	0.038	
					Time spent – squatting/ kneeling*	0.17 (0.02-0.32)	0.09 (-0.05-0.23)	-0.02 (-0.19-0.15)	0.02 (-0.14-0.20)	0.021	
					Time spent – walking or upright position*	0.06 (-0.12-0.25)	-0.16 (-0.35-0.02)	-0.01 (0.24-0.21)	-0.15 (-0.38-0.07)	0.010	
					Time spent – leaning forward without support*	-0.01 (-0.18-0.17)	-0.07 (-0.25-0.10)	0.01 (-0.21-0.22)	-0.01 (-0.23-0.19)	0.009	
					Lifting in uncomfortable position*	0.06 (-0.03-0.14)	0.02 (-0.06-0.11)	0.07 (-0.03-0.19)	0.11 (-0.01-0.22)	0.001	
					Lifting more than 10 kg*	0.04 (-0.06-0.15)	0.01 (-0.10-0.11)	0.03 (-0.10-0.16)	0.13 (-0.01-0.27)	0.001	
					Manually moving patients/clients*	-0.01 (-0.10-0.09)	-0.09 (-0.19-0.01)	0.01 (-0.10-0.14)	0.03 (-0.09-0.16)	0.003	
					Lifting/ support patients/ clients*	0.03 (-0.06-0.13)	0.01 (-0.08-0.11)	0.02 (-0.10-0.14)	-0.06 (-0.18-0.06)	0.001	
					Physical exertions without aids*	0.03 (-0.08-0.14)	-0.04 (-0.06-0.15)	-0.01 (-0.14-0.11)	-0.03 (-0.16-0.09)	0.051	

<table>
<tr><th>General</th><th>Study</th><th>Population</th><th>Exposure</th><th>Outcome</th><th>Results</th><th>Comments</th></tr>
<tr><td></td><td></td><td></td><td></td><td></td><td>
<table>
<tr><td>Physical exertions despite available aids*</td><td>0.07 (-0.02-0.17)</td><td>0.06 (-0.03-0.15)</td><td>0.01 (-0.10-0.13)</td><td>0.06 (-0.05-0.18)</td><td>0.001</td></tr>
<tr><td>Perceived physical intensity/ demand at work*</td><td>0.19 (-0.07-0.45)</td><td>-0.01 (-0.27-0.25)</td><td>0.26 (-0.05-0.57)</td><td>0.08 (-0.23-0.39)</td><td>0.011</td></tr>
<tr><td>Appropriate allocation pf physically demanding work**</td><td>0.01 (-0.18-0.20)</td><td>-0.03 (-0.23-0.15)</td><td>-0.07 (-0.31-0.15)</td><td>-0.07 (-0.31-0.16)</td><td>0.026</td></tr>
</table>
*lower score is advantageous; **higher score is advantageous
ICC=Intraclass correlation for municipal cluster

Short summary of findings:

- inspection intervention: no significant effects on the psychosocial factors, adverse social behaviour and biomechanical factors compared to control
- guidance-through-workshop group: increases in decision control and empowering leadership at 12 months and control over work intensity at 6 and 12 months compared with control group but not statistically significant (after adjusting for multiple testing); no significant effect on adverse social behaviour and biomechanical factors
</td><td></td></tr>
<tr><td>Johannessen et al. 2022 #2421</td><td>See above</td><td>See above</td><td>See above</td><td>Outcome name: Implemented measures

Outcome definition and assessment: Self-developed questions: Have you recently implemented, or are you in the near future planning to</td><td>Unadjusted/ adjusted estimates with precision (e.g. 95% confidence interval) for each outcome:
Tab.3: Associations between intervention groups and self-report of implemented measures, or such plans, among home-care services managers (logistic regression analyses)
<table>
<tr><th></th><th>N</th><th>Yes n (%)</th><th>Coef. (SE)</th><th>Sign.</th></tr>
<tr><td colspan="5">Have you implemented, or are you in near future planning to implement, measures to improve the working environment at your workplace?</td></tr>
<tr><td>Inspection</td><td>36</td><td>35 (97%)</td><td>2.12 (1.07)</td><td>0.048</td></tr>
<tr><td>Guidance</td><td>36</td><td>28 (78%)</td><td>-0.18 (0.53)</td><td>0.732</td></tr>
<tr><td>Controls</td><td>52</td><td>42 (81%)</td><td>Ref</td><td></td></tr>
<tr><td>Missing</td><td>6</td><td></td><td></td><td></td></tr>
<tr><td colspan="5">(If yes, what kind of measures) …identifying hazards and assessing the risks at your workplace?</td></tr>
<tr><td>Inspection</td><td>35</td><td>27</td><td>1.31 (0.51)</td><td>0.010</td></tr>
</table>
</td><td>See above</td></tr>
</table>

<table>
<tr><th>General</th><th>Study</th><th>Population</th><th>Exposure</th><th>Outcome</th><th>Results</th><th>Comments</th></tr>
<tr><td></td><td></td><td></td><td></td><td>implement, measures to improve the working environment at your workplace?” (yes”, “no” and “do not know)
<u>for control:</u> “yes, we have recently implemented measures”; “yes, we are in the process of implementing measures”; “yes, we are planning to implement measures by 2019”; “no”; “do not know”
-recoded into a dichotomous variable (yes/no)
-yes only -> follow-up questions: kind of measures</td><td><table>
<tr><td>Guidance</td><td>28</td><td>19</td><td>0.84 (0.51)</td><td>0.098</td></tr>
<tr><td>Controls</td><td>42</td><td>20</td><td></td><td></td></tr>
<tr><td colspan="5">(If yes, what kind of measures) …develop plans for a systematic approach to occupational safety and health management?</td></tr>
<tr><td>Inspection</td><td>35</td><td>13</td><td>0.17 (0.48)</td><td>0.727</td></tr>
<tr><td>Guidance</td><td>28</td><td>11</td><td>4.07 (0.51)</td><td>0.611</td></tr>
<tr><td>Controls</td><td>42</td><td>14</td><td></td><td></td></tr>
</table>
Short summary of findings:
- managers in the inspection group reported significantly more frequently to have implemented, or having plans to soon implement, preventive measures and measures of hazards identification and risk assessment as compared to control
- no effect in guidance group</td><td></td></tr>
<tr><td>First Author, Year
McLeod, Christopher 2024
#2162</td><td>Study name: -

Country: Alberta, Canada</td><td>Recruitment method/ Data source: data from Government of Alberta (GoA) Ministry of Jobs, Economy and Trade and Workers Compensation Board of Alberta (WCB)</td><td>Description of intervention: inspections

Study groups:</td><td>Outcome name: Injuries (claims)

Outcome definition and assessment:
All accepted claims for work-related</td><td>Descriptive Statistics:
Supplementary Table A. Number of firm-year months eligible for inspection in the final analytical cohorts by study restrictions and sector<table>
<tr><td></td><td>Construction</td><td>Manufacturing</td><td>Transportation</td></tr>
<tr><td>Initial cohort</td><td>5,610,337</td><td>1,430,598</td><td>2,040,606</td></tr>
</table></td><td>Funding:
Government of Alberta OHS Futures—Research Funding Program, grant number: 095233766</td></tr>
</table>

<table>
<tr><th>General</th><th>Study</th><th>Population</th><th>Exposure</th><th>Outcome</th><th>Results</th><th>Comments</th></tr>
<tr>
<td>McLeod, Christopher, 2021 (# reference list final report)

Extracted by: MS

Checked by: UBA</td>
<td>Study design: difference-in-differences (DID) study design (=controlled before-after study design)
Time of Study: 2011-2018</td>
<td>- linking of data via WCB account number and industry operation identifier
- GoA data extracted from Compliance Management Information System (CMIS) database
-inclusion criteria:
1) having at least one or more full-time equivalent employees (FTE) for 37 consecutive months
2) having a preintervention baseline (i.e. no inspection during 12 months before intervention)
3) no investigations taking place during same month as inspection

Study design: 37-month time frame divided into four distinct periods:
12-month preintervention period, 1-month intervention period (firm experienced regulatory inspection or not), and two 12-month postintervention periods

Matching criteria:
Matching of inspected firm- to non-inspected firm-year months based on firm-level characteristics (multivariable logit prediction models and model fit statistics of the Akaike information criterion</td>
<td>Intervention: inspected firm
Control: non-inspected firm

Repeated cross sections of firms in which firms could be in the treatment (inspected firm) or control (non-inspected firm) groups at different time periods

Time of measurement: 1 month intervention period</td>
<td>injuries and musculo-skeletal diseases, with work disability (compensated lost-time) beyond the day of injury
-exclusion of occupational cancer and mental health

Time of measurement: 2x12 months after intervention</td>
<td>
<table>
<tr><td>Firms 1+ FTEs for 37 months*</td><td>1,145,035</td><td>404,692</td><td>323,226</td></tr>
<tr><td>Firms no regulatory activities in past 12 months</td><td>887,919</td><td>315,176</td><td>263,922</td></tr>
<tr><td>Firms no investigation during month of inspection**</td><td>887,370</td><td>314,950</td><td>263,812</td></tr>
<tr><td>Inspected firms***</td><td>3,835</td><td>1,154</td><td>303</td></tr>
<tr><td>Control firms</td><td>883,535</td><td>313,796</td><td>263,509</td></tr>
<tr><td>Matched</td><td></td><td></td><td></td></tr>
<tr><td>Inspected firms***</td><td>3,827</td><td>1,133</td><td>295</td></tr>
<tr><td>Control firms</td><td>452,741</td><td>82,657</td><td>81,828</td></tr>
</table>

Statistical methods used: difference-in-differences (DiD) negative binomial regression models with log of firm FTEs included as an offset variable and clustered by the firm to account for correlation of observations within firms over time. Balancing of firm characteristics between the treatment group and control groups at the time of the intervention using Coarsened Exact Matching (CEM).
Matching variables included in regression models (except for lost-time injury claim in the previous 12 months) + firm tenure, whether firm was a member of the Partnerships in Injury Reduction (PIR), they had Certificate of Recognition (COR), and whether had head office in Alberta
- sector specific approach: construction, manufacturing, and transportation

Unadjusted/ adjusted estimates with precision (e.g. 95% confidence interval) for each outcome:</td>
<td>Conflict of Interest stated: yes

Other comments:
- Ethical approval from Behavioural Research Ethics Board of the University of British Columbia (H18-00702)
- no differentiation according to the type of disease (injury or musculoskeletal disease)</td>
</tr>
</table>

<table>
<tr><th>General</th><th>Study</th><th>Population</th><th>Exposure</th><th>Outcome</th><th>Results</th><th>Comments</th></tr>
<tr><td></td><td></td><td>and Bayesian information criterion): insurable earnings (categorical, quartiles); firm size (categorical); industry premium rate; experience rate adjustment (categorical: discounted, unadjusted, surcharged); industry subsector (derived from SIC 1980 division, major and minor group codes); rate year, and a binary indicator of whether the firm had a lost-time injury claim in the previous 12 months.

invited companies: NA

companies at baseline: NA

Response: NA

companies at follow –up: NA

Loss-to-follow-up: NA

Work characteristics: construction, transport, manufacturing</td><td></td><td></td><td>Tab.2: Claim rate ratio of the effect of inspection on firm-level lost-time injury rates
<table>
<tr><th></th><th>Construction</th><th>Manufacturing</th><th>Transportation</th></tr>
<tr><td></td><td>RR 95% CI</td><td>RR 95% CI</td><td>RR 95% CI</td></tr>
<tr><td>Baseline difference</td><td>1.08 (1.01-1.14)</td><td>1.08 (0.99-1.17)</td><td>1.01 (0.84-1.20)</td></tr>
<tr><td>Postintervention period (t + 1)</td><td>0.92 (0.90-0.94)</td><td>0.95 (0.92-0.98)</td><td>1.05 (0.98-1.12)</td></tr>
<tr><td>Postintervention period (t + 2)</td><td>0.96 (0.93-0.98)</td><td>0.89 (0.86-0.93)</td><td>1.12 (1.04-1.20)</td></tr>
<tr><td>Intervention effect (t + 1)</td><td>1.12 (1.04-1.21)</td><td>1.01 (0.91-1.11)</td><td>1.07 (0.88-1.31)</td></tr>
<tr><td>Intervention effect (t + 2)</td><td>1.02 (0.94-1.10)</td><td>1.00 (0.90-1.11)</td><td>0.92 (0.75-1.13)</td></tr>
<tr><td>Firm-years</td><td>1,369,704</td><td>251,370</td><td>246,369</td></tr>
</table>
RR=Rate ratio;
Firms matched during the intervention month on insurable earnings, firm size, industry premium rate, experience rate adjustment, and industry subsector. Postintervention periods = postintervention periods for both firms that were inspected during month t0 and firms that were not inspected during month t0. Firm years=12-month periods prior (t−1) to the intervention month (t0), the 12-month period after that (t + 1), and the 12-month period after that (t + 2). Models adjusted for insurable earnings, firm size, industry premium rate, experience rate adjustment, industry subsector, rate year, firm tenure, firm membership in Partnerships in Injury Reduction, Certificate of Recognition, and employer address location.

Tab.3: Claim rate ratio of the effect of inspection on firm-level lost-time injury rates using matched cohorts by sector
<table>
<tr><th></th><th>M I
RR 95%CI</th><th>M II
RR 95%CI</th><th>M III
RR 95%CI</th><th>M IV
RR 95%CI</th><th>M V
RR 95%CI</th></tr>
<tr><td>Contruction</td><td></td><td></td><td></td><td></td><td></td></tr>
<tr><td>Inspected (t0)</td><td>1.08 (1.01–1.14)</td><td>0.91 (0.83–0.99)</td><td>1.03 (0.94–1.12)</td><td>1.03 (0.94–1.12)</td><td>1.03 (0.94–1.12)</td></tr>
<tr><td>Inspected more than once (t0)</td><td></td><td>1.43 (1.27–1.63)</td><td></td><td></td><td></td></tr>
<tr><td>Inspected with orders issued (t0)</td><td></td><td></td><td>1.10 (0.97–1.23)</td><td>1.08 (0.94–1.23)</td><td>1.11 (0.97–1.27)</td></tr>
<tr><td>Inspected with stop work/use orders issued (t0)</td><td></td><td></td><td></td><td>1.05 (0.89–1.23)</td><td></td></tr>
</table></td><td></td></tr>
</table>

General	Study	Population	Exposure	Outcome	Results	Comments

Inspected with fall protection orders issued (t0)					0.96 (0.82–1.14)
Postintervention period (t + 1)	0.92 (0.90–0.94)	0.93 (0.91–0.94)	0.92 (0.90–0.94)	0.92 (0.90–0.94)	0.92 (0.90–0.94)
Postintervention period (t + 2)	0.96 (0.93–0.98)	0.96 (0.94–0.98)	0.96 (0.93–0.98)	0.96 (0.93–0.98)	0.96 (0.93–0.98)
Intervention effect (t + 1)	1.12 (1.04–1.21)	1.08 (0.98–1.18)	1.16 (1.05–1.28)	1.12 (0.95–1.31)	1.22 (1.03–1.44)
Intervention effect (t + 2)	1.02 (0.94–1.10)	0.94 (0.84–1.04)	1.06 (0.95–1.18)	1.02 (0.85–1.22)	1.03 (0.86–1.24)
Firm-years	1,369,704				
Manufacturing					
Inspected (t0)	1.08 (0.99–1.17)	0.95 (0.84–1.07)	1.01 (0.88–1.15)	1.01 (0.88–1.15)	1.01 (0.88–1.15)
Inspected more than once (t0)		1.25 (1.08–1.45)			
Inspected with orders issued (t0)			1.11 (0.95–1.31)	1.10 (0.93–1.30)	1.08 (0.91–1.29)
Inspected with stop work/use orders issued (t0)				1.13 (0.84–1.53)	
Inspected with fall protection orders issued (t0)					1.08 (0.88–1.33)
Postintervention period (t + 1)	0.95 (0.92–0.98)	0.94 (0.92–0.97)	0.95 (0.92–0.98)	0.95 (0.92–0.98)	0.95 (0.92–0.98)
Postintervention period (t + 2)	0.89 (0.86–0.93)	0.89 (0.85–0.92)	0.89 (0.86–0.93)	0.89 (0.86–0.93)	0.89 (0.86–0.93)
Intervention effect (t + 1)	1.01 (0.91–1.11)	1.03 (0.91–1.16)	0.99 (0.87–1.12)	1.06 (0.74–1.50)	1.08 (0.87–1.33)
Intervention effect (t + 2)	1.00 (0.90–1.11)	1.04 (0.91–1.18)	0.98 (0.86–1.12)	1.07 (0.77–1.47)	1.03 (0.81–1.30)
Firm-years	251,370				

General	Study	Population	Exposure	Outcome	Results	Comments
					(see below)	

Transportation					
Inspected (t0)	1.01 (0.84–1.20)	0.82 (0.65–1.02)	0.94 (0.71–1.24)	0.94 (0.71–1.24)	0.94 (0.71–1.24)
Inspected more than once (t0)		1.62 (1.18–2.23)			
Inspected with orders issued (t0)			1.12 (0.80–1.58)	1.13 (0.80–1.59)	1.20 (0.83–1.73)
Inspected with stop work/use orders issued (t0)				0.92 (0.43–1.97)	
Inspected with fall protection orders issued (t0)					0.86 (0.56–1.33)
Postintervention period (t + 1)	1.05 (0.98–1.12)	1.06 (1.00–1.13)	1.05 (0.98–1.12)	1.05 (0.98–1.12)	1.05 (0.98–1.12)
Postintervention period (t + 2)	1.12 (1.04–1.20)	1.14 (1.06–1.23)	1.12 (1.04–1.20)	1.12 (1.04–1.20)	1.12 (1.04–1.20)
Intervention effect (t + 1)	1.07 (0.88–1.31)	0.90 (0.68–1.19)	1.15 (0.92–1.44)	1.40 (0.60–3.24)	1.34 (0.92–1.96)
Intervention effect (t + 2)	0.92 (0.75–1.13)	0.72 (0.53–0.99)	0.87 (0.67–1.12)	1.43 (0.41–4.94)	1.00 (0.69–1.44)
Firm-years	246,369				

Main intervention effects for inspections (Model I) estimated with interaction term between baseline (inspected (t0) and postintervention periods (t + 1 or t + 2). Intervention effects for specific types of inspections (Models II–V) estimated with linear combination of estimates of the separate interaction terms for each inspection type.

Tab. Suppl. D: Claim rate ratio of the effect of inspection on firm-level lost-time injury rates using matched cohorts by sector

	M I RR 95%CI	M II RR 95%CI	M III RR 95%CI	M IV RR 95%CI	M V RR 95%CI
Contruction					
Inspected (t0)	1.10 (1.05-1.16)	0.96 (0.89-1.03)	1.08 (1.00-1.16)	1.08 (1.00-1.16)	1.08 (1.00-1.16)
Inspected more than once (t0)		1.37 (1.24-1.52)			

General	Study	Population	Exposure	Outcome	Results	Comments
					(continued below)	

Inspected with orders issued (t0)			1.05 (0.95-1.17)	1.04 (0.92-1.16)	
Inspected with stop work/use orders issued (t0)				1.04 (0.90-1.21)	
Inspected with fall protection orders issued (t0)					0.99 (0.85-1.16)
Postintervention period (t + 1)	0.96 (0.94-0.98)	0.96 (0.94-0.98)	0.96 (0.94-0.98)	0.96 (0.94-0.98)	0.96 (0.94-0.98)
Postintervention period (t + 2)	0.98 (0.96-1.00)	0.98 (0.96-1.00)	0.98 (0.96-1.00)	0.98 (0.96-1.00)	0.98 (0.96-1.00)
Intervention effect (t + 1)	1.05 (0.99-1.12)	1.04 (0.97-1.12)	1.09 (1.00-1.18)	1.06 (0.92-1.22)	1.16 (0.99-1.34)
Intervention effect (t + 2)	1.01 (0.95-1.08)	0.95 (0.87-1.05)	1.03 (0.94-1.13)	0.98 (0.84-1.14)	0.99 (0.84-1.17)
Firm-years	1,369,704				
Manufacturing					
Inspected (t0)	1.13 (1.05-1.20)	1.04 (0.94-1.15)	1.08 (0.98-1.20)	1.08 (0.98-1.20)	1.08 (0.98-1.20)
Inspected more than once (t0)		1.15 (1.02-1..31)			
Inspected with orders issued (t0)			1.07 (0.94-1.21)	1.07 (0.93-1.22)	1.03 (0.90-1.18)
Inspected with stop work/use orders issued (t0)				1.02 (0.79-1.34)	
Inspected with fall protection orders issued (t0)					1.11 (0.93-1.32)
Postintervention period (t + 1)	0.98 (0.96-1.01)	0.97 (0.95-1.00)	0.98 (0.96-1.01)	0.98 (0.96-1.01)	0.98 (0.96-1.01)
Postintervention period (t + 2)	0.92 (0.89-0.95)	0.91 (0.88-0.94)	0.92 (0.89-0.95)	0.92 (0.89-0.95)	0.92 (0.89-0.95)
Intervention effect (t + 1)	0.98 (0.91-1.06)	1.02 (0.93-1.13)	0.99 (0.89-1.09)	1.13 (0.85-1.50)	1.00 (0.84-1.19)

General	Study	Population	Exposure	Outcome	Results	Comments
					(see below)	

Intervention effect (t + 2)	1.00 (0.92-1.09)	1.05 (0.94-1.18)	0.98 (0.87-1.09)	1.09 (0.84-1.42)	1.01 (0.83-1.24)
Firm-years	252,370				
Transportation					
Inspected (t0)	1.09 (0.93-1.28)	0.94 (0.77-1.14)	1.05 (0.83-1.33)	1.05 (0.83-1.33)	1.05 (0.83-1.33)
Inspected more than once (t0)		1.42 (1.07-1.88)			
Inspected with orders issued (t0)			1.07 (0.80-1.44)	1.07 (0.79-1.45)	1.13 (0.82-1.56)
Inspected with stop work/use orders issued (t0)				0.98 (0.53-1.82)	
Inspected with fall protection orders issued (t0)					0.88 (0.59-1.30)
Postintervention period (t + 1)	1.06 (1.00-1.12)	1.07 (1.01-1.13)	1.06 (1.00-1.12)	1.06 (1.00-1.12)	1.06 (1.00-1.12)
Postintervention period (t + 2)	1.09 (1.03-1.15)	1.10 (1.04-1.17)	1.09 (1.03-1.15)	1.09 (1.03-1.15)	1.09 (1.03-1.15)
Intervention effect (t + 1)	0.99 (0.84-1.16)	0.86 (0.68-1.09)	1.07 (0.88-1.30)	1.20 (0.60-2.40)	1.21 (0.90-1.61)
Intervention effect (t + 2)	0.91 (0.77-1.08)	0.75 (0.58-0.96)	0.91 (0.73-1.14)	1.40 (0.68-2.92)	0.96 (0.70-1.32)
Firm-years	246,369				

Main intervention effects for inspections (Model I) estimated with interaction term between baseline (inspected (t0) and postintervention periods (t + 1 or t + 2). Intervention effects for specific types of inspections (Models II–V) estimated with linear combination of estimates of the separate interaction terms for each inspection type.

Tab. Suppl. E: Incidence rate ratios with 95% confidence intervals of the effect of specific inspection on firm-level lost-time claim rates using matched cohorts, by sector and firm size (small firms < 20 FTEs, large Firms 20+ FTEs)

	Construction	Manufacturing	Transportation
	RR 95% CI	RR 95% CI	RR 95% CI
Small firms			
Inspected (t0)	1.04 (0.95-1.14)	1.12 (0.94-1.32)	1.09 (0.67-1.78)

Genera l	Study	Population	Exposure	Outcome	Results	Comments
					(see tables below)	

Postintervention period (t + 1)	0.84 (0.81-0.87)	0.84 (0.76-0.92)	0.89 (0.75-1.07)
Postintervention period (t + 2)	0.90 (0.87-0.93)	0.77 (0.79-0.86)	0.94 (0.78-1.14)
Intervention effect (t + 1)	1.25 (1.10-1.41)	1.07 (0.83-1.37)	1.01 (0.56-1.79)
Intervention effect (t + 2)	1.15 (1.01-1.32)	1.02 (0.79-1.31)	0.98 (0.56-1.73)
Firm-years	115,951	153,954	187,844
Large firms			
Inspected (t0)	1.10 (1.02-1.19)	1.07 (0.98-1.17)	0.99 (0.84-1.18)
Postintervention period (t + 1)	0.98 (0.96-1.01)	0.98 (0.95-1.01)	1.09 (1.03-1.15)
Postintervention period (t + 2)	1.00 (0.97-1.03)	0.92 (0.89-0.96)	1.14 (1.06-1.22)
Intervention effect (t + 1)	1.05 (0.96-1.15)	0.99 (0.89-1.10)	1.08 (0.90-1.30)
Intervention effect (t + 2)	0.94 (0.85-1.03)	1.00 (0.89-1.12)	0.90 (0.73-1.13)
Firm-years	213,789	97,416	58,525

Tab. Suppl. F: Incidence rate ratios with 95% confidence intervals of the effect of specific inspection on firm-level disabling injury claim rates using matched cohorts, by sector and firm size (small firms < 20 FTEs, large Firms 20+ FTEs)

	Construction	Manufacturing	Transportation
	RR 95% CI	RR 95% CI	RR 95% CI
Small firms			
Inspected (t0)	1.04 (0.95-1.13)	1.15 (0.99-1.34)	1.20 (0.75-1.91)
Postintervention period (t + 1)	0.86 (0.83-0.88)	0.87 (0.80-0.95)	1.00 (0.85-1.18)
Postintervention period (t + 2)	0.91 (0.88-0.94)	0.80 (0.72-0.89)	0.94 (0.82-1.09)
Intervention effect (t + 1)	1.22 (1.09-1.37)	1.02 (0.82-1.28)	0.88 (0.54-1.43)
Intervention effect (t + 2)	1.20 (1.06-1.35)	0.98 (0.78-1.23)	0.89 (0.54-1.46)
Firm-years	115,915	153,954	187,844
Large firms			
Inspected (t0)	1.14 (1.07-.22)	1.12 (1.04-1.20)	1.07 (0.93-1.24)
Postintervention period (t + 1)	1.02 (1.00-1.04)	1.00 (0.98-1.03)	1.07 (1.02-1.12)
Postintervention period (t + 2)	1.03 (1.00-1.05)	0.94 (0.91-0.98)	1.11 (1.05-1.18)

<table>
<tr><th>General</th><th>Study</th><th>Population</th><th>Exposure</th><th>Outcome</th><th>Results</th><th>Comments</th></tr>
<tr><td></td><td></td><td></td><td></td><td></td><td>
<table>
<tr><td>Intervention effect (t + 1)</td><td>0.97 (0.91-1.04)</td><td>0.97 (0.89-1.06)</td><td>1.01 (0.87-1.17)</td></tr>
<tr><td>Intervention effect (t + 2)</td><td>0.92 (0.85-1.00)</td><td>1.01 (0.92-1.10)</td><td>0.90 (0.76-1.07)</td></tr>
<tr><td>Firm-years</td><td>213,789</td><td>97,416</td><td>58,525</td></tr>
</table>

Short summary of findings:
- construction sector: inspected firms associated with a 12% increase in lost-time claim rate (IRR: 1.12, 95% CI: 1.04−1.21) relative to the change in non-inspected firms during first year, but no different in the second year (IRR: 1.02, 95% CI: 0.94−1.10), postintervention
- in the postintervention period, injury rate decreased overall in construction in the first (IRR: 0.92 [0.90–0.94]) and second (IRR: [0.96-0.98]) years, -> inspected firms had a higher baseline rate that increased slightly, while non-inspected firms experienced a decline.
- manufacturing and transportation sectors: no inspection intervention effect
Effect of specific types of inspections:
<u>- construction</u>:
a) Having more than one inspection not associated with decrease in lost-time claim rate in the first or second year, postintervention, compared to non-inspected firms during same period.
b) Inspections with orders: 16% increase in lost-time claim rate during the first year but no difference in second year, postintervention.
c) Inspections with stop work/use orders: not associated with any differences in the changes
d) Inspections with fall protection orders: 22% increase in the injury rate during the first year but no difference in the second year relative to change in non-inspected firms postintervention.
<u>transportation:</u> reduction in injury rate due to various inspection activities in the second year, postintervention but only for firms with more than one inspection (28% reduction in lost-time claim rate)
<u>manufacturing:</u> no effect
disabling injury claim rates: similar pattern
small firms/ large firms: increased lost-time claim rates among inspected small construction firms relative to non-inspected small construction firms during the same period
</td><td></td></tr>
</table>

<table>
<tr><th>General</th><th>Study</th><th>Population</th><th>Exposure</th><th>Outcome</th><th>Results</th><th>Comments</th></tr>
<tr><td></td><td></td><td></td><td></td><td></td><td>->"Inspections were not effective in reducing firm-level claim rates, with the exception of transportation firms with more than one inspection experiencing a 28% decrease in their claim rate in the second year post-inspection, relative to the change in non-inspected firms. In construction, inspected firms experienced a 12% increase in their claim rate in the first year post-inspection. No effect was observed in the manufacturing sector."</td><td></td></tr>
<tr>
<td>First Author, Year
McLeod, Kim 2019
reference list

Extracted by:

Checked by:</td>
<td>Study name:-

Country: Canada

Study design: controlled before-after study (DID, fixed effect)

Time of Study: 2003-2014</td>
<td>Recruitment method/ Data source:
WorkSafeBC; exclusion of firms inspected in the two years (i.e. 24 months, 2003-2004) prior to the start of follow-up
Firms only included if: only 1 location, at least 1 full-time equivalent (FTE) for 4 years (1 year baseline, 1 year intervention, 2 years follow-up); whole sample: N=327,482

Matching criteria: matched control group: 4-digit sector (industry) category for included firms, experience rating category, and firm size (continuous and categorical)

invited companies: NA

companies at baseline: Single location 1 + FTE: n=89,593, no inspection in previous 24 months: n=74,394

Response: NA</td>
<td>Description of intervention: Inspection (targeted= firms are chosen because of high risk of serious injury)

Study groups: intervention = new inspection; not random

Time of measurement: 2005-2012</td>
<td>Outcome name:
Acute injuries
Shortterm, longterm and fatal injuries

Outcome definition and assessment:
- acute injuries: defined as any claim for amputations, fractures, dislocations, concussions, cold or heat exposures, burns, abrasions/contusions/lacerations or hernias
Shortterm, longterm, fatal: regardless of nature
-Measured as the number of acute injuries each year, per firm per 100 FTE</td>
<td>Descriptive Statistics:
Tab. 20 Cohort characteristics:
<table>
<tr><th></th><th>All firms (2005-2012)</th><th>Single location, 1+FTE firms, 2005-2012, only firm-years with four year follow up and no inspection in past 24 months</th></tr>
<tr><td>Number of firm</td><td>327,482</td><td>74,394</td></tr>
<tr><td>Mean FTE</td><td>10.5</td><td>7.4</td></tr>
<tr><td>Sum FTE</td><td>16,156,464</td><td>2,771,491</td></tr>
<tr><td>Sume Acute Injuries</td><td>169,441</td><td>11,584</td></tr>
<tr><td>Mean acute Injury rate</td><td>1.05</td><td>0.42</td></tr>
<tr><td>Sum SLF</td><td>441,121</td><td>24,250</td></tr>
<tr><td>Mean SLF Injury Rate</td><td>2.73</td><td>0.87</td></tr>
</table>
Tab.24: Sample characteristics
<table>
<tr><th></th><th>All firms (2005-2012)</th><th>Single location, 1+FTE firms, 2005-2012, only firm-years with four year follow up and no inspection in past 24 months</th></tr>
<tr><td>Sum inspections</td><td>93,402</td><td>10,485</td></tr>
<tr><td>Sum orders</td><td>61,301</td><td>6,439</td></tr>
<tr><td>Sum warning letters</td><td>1,789</td><td>130</td></tr>
<tr><td>Sum penalties</td><td>1,264</td><td>78</td></tr>
<tr><td>Median penalty amount</td><td>$ 5,000.00</td><td>$ 4,562.67</td></tr>
</table>
Tab. 25. Results matching firm size, base premium rate, injury rate
<table>
<tr><th></th><th colspan="2">Unmatched</th><th colspan="2">Matched</th></tr>
<tr><td></td><td>Not inspected</td><td>Inspected</td><td>Not inspected</td><td>Inspected</td></tr>
<tr><td>Average Firm Size in Full Time Equivalents</td><td>7.0</td><td>10.6</td><td>9.3</td><td>10.3</td></tr>
<tr><td>Average Base Premium Rate in Dollars</td><td>1.11</td><td>3.19</td><td>2.98</td><td>3.00</td></tr>
</table></td>
<td>Funding: NR

Conflict of Interest stated: no

Other comments:
- dissertation
- ethics approval from University of British Columbia Office Of Research Ethics (H12-02635)
- WorkSafeBC inspections includes some activities, such as communication with employers that may not necessarily involve site visits (2008 – 2011: 75-77% inspection reports involved a worksite visit by a prevention officer)</td>
</tr>
</table>

General	Study	Population	Exposure	Outcome	Results	Comments
		# companies at follow –up: NA; Loss-to-follow-up: NA; Work characteristics: Fig. 10 Firms by sector (see below)		Time of measurement: 1-2 years after inspection	(see below)	

Population

# companies at follow –up: NA

Loss-to-follow-up: NA

Work characteristics:
Fig. 10 Firms by sector

	Full cohort	Final cohort
Service sector	48%	65%
Public sector	0.3%	0.1%
Trade	9%	13%
Transportation & warehousing	10%	3%
Construction	22%	10%
Manufacturing	5%	5%
Primary resources	5%	3%

Outcome

Time of measurement: 1-2 years after inspection

Results

Number of Firms Where Experience Rating is Discount or Equal to 1	332,636	8,259	7,972	7,972
Number of Firms Where Experience Rating is Surcharge	5,629	576	397	397

Statistical methods used: chapter V: Fixed effects along with coarsened exact matching for dealing with time invariant unobserved differences between the control and treatment groups

Unadjusted/ adjusted estimates with precision (e.g. 95% confidence interval) for each outcome:

1. *Comparison of 4 different analysis methods with WorkSafeBC data (chapter 4)*

Analysis of data using methods of previous studies:
Tab.19: Scholz-Gray and Haviland change models for acute and short-term, longterm, and fatal (SLF) injuries after inspection, unmatched and matched

	Injuries	Unmatched (95% CI)	Matched (95% CI)
Inspection in previous year	Acute	-7% (-7%, -8%)	-11% (-18%, -5%)
Inspection 2 years ago		-13% (-12%, -13%)	-3% (-12%, 6%)
Inspection in previous year	SFL	-7% (-6%, -7%)	-10% (-4%, 3%)
Inspection 2 years ago		-13% (-13%, -12%)	-5% (-13%, 3%)

- decrease in injuries in inspected firms for unmatched and matched analyses; larger effects for inspection in previous year for matched analysis

Haviland, A. M., Burns, R. M., Gray, W. B., Ruder, T., & Mendeloff, J. (2012). A new estimate of the impact of OSHA inspections on manufacturing injury rates, 1998–2005. *American Journal of Industrial Medicine*, *55*(11), 964-975.

Scholz, J. T., & Gray, W. B. (1990). OSHA enforcement and workplace injuries: A behavioral approach to risk assessment. *Journal of Risk and Uncertainty*, *3*, 283-305.

<table>
<tr><th>General</th><th>Study</th><th>Population</th><th>Exposure</th><th>Outcome</th><th>Results</th><th>Comments</th></tr>
<tr><td></td><td></td><td></td><td></td><td></td><td>
Tab. 20: Foley, Baggs, Silverstein: Results of GEE Poisson models as incident rate ratios, matched and unmatched
<table>
<tr><th></th><th>Injuries</th><th>Unmatched (95% CI)</th><th>Matched (95% CI)</th></tr>
<tr><td>Inspection in previous year</td><td rowspan="2">Acute</td><td>1.46 (0.84, 2.54)</td><td>1.24 (1.11-1.39)</td></tr>
<tr><td>Inspection 2 years ago</td><td>1.36 (1.23-1.50)</td><td>1.32 (1.16-1.50)</td></tr>
<tr><td>Inspection in previous year</td><td rowspan="2">SFL</td><td>1.35 (0.88-2.07)</td><td>1.23 (1.13-1.34)</td></tr>
<tr><td>Inspection 2 years ago</td><td>1.22 (1.13-1.32)</td><td>1.24 (1.11-1.38)</td></tr>
</table>
Exponentiated coefficients; 95% confidence intervals in brackets

-firms being inspected experience a significant increase in injuries

Foley, M., Fan, Z. J., Rauser, E., & Silverstein, B. (2012). The impact of regulatory enforcement and consultation visits on workers' compensation claims incidence rates and costs, 1999–2008. American Journal of Industrial Medicine, 55(11), 976-990.
Baggs, J., Silverstein, B., & Foley, M. (2003). Workplace health and safety regulations: Impact of enforcement and consultation on workers' compensation claims rates in Washington State. American Journal of Industrial Medicine, 43(5), 483-494.

Tab. 21: Levine et al.: Results of fixed effects models as incident rate ratios, matched and unmatched
<table>
<tr><th></th><th>Injuries</th><th>Unmatched (95% CI)</th><th>Matched (95% CI)</th></tr>
<tr><td>Inspection in previous year</td><td rowspan="2">Acute</td><td>1.03 (0.94-1.13)</td><td>1.16 (1.02-1.31)</td></tr>
<tr><td>Inspection 2 years ago</td><td>1.07 (1.00-1.14)</td><td>1.13 (0.98-1.29)</td></tr>
<tr><td>Inspection in previous year</td><td rowspan="2">SFL</td><td>1.09 (0.99-1.19)</td><td>1.03 (0.94-1.12)</td></tr>
<tr><td>Inspection 2 years ago</td><td>1.00 (0.91-1.11)</td><td>0.96 (0.87, 1.07)</td></tr>
</table>
Exponentiated coefficients; 95% confidence intervals in brackets

Increased incidence of injuries due to inspections (higher but not always statistically significant)

Levine, D. I., Toffel, M. W., & Johnson, M. S. (2012). Randomized government safety inspections reduce worker injuries with no detectable job loss. Science, 336(6083), 907-911.

Levine, D. I., Toffel, M. W., & Johnson, M. S. (2012). Randomized government safety inspections reduce worker injuries with no detectable job loss. Science, 336(6083), 907-911.
</td><td></td></tr>
</table>

General	Study	Population	Exposure	Outcome	Results	Comments

Tab. 22: Results of matched difference-in-differences models as incident rate ratios, acute and short term, long term, and fatal injuries

	Injuries	Matched (95% CI)
Inspection in previous year	Acute	0.98 (0.83-1.17)
Inspection 2 years ago		0.97 (0.81-1.17)
Inspection in previous year	SFL	0.95 (0.84-1.08)
Inspection 2 years ago		0.94 (0.83-1.07)

Exponentiated coefficients; 95% confidence intervals in brackets

-inspections have no significant effect on injury rates

Lee, M. J. (2016). *Matching, regression discontinuity, difference in differences, and beyond*. Oxford University Press.

-> most appropriate: fixed effects method -> used for studying effects of inspections in British Columbia

Effect of regulatory inspections on workplace injury rates in British Columbia (chapter 5)

Fig. 11+12: Effect of inspection on acute and short-term, long-term and fatal (SFL) injuries, by duration of follow-up and type of inspection (95% CI)

	Acute injury RR	SFL injury RR
12 months		
Inspection	0.95 -> ns	
Inspection with order	0.90 -> ns	
13-24 months		
Inspection	1.07 -> ns	
Inspection with order	0.98 -> ns	

(all not significant, 95%CI only present in figure)

Tab. 27: Results of conditional fixed effects model by inspection type, acute injuries, and match (match was performed 3 times, with a random number starting value of 320, 230, or 032)

	12 month follow-up		13-24 month follow-up	
	RR 95% CI	n	RR 95% CI	n

General	Study	Population	Exposure	Outcome	Results	Comments
					(table continued below)	

	Inspections			
Match 320	0.95 (0.86-1.05)	52,406	1.07 (0.96-1.20)	53,818
Match 230	1.00 (0.90-1.11)	52,484	0.98 (0.88-1.10)	54,797
Match 032	0.96 (0.86-1.06)	51,398	1.05 (0.94-1.17)	54,649
Unmatched	1.00 (0.93-1.08)	611,022	0.97 (0.90-1.04)	741,454
	Inspection with orders			
Match 320	0.90 (0.78-1.04)	30,640	0.98 (0.84-1.14)	35,494
Match 230	0.90 (0.78-1.04)	31,805	0.99 (0.85-1.14)	34,111
Match 032	0.89 (0.77-1.03)	30,724	0.94 (0.81-1.09)	34,332
Unmatched	0.91 (0.81-1.02)	552,235	0.96 (0.87-1.07)	671,584

Tab. 28: Results of conditional fixed effects model by inspection type, short-term, long-term and fatal (SLF) injuries, and match (match was performed 3 times, with a random number starting value of 320, 230, or 032)

	12 month follow-up		13-24 month follow-up	
	RR 95% CI	n	RR 95% CI	n
	Inspections			
Match 320	1.02 (0.95-1.10)	83,436	1.08 (1.00-1.17)	86,843
Match 230	1.05 (0.98-1.13)	84,324	1.04 (0.96-1.12)	87,798
Match 032	1.04 (0.97-1.12)	83,339	1.07 (0.99-1.16)	88,393
Unmatched	1.04 (0.99-1.10)	985,399	1.02 (0.97-1.07)	1,191,197
	Inspection with orders			
Match 320	0.99 (0.89-1.09)	50,322	1.07 (0.97-1.19)	54,952
Match 230	0.98 (0.88-1.08)	51,794	1.11 (1.00-1.23)	53,501
Match 032	0.96 (0.87-1.07)	49,279	1.05 (0.95-1.17)	53,595
Unmatched	1.01 (0.93-1.09)	899,213	1.05 (0.98-1.12)	1,086,450

General	Study	Population	Exposure	Outcome	Results	Comments
					Tab. 29: Matched and unmatched results of the conditional fixed effects for inspections with fall protection orders, fall from height injuries only	

	12 month follow-up RR 95% CI	13-24 month followup RR 95% CI
Match 320	1.07 (0.85-1.35)	0.94 (0.74-1.20)
Match 230	1.07 (0.85-1.34)	0.95 (0.73-1.13)
Match 032	1.04 (0.83-1.30)	0.97 (0.76-1.25)
Unmatched	1.13 (0.95-1.34)	0.99 (0.84-1.18)

->inspection not statistically significant associated with lower injury rate

Fig. 13-14: Inspection effect on acute injuries by sector:

Sector	12 months RR	13-24 months RR
,		
Primary resources	1.19	1.44
Manufacturing	1.13	1.05
Construction	0.96	1.07
Transportation & Warehousing	1.01	1.12
Trade	0.93	0.84
Services	0.77 (significant)	1.12

Fig. 15-16: Inspection with order effect on acute injuries by sector:

Sector	12 months RR	13-24 months RR
,		
Primary resources	0.91	0.72
Manufacturing	0.93	0.92
Construction	1.05	1.05
Transportation & Warehousing	1.22	1.06

General	Study	Population	Exposure	Outcome	Results	Comments

Trade	0.62	1.33
Services	0.57 (significant)	0.88

->lower injury rate after 12 months inspection/ inspection with order for service sector

Fig. 17-18: Inspection effect on acute injuries, by firm size

Sector	12 months RR	13-24 months RR
Inspection effect		
Small (1-5 FTE)	0.91	0.95
Medium (5-20)	1.10	1.11
Large (20+ FTE)	0.80	1.05
Inspection with order effect		
Small (1-5 FTE)	0.77	1.04
Medium (5-20)	1.03	0.95
Large (20+ FTE)	0.79	0.89

Sensitivity analysis

Tab. 30: Inspections in large service sector and non-service sector firms

	Acute injury service firms		Acute injury non-service firms	
	RR 95% CI	n	RR 95% CI	n
	12 month follow-up			
Match 320	0.64 (0.46-0.88)	3,914	0.89 (0.69-1.16)	5,234
Match 230	0.62 (0.44-0.88)	3,764	0.86 (0.66-1.129	5,155
Match 032	0.58 (0.41-0.81)	3,737	0.91 (0.70-1.18)	5,208
Unmatched	0.85 (0.68-1.06)	55,418	"model did not converge"	

->in large firms from service sector significantly lower injury rates 12 months after inspection

General	Study	Population	Exposure	Outcome	Results	Comments

Tab. 31: Inspections with site visit compared to all inspections, 12- and 13-24-months follow-up

	Acute, Inspections with site visit (2007 onwards)		Acute, inspections (2007 onwards)	
	RR 95% CI	n	RR 95% CI	n
	12-month follow-up			
Match 320	0.97 (0.86-1.09)	37,540	0.93 (0.83-1.05)	40,274
Match 230	0.97 (0.86-1.09)	38,382	1.00 (0.89-1.12)	40,513
Match 032	1.01 (0.93-1.10)	428,725	0.94 (0.83-1.06)	39,619
Unmatched	1.01 (0.93-1.10)	428,725	0.99 (0.91-1.08)	437,356
	13-24-month follow-up			
Match 320	0.99 (0.87-1.13)	38,650	0.98 (0.88-1.10)	54,797
Match 230	0.99 (0.87-1.13)	39,629	1.07 (0.96-1.20)	53,818
Match 032	1.00 (0.87-1.14)	39,531	1.05 (0.94,1.17)	54,649
Unmatched	0.94 (0.87-1.02)	494,165	0.97 (0.90-1.04)	741,454

->similar results for inspections with site visit

-another sensitivity analysis with excluding firms with outlying size and injury rates (greater than 0.1 acute injuries/FTE) from cohort
Tab. 32+33: excluding firms with outlying size

	Inspections		Inspections with orders	
	RR 95% CI	n	RR 95% CI	n
	Very small firms removed (<1st percentile FTE)			
Match 320	0.95 (0.86-1.05)	52,109	0.90 (0.78-1.04)	30,536
Match 230	1.00 (0.90-1.10)	52,259	0.90 (0.78-1.04)	31,721
Match 032	0.96 (0.86-1.06)	50,990	0.89 (0.77-1.03)	30,544
Unmatched	0.96 (0.86-1.06)	607,749	0.91 (0.81-1.02)	549,198
Very large firms removed (>99th percentile FTE)				
Match 320	0.98 (0.88-1.09)	51,153	0.91 (0.79-1.05)	30,032
Match 230	1.02 (0.92-1.13)	51,200	0.90 (0.78-1.04)	31,121

<table>
<tr><th>General</th><th>Study</th><th>Population</th><th>Exposure</th><th>Outcome</th><th>Results</th><th>Comments</th></tr>
<tr><td></td><td></td><td></td><td></td><td></td><td>
<table>
<tr><td>Match 032</td><td>0.98 (0.88-1.09)</td><td>50,195</td><td>0.90 (0.78-1.05)</td><td>30,169</td></tr>
<tr><td>Unmatched</td><td>1.00 (0.92-1.08)</td><td>590,643</td><td>0.94 (0.84-1.06)</td><td>534,261</td></tr>
</table>

Tab. 34: excluding firms with higher injury rates (>0.10 acute injuries per FTE)
<table>
<tr><td></td><td>Inspections</td><td></td><td>Inspections with orders</td><td></td></tr>
<tr><td></td><td>RR 95% CI</td><td>n</td><td>RR 95% CI</td><td>n</td></tr>
<tr><td>Match 320</td><td>0.84 (0.74-0.97)</td><td>31,790</td><td>0.81 (0.67-0.98)</td><td>19,628</td></tr>
<tr><td>Match 230</td><td>0.82 (0.71-0.94)</td><td>32,161</td><td>0.79 (0.65-0.96)</td><td>19,287</td></tr>
<tr><td>Match 032</td><td>0.81 (0.71-0.93)</td><td>31,536</td><td>0.77 (0.64-0.93)</td><td>19,984</td></tr>
<tr><td>Unmatched</td><td>0.79 (0.70-0.89)</td><td>516,660</td><td>0.76 (0.66-0.87)</td><td>562,313</td></tr>
</table>

->removing firms with (>0.10 injuries per FTE): in analysis cohort: statistically significant 16% reduction of injuries for inspections and 19% for inspections with orders

Short summary of findings:
-´Comparison of different methods for analysis:
(1 method: increase in injuries, 1 method: reduction in injuries, and two methods: no effect on injuries):
a) Haviland: decrease in injuries -> looking only at change in injuries within a firm -> firm size not considered
b) Difference/Scholz-Gray method: based on assumption that injury counts are log-normally distributed: useful when study group is homogenous but matching or another technique necessary when firm size or industry vary within the group; firm size not accounted for; rarely used
c) difference-in-differences (DiD): controls for that injury rates change over time due to reasons independent of the intervening prevention activities (but assumes that rate of change is the same in treatment and control)
d) GEE method by Foley et al.: not appropriate (selection bias created by targeted inspections not considered)
e) Levine: variability between results of each of matches
</td><td></td></tr>
</table>

<table>
<tr><th>General</th><th>Study</th><th>Population</th><th>Exposure</th><th>Outcome</th><th>Results</th><th>Comments</th></tr>
<tr><td></td><td></td><td></td><td></td><td></td><td>-> best methods: conditional fixed effects and difference-indifferences
-> accounting for unobserved covariates, combined with matching to reduce bias with similar treatment and control groups
->fixed effect conditions out unobserved covariates on an individual level, while DID conditions out differences at a group effect level
Effect of regulatory inspections on workplace injury rates in British Columbia (chapter 5)
- effect of inspections (and inspections with orders) for (large) service sector only for 12-month follow-up
- exclusion of outliers: statistically significant 16% reduction of injuries for inspections and 19% for inspections with orders for 12-month follow-up</td><td></td></tr>
<tr><td>First Author, Year
Macpherson, 2022 #reference list

Extracted by: MS

Checked by: UBA</td><td>Study name:-

Country: Canada

Study design: difference-in-differences (DID) (=controlled before-after)

Time of Study: 2009-2020</td><td>Recruitment method/ Data source:
Claim level data from Workplace Safety and Insurance Board (WSIB),
COR data from Infrastructure Health & Safety Association (IHSA)
WSIB data: included firm and claim-level data for 2009-2020 IHSA data included COR registration, COR audit-level, element-level & sub-element data extracted from Calvin database -> data linkage

Study cohort 2: COR firms with WSIB accounts with at least 1 FTE and positive insurable earnings for the year of their passed external audit, and up to a maximum of 3 years per external audit, for the years 2012 and 2020</td><td>Description of intervention:
COR program (workplace inspection is part of COR)

Study groups:
first became COR-certified between 2012 and 2019
control: non-COR firms

Time of measurement: 2009-2020</td><td>Outcome name:
Work injury (Claim)

Outcome definition and assessment:
“Claims from a work-related injury/disease which results in: being off work past the day of accident, loss of wages/earnings, or a permanent disability/impairment”
- separate work-related injury claim rates were analysed: 1) lost time claims injury = claims from a work-related injury/disease after an accident</td><td>Descriptive Statistics:
Workplace inspection: sub-elements: n=11, possible score: 31, %Total score: 7.0
% of firm audits scoring 100% for workplace inspections: 54%

Tab.3: Summary of firms by cohort restriction for study cohort 2
<table>
<tr><td></td><td>COR</td></tr>
<tr><td>Firms with passed external audits (2012-2020)</td><td>526</td></tr>
<tr><td>Firms with complete audit, element and sub-element data*</td><td>479</td></tr>
<tr><td>Firms linked to WSIB data extract</td><td>448</td></tr>
<tr><td>Firms with 1+ FTEs and insurable earnings for at least one year**</td><td>442 (606 audits)</td></tr>
</table>
* “Firms with COR equivalency certifications are automatically assigned 100% in the Calvin database. These firms and others with missing audit element and sub-element data were removed. 1 firm transitioned from the “Small business (2-4 workers) audit” to the “Standard (20+ worker)” audit. ** Firms required at least one year of 1+ FTEs up to a maximum of three years.”

Statistical methods used: difference-in-differences (DiD) design (2 categories score percentages: 100% vs <100%), negative binomial models, incidence rate ratio (IRR)

Unadjusted/ adjusted estimates with precision (e.g. 95% confidence interval) for each outcome

Tab. 5: Negative binomial regression
<table>
<tr><td></td><td>Lost time</td><td>High-impact</td><td>No lost time</td></tr>
<tr><td></td><td>IRR (95% CI)</td><td>IRR (95% CI)</td><td>IRR (95% CI)</td></tr>
</table></td><td>Funding:
Infrastructure Health and Safety Association (IHSA), Michael Smith Foundation for Health Research Scholar Award

Conflict of Interest stated: “All inferences, opinions, and conclusions drawn within this publication are those of the authors, and do not reflect the opinions or policies of the data stewards.“

Other comments:
- ethics approval University of British Columbia Behavioural Research Ethics</td></tr>
</table>

<table>
<tr><th>General</th><th>Study</th><th>Population</th><th>Exposure</th><th>Outcome</th><th>Results</th><th>Comments</th></tr>
<tr><td></td><td></td><td>Matching criteria: balance COR and non-COR firms on observable characteristics, Coarsened Exact Matching (CEM): industry classification unit, firm size, year, and pre-baseline lost time injury rate (lagged three-year average) Even after matching, participation rate in occupational safety and health programs of the Workplace Safety and Insurance Board was higher in the COR group compared to the control group (22.5 versus 9.7%).

invited companies: NA

companies at baseline: N=526

Response: NA

companies at follow –up: NA

Loss-to-follow-up: NA

Work characteristics: no restriction</td><td></td><td>which results in: being off work past the day of accident, loss of wages/earnings, or a permanent disability/impairment
2) high-impact claims = lost time claims that have large health and financial impacts on employees and businesses: low back, shoulder, and fracture claims and 3) no lost time claims = claims from work-related injury/disease where no time is lost from work, other than on the day of accident, but where health care is required

Time of measurement: 2009-2020</td><td>
<table>
<tr><td>Baseline</td><td>0,88 (0,75-1,03)</td><td>0,87 (0,74-1,03)</td><td>1,07 (0,94-1,22)</td></tr>
<tr><td>Intervention</td><td>0,72 (0,63-0,84)</td><td>0,80 (0,66-0,97)</td><td>1,00 (0,92-1,08)</td></tr>
<tr><td>Firm years</td><td>7,255</td><td>7,255</td><td>7,255</td></tr>
<tr><td>Firms</td><td>656</td><td>656</td><td>656</td></tr>
</table>
IRR = incidence rate ratio. CI = confidence interval. Estimates from fully adjusted, population-averaged models with offsets of logged FTEs, including non-COR firms and COR firms

Tab. Appendix 12: Low variation elements: element scores and lost time injuries
(incidence rate ratios (IRR) and 95% confidence intervals (CI, reference = 100%); population-averaged models with offsets of logged FTEs, adjusting for NAICS class/subclass, firm size, firm years, premium rate adjustment, year, OHS program participation, employer province, audit type. Separate models for each element. 442 firms (1,391 firm years).
Workplace inspections: IRR=1.20 95% CI 0.97-1.60, %audits <100:21.0, %Total score: 7.0
<u>Only construction sector:</u>
Workplace inspections: IRR=1.30 95% CI 1.02-1.65, %audits <100:47.4, %Total score: 7.0

Short summary of findings:
- 20%higher lost claim rates for firms with less than 100% on workplace inspections (respectively 30% when only construction sector was analysed)
- Firms with COR have significant lower lost time and High impact claims.</td><td>Board (certificate number H21-02492)
- study focuses on intervention of Certificate of Recognition (COR)
- COR=audit-based certificate program offered by occupational health and safety (OHS) organisations across Canada</td></tr>
<tr><td>First Author, Year
Macpherson, 2021
#2818</td><td>Study name:-

Country: Canada</td><td>Recruitment method/ Data source:
Data from Inspection Experience and Impact (IEI): bi-annual telephone survey conducted by Ipsos</td><td>Description of intervention: workplace safety inspections</td><td>Outcome name: hazard management</td><td>Descriptive Statistics:
-inspections changed how hazards were managed: 70% respondents some form of change: 29.0% minor changes, 32.0% moderate changes and 9.4% major changes</td><td>Funding:
WorkSafeBC, Michael Smith Health Research Foundation Scholar Award</td></tr>
</table>

<table>
<tr><th>General</th><th>Study</th><th>Population</th><th>Exposure</th><th>Outcome</th><th>Results</th><th>Comments</th></tr>
<tr>
<td>
Extracted by:

Checked by:</td>
<td>**Study design:** Cross-sectional

Time of Study: 2018-2019</td>
<td>(independent market research company) on behalf of WorkSafeBC
-survey randomized from all inspection reports submitted to WorkSafeBC in previous 6 months (exclusing inspections that resulted in penalty and firms with fatalities)
-4th quarter of 2018 survey (n=722) and 1rst and 2nd quarter of 2019 (n=1,077)

Matching criteria: NA

 # invited companies: NR

 # companies at baseline: n=1,799
(n=1,578 (87.7%) with valid answers of the full sample)

Response: NR

 # companies at follow –up: NA

Loss-to-follow-up: NA

Work characteristics:
Fig. 3: Distribution of companies
<table>
<tr><th>*Sector*</th><th>*n*</th></tr>
<tr><td>Primary resources</td><td>99</td></tr>
<tr><td>Transportation & ware-housing</td><td>46</td></tr>
<tr><td>Construction</td><td>628</td></tr>
</table>
</td>
<td>

Study groups: NA

Time of measurement: 2018-2018</td>
<td>**Outcome definition and assessment:**
“Did the inspection lead to changes in how hazards are managed at your workplace?” Responses could include no changes, minor changes, moderate changes, or major changes

Time of measurement: 2018-2018</td>
<td>- minor or moderate changes more likely to be reported when officers rated as being very good for explaining ways to be manage hazards or explaining changes required be compliant with regulation
-response inspections associated with a smaller proportion of no hazard management changes (26.8% versus 32.0%), and higher proportions of moderate and major changes (35.9% and 11.0% versus 28.6% and 7.9%) compared to planned inspections
- Inspections did not include orders no changes reported by higher proportion of respondents (41.1%), followed by initiating orders (23.4%) and order follow-ups (15.8%). Similar results for moderate and major changes.
- difference of more than 10 percentage points between respondents who reported not having had a post-inspection discussion with inspection officer and reporting no changes in how hazards were managed at their workplaces (37.3% versus 26.2%). Having had a post-inspection discussion more likely to report all three types of changes.

Fig.3: Inspection characteristics
<table>
<tr><th>Characteristic</th><th>n</th></tr>
<tr><td>*Inspection type*</td><td></td></tr>
<tr><td>Planned inspection</td><td>843</td></tr>
<tr><td>Response inspection</td><td>735</td></tr>
<tr><td>*Type of order issued*</td><td></td></tr>
<tr><td>No orders</td><td>655</td></tr>
<tr><td>Initiating orders</td><td>689</td></tr>
<tr><td>Order follow-up</td><td>234</td></tr>
</table>

App A: Change in how hazards were managed as result of inspection
<table>
<tr><th>Changes</th><th>N</th></tr>
<tr><td>No changes</td><td>467</td></tr>
<tr><td>Minor changes</td><td>458</td></tr>
<tr><td>Moderate changes</td><td>505</td></tr>
<tr><td>Major changes</td><td>148</td></tr>
</table>

Statistical methods used: unadjusted and adjusted multinomial logistic regression models (Odds ratios (OR) 95% CI)

Unadjusted/ adjusted estimates with precision (e.g. 95% confidence interval) for each outcome:</td>
<td>**Conflict of Interest stated:** yes

Other comments: -</td>
</tr>
</table>

General	Study	Population	Exposure	Outcome	Results	Comments
		Public sector 44; Service sector 359; Manufacturing 252; Trade 150			see below	

Public sector	44	
Service sector	359	
Manufacturing	252	
Trade	150	

Tab. 1: Multivariable multinomial regression estimating the likelihood of respondents reporting changes in how hazards were managed at their workplaces as a result of inspections (n = 1,578).

	Minor changes OR 95% CI	Moderate changes OR 95% CI	Major changes OR 95% CI
Inspection type			
Planned (ref)	1	1	1
Response	1.00 (0.77-1.31)	1.59 (1.22-2.07)	1.78 (1.21-2.63)
Order type			
No order (ref)	1	1	1
Initiating order	1.87 (1.41-2.49)	2.75 (2.06-3.67)	3.16 (2.04-4.90)
Order follow-up	2.45 (1.56-3.86)	4.95 (3.20-7.66)	5.56 (3.08-10.05)
Post-inspection discussion			
No (ref)	1	1	1
Yes	1.37 (1.02-1.82)	1.53 (1.14-2.05)	1.35 (0.88-2.08)

OR=odds ratio, CI= confidence interval

(results of unadjusted model not extracted)

App D: Adjusted multinomial logistic regression models for construction sector (n=628)

	Minor changes OR 95% CI	Moderate changes OR 95% CI	Major changes OR 95% CI
Inspection type			
Planned (ref)	1	1	1
Response	1.06 (0.70-1.60)	2.10 (1.36-3.26)	3.45 (1.77-6.73)
Order type			
No order (ref)	1	1	1
Initiating order	1.42 (0.92-2.21)	1.78 (1.12-2.81)	3.13 (1.60-6.12)
Order follow-up	3.63 (1.13-11.69)	6.46 (2.05-20.34)	10.73 (2.65-43.52)
Post-inspection discussion			
No (ref)	1	1	1
Yes	1.03 (0.66-1.60)	1.79 (1.10-2.91)	1.84 (0.88-3.87)

OR=odds ratio, CI= confidence interval

App E: Adjusted multinomial logistic regression models inspections with orders

	Minor changes OR 95% CI	Moderate changes OR 95% CI	Major changes OR 95% CI
Inspection type			
Planned (ref)	1	1	1
Response	1.03 (0.70-1.50)	1.68 (1.17-2.42)	1.70 (1.04-2.78)
Order type			
Initiating order	1	1	1
Order follow-up	1.28 (0.80-2.04)	1.82 (1.17-2.82)	1.73 (0.99-3.05)

<table>
<tr><th>General</th><th>Study</th><th>Population</th><th>Exposure</th><th>Outcome</th><th>Results</th><th>Comments</th></tr>
<tr><td></td><td></td><td></td><td></td><td></td><td>
<table>
<tr><td colspan="4">Post-inspection discussion</td></tr>
<tr><td>No (ref)</td><td>1</td><td>1</td><td>1</td></tr>
<tr><td>Yes</td><td>1.47 (0.96-2.24)</td><td>1.52 (1.01-2.28)</td><td>1.37 (0.79-2.38)</td></tr>
</table>
OR=odds ratio, CI= confidence interval

App G: Adjusted multinomial logistic regression models inspections without orders
<table>
<tr><th></th><th>Minor changes OR 95% CI</th><th>Moderate changes OR 95% CI</th><th>Major changes OR 95% CI</th></tr>
<tr><td colspan="4">Inspection type</td></tr>
<tr><td>Planned (ref)</td><td>1</td><td>1</td><td>1</td></tr>
<tr><td>Response</td><td>1.16 (0.78-1.72)</td><td>1.53 (1.01-2.30)</td><td>2.55 (1.27-5.15)</td></tr>
<tr><td colspan="4">Post-inspection discussion</td></tr>
<tr><td>No (ref)</td><td>1</td><td>1</td><td>1</td></tr>
<tr><td>Yes</td><td>1.36 (0.91-2.05)</td><td>1.56 (1.00-2.43)</td><td>1.48 (0.69-3.18)</td></tr>
</table>
OR=odds ratio, CI= confidence interval

Short summary of findings:
- over 2/3 reported changes in hazard management as results of inspection (just 1/3 moderate changes, 10% major changes)
- higher odds of reporting workplace changes for response inspections, relative to planned inspections, was only observed for moderate (OR 1.59; 95% CI 1.22–2.07) and major changes (OR 1.78; 95% CI 1.21–2.63).
- effect of inspections with orders persisted for all forms of changes in how workplace hazards were managed, with highest odds for order follow-ups for minor (OR 2.45, 95% CI 1.56–3.86), moderate (OR 4.95; 95% CI 3.20–7.66), and major changes (OR 5.56; 95% CI 3.08–10.05)
- having had post-inspection discussions only had higher odds of reporting minor (OR 1.37; 95% CI 1.02–1.82) and moderate changes (OR 1.53, 95% CI 1.14–2.05) in workplace hazard management, relative to those who had not had post-inspection
- sensitivity analysis: construction only: reporting a major change in workplace hazard management was tenfold higher for order follow-ups than no orders, albeit with wide confidence intervals (OR 10.73, 95% CI 2.65–43.52)
</td><td></td></tr>
<tr><td>First Author, Year
Li, 2022
#4353</td><td>Study name: -

Country: USA</td><td>Recruitment method/ Data source:
6 Data sets from Mine Data Retrieval System (MDRS), and maintained by Mining Safety</td><td>Description of intervention: inspection data set: 1)</td><td>Outcome name: workplace injuries</td><td>Descriptive Statistics:
- Mines with flagrant violations had higher injury rates and received more inspections and penalties: average quarterly injury rate of mines with flagrant violations is 6.66 cases per 100 employees, compared to 3.38 cases in mines without flagrant violations</td><td>Funding: NR

Conflict of Interest stated: no</td></tr>
</table>

<table>
<tr><th>General</th><th>Study</th><th>Population</th><th>Exposure</th><th>Outcome</th><th>Results</th><th>Comments</th></tr>
<tr>
<td>Extracted by: MS

Checked by:</td>
<td>Study design: Cross-sectional study (event-study model)

Time of Study: 2000-2016</td>
<td>and Health Administration (MSHA): accident and injuries data set, quarterly employment/ production data set, inspection data set, violation data set, assessed violation
data set, and mine addresses of record data set

Matching criteria: NA

invited companies: NA

# companies at baseline:
N=183 coal mines
8,133 mine-quarter observations

Response: NA

companies at follow –up: NA

Loss-to-follow-up: NA

Work characteristics: coal mines only with a fatal accident within one year before the flagrant violation are excluded
- coal mine with multiple citations of flagrant violations, only the first violation is included
- observations with quarterly injury rates higher than 100 injuries per 100 fulltime</td>
<td>universe of MSHA inspections
2) violation data set & assessed violation: type of violation and the assessed amount of penalty, if any
- penalties normalized to 2010 dollar values

Study groups: NA

Time of measurement: 2000-2016</td>
<td>Outcome definition and assessment:
- quarterly injury rate=number of injuries per 100 full-time equivalent employees in a quarter
- injury rate by level of severity:
1) all
2) ”permanent”: injuries with fatalities or permanent disabilities
3) ”days away or restriced”: injuries involving days away from work or work restrictions
4) ”mild”: injuries with medical attention but no loss of workdays

- exclusion: occupational illnesses (3.8% of injuries & illnesses), injuries attributable to natural causes, injuries involving non-employees, injuries with</td>
<td>Tab.1: Descriptive statistics
<table>
<tr><th></th><th>all</th><th>Flagrant violations</th><th>No flagrant violations</th><th>No flagrant violations, closest match</th></tr>
<tr><td>Inspections</td><td>2.451 (4.266)</td><td>8.773 (9.840)</td><td>1.912 (2.793)</td><td>3.204 (5.681)</td></tr>
<tr><td>Penalties: Initial ($1,000)</td><td>6.818 (54.423)</td><td>56.633 (181.932)</td><td>2.573 (12.814)</td><td>5.497 (21.735)</td></tr>
<tr><td>Penalties: Final ($1,000)</td><td>5.293 (45.256)</td><td>42.340 (153.268)</td><td>2.135 (9.687)</td><td>4.632 (16.518)</td></tr>
<tr><td>Violations</td><td>9.317 (22,867)</td><td>46.845 (54,912)</td><td>6.119 (13,426)</td><td>11.861 (24,309)</td></tr>
<tr><td>Injury rate</td><td>3.634 (9.727)</td><td>6.663 (9.510)</td><td>3.375 (9.702)</td><td>4.935 (11.271)</td></tr>
<tr><td>Injury rate: permanent</td><td>0.044 (0.946)</td><td>0.106 (1.107)</td><td>0.038 (0.939)</td><td>0.077 (1.107)</td></tr>
<tr><td>Injury rate: Days lost</td><td>2.590 (8.084)</td><td>4.579 (7.702)</td><td>2.420 (8.093)</td><td>3.462 (9.605)</td></tr>
<tr><td>Injury rate: mild</td><td>1.000 (4.734)</td><td>1.978 (4.579)</td><td>0.917 (4.738)</td><td>1.396 (5.334)</td></tr>
<tr><td>Employment hours (1,000)</td><td>22.899 (49.334)</td><td>94.594 (113.285)</td><td>16.788 (32.744)</td><td>31.723 (68.570)</td></tr>
<tr><td>Quarterly hours per worker</td><td>519 (182)</td><td>562 (129)</td><td>515 (185)</td><td>558 (151)</td></tr>
</table>
Statistical methods used: regression (not described)

Unadjusted/ adjusted estimates with precision (e.g. 95% confidence interval) for each outcome:

Tab.2: Effect of flagrant violations on workplace safety (N=8,133)
<table>
<tr><th></th><th>Injury rate Coef. (SE)</th><th>Injury rate: permanent Coef. (SE)</th><th>Injury rate: days away or restricted Coef. (SE)</th><th>Injury rate: mild Coef. (SE)</th></tr>
<tr><td>Years 1 to 2</td><td>-0.182 (0.535)</td><td>0.030 (0.031)</td><td>-0.542 (0.439)</td><td>0.331 (0.256)</td></tr>
<tr><td>Years 3 to 4</td><td>-1.388** (0.487)</td><td>-0.018 (0.024)</td><td>-1.400*** (0.400)</td><td>0.029 (0.228)</td></tr>
<tr><td>Years 5 and after</td><td>-1.440** (0.717)</td><td>0.054 (0.040)</td><td>-1.391** (0.573)</td><td>-0.103 (0.329)</td></tr>
<tr><td>R^2</td><td>0.058</td><td>0.013</td><td>0.051</td><td>0.022</td></tr>
</table>
The model includes mine fixed effects and calendar quarter fixed effects. Standard errors (shown in parentheses) are clustered at the mine level. ***, **, and * indicate statistical significance at the 1, 5, and 10% levels, respectively.</td>
<td>Other comments:
- also investigated productivity</td>
</tr>
</table>

<table>
<tr><th>General</th><th>Study</th><th>Population</th><th>Exposure</th><th>Outcome</th><th>Results</th><th>Comments</th></tr>
<tr><td></td><td></td><td>equivalent employees are excluded (0.5%)</td><td></td><td>missing classification code

Time of measurement: 2000-2016</td><td>Short summary of findings:
- injury rate decreased by 0.18 cases per 100 employees during first two years after a flagrant violation (representing a 3% decrease compared to average total case rate of 7.0 in the quarter right before flagrant violation)
- between year three and year four after flagrant violation, injury rate decreased by 1.39 cases per 100 employees (20%); rate decreased by 1.44 cases (21%) at year five and later level of severity
- decrease in workplace injuries primarily from decrease in injuries with days away or restricted from work -> dropped by 0.54 cases during the first two years after flagrant violation and by 1.40 cases between year 3 & 4.
- fatal and permanent injuries and mild injuries ->small and statistically insignificant changes before and after the flagrant violation (possibly due to decrease in working hours after flagrant violation)</td><td></td></tr>
<tr><td>First Author, Year
Li, 2019
#4365

Extracted by: MS

Checked by:</td><td>Study name: -

Country: USA

Study design: (Fuzzy Regression Discontinuity) (quasi-experimental pre & post test design)</td><td>Recruitment method/ Data source:
Data from OSHA Data Initiative (ODI): company data, accidents and injuries
OSHA IMIS: inspection data (programmed/ unprogrammed inspections, citations and penalties)
Data from Federal Mediation and Conciliation Service

ODI: pooled data 1996-2011: 1,018,600 establishment-by-year -> observations dropped if duplicate record or company name & address missing -> stacking observations based on companies name & address -> 341,302 unique companies</td><td>Description of intervention:
OSHA's Site-Specific Targeting (SST) plan: program targeted companies for inspection if their baseline case rate exceeded a cutoff (case-rate cutoff) increase in programmed inspections</td><td>Outcome name:
a)TCR=Rate of cases involving death, days away from work, job transfers and restrictions, and medical attention beyond first aid
b)DART= cases involving days away from work and job transfers and restrictions
c) DAFWII includes cases involving days away from work

Outcome definition and</td><td>Descriptive Statistics:

Tab.1: Summary statistics (mean + SE)
<table>
<tr><th></th><th>all</th><th>DART < cutoff</th><th>DART ≥ cutoff</th></tr>
<tr><td>TCR t</td><td>12.81 (0.03)</td><td>10.49 (0.02)</td><td>26.96 (0.08)</td></tr>
<tr><td>DART t</td><td>7.33 (0.02)</td><td>5.41 (0.01)</td><td>19.06 (0.05)</td></tr>
<tr><td>Cutoff</td><td>13.67 (0.01)</td><td>13.71 (0.01)</td><td>13.41 (0.02)</td></tr>
<tr><td>Inspection, programmed</td><td>0.086 (0.001)</td><td>0.051 (0.001)</td><td>0.303 (0.003)</td></tr>
<tr><td>Citation, programmed</td><td>0.064 (0.001)</td><td>0.036 (0.001)</td><td>0.230 (0.003)</td></tr>
<tr><td>Penalty, programmed</td><td>0.057 (0.001)</td><td>0.033 (0.000)</td><td>0.203 (0.003)</td></tr>
<tr><td>Inspection, unprogrammed</td><td>0.046 (0.001)</td><td>0.044 (0.001)</td><td>0.062 (0.002)</td></tr>
<tr><td>Manufacturing</td><td>0.610 (0.001)</td><td>0.622 (0.001)</td><td>0.536 (0.003)</td></tr>
<tr><td>Health services</td><td>0.175 (0.001)</td><td>0.177 (0.001)</td><td>0.166 (0.003)</td></tr>
<tr><td>Union activity</td><td>0.125 (0.001)</td><td>0.122 (0.001)</td><td>0.143 (0.002)</td></tr>
<tr><td>TCR t+4</td><td>9.51 (0.02)</td><td>8.71 (0.02)</td><td>14.38 (0.06)</td></tr>
<tr><td>DART t+4</td><td>5.69 (0.01)</td><td>5.08 (0.01)</td><td>9.47 (0.05)</td></tr>
<tr><td>Companies</td><td>61,702</td><td>55,247</td><td>6,455</td></tr>
<tr><td>Observations</td><td>154,808</td><td>133,013</td><td></td></tr>
</table>
t =first observation, t+4=second observation; cutoff= DART rate cutoff for the primary inspection list</td><td>Funding: NR

Conflict of Interest stated: no

Other comments: -</td></tr>
</table>

<table>
<tr><th>General</th><th>Study</th><th>Population</th><th>Exposure</th><th>Outcome</th><th>Results</th><th>Comments</th></tr>
<tr>
<td></td>
<td>Time of Study: 1996-2011</td>
<td>of which 188,178 -> 1 observation -> aim: paired observations per company limiting data to pairs of observations being 4 years apart

Matching criteria: NA

invited companies: NA

companies at baseline: n=252,382 paired observations for which first years is in 1996 to 2007.
Exclusion of data pairs:
a) First observation was in 1996 (data not implemented in SST plan)
b) states that do not participate in SST plan (->35 states included)
c) missing ODI case rate or case rate exceeds 100

n=154,808 observation pairs among 61,702 unique establishments (average of 2.5 observation pairs per establishment)

Response: NA

companies at follow –up: NA

Loss-to-follow-up: NA</td>
<td>at the cutoff is used to identify the effect of an inspection on worker safety

Study groups: NA

Time of measurement: 1996-2011</td>
<td>assessment: Case rates =per 100 fulltime equivalent workers

Time of measurement: 1996-2011</td>
<td>- discontinuity in inspections at the cutoff: -> cutoff is associated with a 22.7 percentage point increase in programmed inspections, a 17.6 percentage point increase in citations, and a 15.7 percentage point increase in penalties
- programmed inspection increased the number of citations by 5.06 (no discontinuous change on unprogrammed inspections at cutoff)

Statistical methods used: regression discontinuity model using local linear regression, with the case rate as the outcome variable and a programmed inspection as the treatment variable. Covariates: year fixed effects, state fixed effects, industry fixed effects (manufacturing, health services, and other), and an indicator of union activity; fuzzy regression discontinuity=FRD -> for discontinuity in inspections to identify the causal effect of inspections

Unadjusted/ adjusted estimates with precision (e.g. 95% confidence interval) for each outcome:

Tab.4: Effect of Inspection on Worker Safety (adjusted model, mean effects with standard error)
<table>
<tr><td>ODI year</td><td colspan="2">1997-2007</td><td colspan="3">1998-2007</td></tr>
<tr><td></td><td>TCR</td><td>DART</td><td>TCR</td><td>DART</td><td>DAFWII</td></tr>
<tr><td>τFRD</td><td>-0.769
(0.150)</td><td>-1.792**
(0.814)</td><td>-1.717
(1.215)</td><td>-2,068**
(0.872)</td><td>-0.554
(0.607)</td></tr>
<tr><td>αDR - αDL</td><td>0.224***
(0.013)</td><td>0.224***
(0.013)</td><td>0.215***
(0.013)</td><td>0.215***
(0.013)</td><td>0.215***
(0.013)</td></tr>
<tr><td>Bandwidth h</td><td>3.45</td><td>3.17</td><td>3.25</td><td>3.13</td><td>3.57</td></tr>
<tr><td>Observations</td><td>154,808</td><td>154,808</td><td>139,220</td><td>139,220</td><td>139,220</td></tr>
</table>
Covariates=year fixed effects, state fixed effects, industry fixed effects (manufacturing, health services, and other), and an indicator of union activity
αDR= mean outcome just above the Site-Specific Targeting (SST) cutoff
αDL=outcome just below the SST cutoff
τFRD= fuzzy regression discontinuity
***, **, and * statistical significance at the 1%, 5%, and 10% levels

Tab.7: Effect of Inspection on Worker Safety by Industry (adjusted model, mean effects with standard error)
<table>
<tr><td></td><td colspan="3">Industry</td></tr>
<tr><td></td><td>Manufacturing</td><td>Health service</td><td>Other</td></tr>
<tr><td>τFRD</td><td>-1.050 (0.859)</td><td>0,626 (1.317)</td><td>-0.124 (1.532)</td></tr>
<tr><td>αDR - αDL</td><td>0.208*** (0.013)</td><td>0.238*** (0.022)</td><td>0.245*** (0.024)</td></tr>
</table>
</td>
<td></td>
</tr>
</table>

<table>
<tr><th>General</th><th>Study</th><th>Population</th><th>Exposure</th><th>Outcome</th><th>Results</th><th>Comments</th></tr>
<tr><td></td><td></td><td>Work characteristics: registry of businesses in the United States: ODI targeted establishments with minimum number of employees in manufacturing and other industries with injury rates above national average, excluding construction</td><td></td><td></td><td>
<table>
<tr><td>Bandwidth h</td><td>6.37</td><td>6.11</td><td>3.07</td></tr>
<tr><td>Observations</td><td>94,410</td><td>27,136</td><td>33,262</td></tr>
</table>
Covariates=year fixed effects, state fixed effects, industry fixed effects (manufacturing, health services, and other), and an indicator of union activity
αDR= mean outcome just above the Site-Specific Targeting (SST) cutoff
αDL=outcome just below the SST cutoff
τFRD= fuzzy regression discontinuity
***, **, and * statistical significance at the 1%, 5%, and 10% levels

- authors also used alternative samples:
a) longer lags between 1rst and 2nd (5 and 6 years)-> longer lag decrease sample size-> smaller estimates-> positive insignificant-> inspection on worker safety may be ephemeral
b) companies less able to anticipate SST plan and DART cutoff: estimates remain negative but statistically not significant

Short summary of findings:
- inspection decreases DART (TCR not significant)
- inspection mechanism decrease DART rate more than TCR and DAFWII
- average effect of an inspection on DART rate is –1.792 per 100 full-time equivalent workers—a reduction of 20% relative to the post-inspection DART rate near the cutoff (mainly for most evident for manufacturing establishments below the 90th percentile of the DART rate distribution post-inspection
- TCR: -0.769
- effect of industry: Manufacturing -1.050 per 100 full-time equivalent workers, compared to 0.626 for health services and – 0.124 for ‘‘other’’ industries (all not significant)
</td><td></td></tr>
<tr><td>First Author, Year
Li, 2018
#4325

Extracted by: MS</td><td>Study name: -

Country: USA

Study design: (Fuzzy Regression</td><td>Recruitment method/ Data source:
OSHA Data Initiative (ODI): work safety data; companies with ≥40 employee sampled annually from 1996 to 2011: 143,771 surveys on 23,917 nursing facilities
Integrated Management Information System (IMIS) data: inspection data</td><td>Description of intervention: effect of the SST plan on the frequency of inspections
-SST plan prioritized nursing</td><td>Outcome name: Total Case Rate (TCR) and Days Away, Restricted, and Transfer (DART) case rate

Outcome definition and assessment:</td><td>Descriptive Statistics:
Tab.2: Summary Statistics
<table>
<tr><th></th><th>Mean (SD)</th></tr>
<tr><td>Injury case rate</td><td></td></tr>
<tr><td>TCR</td><td>10.566 (7.409)</td></tr>
<tr><td>DART</td><td>6.868 (5.197)</td></tr>
<tr><td>Inspections</td><td></td></tr>
<tr><td>Inspections</td><td>0.041 (0.199)</td></tr>
<tr><td>Violations</td><td>0.026 (0.159)</td></tr>
<tr><td>N</td><td>11,832</td></tr>
</table>
</td><td>Funding: NR

Conflict of Interest stated: no

Other comments:
- database result refers to a publication in 2023 with same title:</td></tr>
</table>

<table>
<tr><th>General</th><th>Study</th><th>Population</th><th>Exposure</th><th>Outcome</th><th>Results</th><th>Comments</th></tr>
<tr>
<td>Checked by:</td>
<td>Discontinuity) (quasi-experimental pre & post test design)

Time of Study: 2004-2011</td>
<td>ODI/IMIS data matched to census of nursing facilities complied by Centers for Medicare & Medicaid Services (CMS); records of nursing facilities derived from Online Survey, Certification and Reporting (OSCAR) database
OSCAR: data on operational characteristics, resident health outcomes, staffing level, and records on deficiency citations issued by state health agencies; data collected annually; data available from 2006-2011
-> main sample nursing facilities survey by ODI from 2002-2007

Matching criteria: NA

invited companies: NA

companies at baseline: N=11,832 nursing facility-year observations
->subsample for inspection effect: only facilities with survey four year after initial survey: n=4,707

Response: NA

companies at follow –up: NA</td>
<td>facilities for inspection if the DART case rates exceeded the SST threshold

Study groups: NA

Time of measurement: SST plan from mid 2004 to mid 2010</td>
<td>Measured around 1 year after end of SST inspection cycle

Time of measurement: 2006-2011</td>
<td>- frequency of inspections year before and after SST inspection cycle is relatively low and shows no discontinuous change at the SST threshold
- nursing facilities with DART above the SST threshold are more likely to be inspected
- DART and TCR one year after the SST plan are positively correlated with DART in the initial survey year, both measures show a discontinuous decrease right at the SST threshold -> inspections are associated with lower injury case rates

Statistical methods used: regression discontinuity design

Unadjusted/ adjusted estimates with precision (e.g. 95% confidence interval) for each outcome:
Tab.4: Effect of Inspection on Injury rate one year after SST plan
<table>
<tr><th></th><th>Mean at SSt</th><th>Reduced Form</th><th>Two-Stage</th></tr>
<tr><td>DART</td><td>14.452</td><td>-1.298* (0.821)</td><td>-5.599*(2.968)</td></tr>
<tr><td>TCR</td><td>19.348</td><td>-2.061* (1.150)</td><td>-7.298* (4.355)</td></tr>
<tr><td>N</td><td>4,707</td><td>4,707</td><td>4,707</td></tr>
</table>
Column 2 = mean of the outcome at SST threshold.
Column 3 & 4 = estimate from local linear models with a triangular kernel, the optimal bandwidth & robust standard errors clustered at facility level
All the models include controls on the number of beds, whether in a chain, whether for profit, share of Medicaid patients, ADL acuity index, and state and year fixed effects. * p<0.10, ** p<0.05, *** p<0.01
Reduced form: measure the size of the discontinuity at the SST threshold.
two-stage: SST threshold as an instrument of the inspection variable

Short summary of findings:
- After an inspection, DART decreases by 5.6 cases per 100 employers, representing a 38 percent decrease among nursing facilities close to the SST threshold. TCR case rate decreases by 7.3 cases per 100 employees (38 percent). Both DART and TCR decrease by a similar proportion, suggesting that inspections reduce both mild injuries with no losses of workdays and relatively severe injuries with losses of workdays</td>
<td>https://www.journals.uchicago.edu/doi/abs/10.1086/722606
-study also investigates healthcare quality & productivity: inspections lead to worse healthcare quality and lower nurse productivity</td>
</tr>
</table>

<table>
<tr><th>General</th><th>Study</th><th>Population</th><th>Exposure</th><th>Outcome</th><th>Results</th><th>Comments</th></tr>
<tr><td></td><td></td><td>Loss-to-follow-up: NA

Work characteristics: nursing facilities</td><td></td><td></td><td></td><td></td></tr>
<tr><td>First Author, Year
Mendeloff, 2021 #3926

Extracted by: MS

Checked by:</td><td>Study name:

Country: USA

Study design: quasi-experimental

Time of Study: 1992-2012</td><td>Recruitment method/ Data source:
29 “federal OSHA” states. Data of inspections & violations from Department of Labour enforcement data webpage.
Sample definition: standards cited at least 1,000 times -> resulted in a total of 91 standards -> for this sample: all inspections for serious, wilful, or repeat (SWR) violations -> creation of a separate “initial citation” observation for each plant & standard violated -> linking to inspection data

Matching criteria: NA

invited companies: NA

companies at baseline: N=181,040 paired inspection-standard observations (based on 84,827 initial citation records from 23,499 inspections)

Response: NA

companies at follow –up: NA</td><td>Description of intervention: inspection

- programmed safety inspections
- programmed health inspections
- complaint safety inspections
- complaint health inspections
- safety follow-up inspections
- health follow-up inspections

Study groups: NA

Time of measurement: initial violations: 1992 to 2002 -> follow-up to 2012</td><td>Outcome name: re-violation

Outcome definition and assessment: Linking initial citations to data from all programmed, complaint, and follow-up inspections that occurred through 10 years following the initial inspection

Time of measurement: initial violations: 1992 to 2002 -> follow-up to 2012</td><td>Descriptive Statistics:
Tab.4: Percentage of following inspections with re-violations of the standard
<table>
<tr><th></th><th>Mean (%)</th><th>Median (%)</th></tr>
<tr><td>All inspection types, all standards</td><td>4.8</td><td>4.0</td></tr>
<tr><td>Only programmed inspections, all standards</td><td>6.2</td><td>5.0</td></tr>
<tr><td>All inspection types, matched to S/H standards</td><td>6.8</td><td>5.5</td></tr>
<tr><td>Programmed inspections, matched to S/H standards</td><td>9.5</td><td>7.6</td></tr>
<tr><td>Programmed safety inspections, safety standards</td><td>9.4</td><td>7.9</td></tr>
<tr><td>Programmed health inspections, health standards</td><td>9.7</td><td>7.5</td></tr>
<tr><td>Complaint safety inspections, safety standards</td><td>6.0</td><td>4.7</td></tr>
<tr><td>Complaint health inspections, health standards</td><td>5.6</td><td>5.0</td></tr>
<tr><td>Follow-up safety inspections, safety standards</td><td>3.1</td><td>2.4</td></tr>
<tr><td>Follow-up health inspections, health standards</td><td>4.7</td><td>4.2</td></tr>
</table>

Statistical methods used: logistic regression

Unadjusted/ adjusted estimates with precision (e.g. 95% confidence interval) for each outcome:

Tab.7: Likelihood of re-violation, given an inspection, federal occupational safety and health administration manufacturing (logistic regression – marginal effects)
<table>
<tr><th></th><th>All inspec</th><th>Safety progr</th><th>Health progr</th><th>Safety compl</th><th>Health compl</th><th>Safety follow</th><th>Health follow</th></tr>
<tr><td>Mean re-violation</td><td>6.93%</td><td>11.75 %</td><td>5.91%</td><td>7.11%</td><td>3.73%</td><td>3.83%</td><td>4.01%</td></tr>
<tr><td colspan="8">Laster inspection type (base=HC)</td></tr>
<tr><td>Health follow-up</td><td>0.001 (0.17)</td><td></td><td></td><td></td><td></td><td></td><td></td></tr>
<tr><td>Health programmed</td><td>0.025*** (6.95)</td><td></td><td></td><td></td><td></td><td></td><td></td></tr>
<tr><td>Safety complaint</td><td>0.042*** (13.33)</td><td></td><td></td><td></td><td></td><td></td><td></td></tr>
<tr><td>Safety follow-up</td><td>-0.004 (-0.072)</td><td></td><td></td><td></td><td></td><td></td><td></td></tr>
</table></td><td>Funding: National Institute of Occupational Safety and Health

Conflict of Interest stated: no

Other comments: -</td></tr>
</table>

General	Study	Population	Exposure	Outcome	Results	Comments
		**Loss-to-follow-up:** NA<br><br>**Work characteristics:** manufacturing plants			(see table below)	

Safety programmed	0.07*** (20.83)						
Establishment size (base=50_99): number of employees							
1-10	0.025*** (4.89)	-0.016 (-0.87)	0.004 (0.33)	0.036*** (3.653)	0.015** (2.23)	0.02** (2.32)	-0.009 (-0.85)
11-19	0.009** (2.09)	-0.01 (-1.03)	-0.01 (-1.06)	0.033*** (3.03)	0.009 (1.39)	0.013 (1.60)	-0.002 (-0.18)
20-49	0.001 (0.31)	-0.006 (-1.06)	-0.006 (-0.93)	0.008 (1.23)	0.009** (1.98)	-0.002 (-31)	-0.004 (-0.51)
100-249	-0.002 (-0.86)	0.006 (1.08)	-0.004 (-0.74)	-0.001 (-0.21)	-0.014*** (-3.31)	-0.004 (-0.46)	-0.018** (2.00)
≥250	-0.014*** (-4.82)	0.017** (2.31)	0.005 (0,73)	-0.022*** (-3.92)	-0.034** (-6.77)	-0.001 (-0.10)	-0.022** (-2.06)
Union	-0.004* (-1.96)	0.016*** (3.16)	0.007* (1.65)	-0.018*** (-4.31)	-0.002 (-0.74)	-0.011* (-1.77)	-0.01 (-1.43)
Log (initial abatement)	0.006*** (8.65)	0.007*** (3.75)	0.005*** (3.03)	0.005*** (4.26)	0.004*** (3.59)	0.005*** (5.05)	0.004** (2.14)
Standard hazardousness	0.009*** (20.62)	0.017*** (16.36)	0.004*** (3.80)	0.011*** (12.05)	0.004*** (4.39)	0.006*** (3.71)	0.0001 (-0.04)
Standard category (base=training)							
Design	0.033*** (8.77)	0.095*** (10.15)	0.018** (2.04)	0.011 (1.38)	0.002 (0.23)	0.009 (1.09)	0.007 (0.58)
Engineering controls	0.038*** (6.87)	-0.252*** (-4.39)	0.107*** (11.30)	-0.273 (-4.26)	0.049*** (7.75)	~	0.052*** (4.92)
Guarding	0.042*** (14.09)	0.101*** (13.01)	0.009 (1.22)	0.039*** (7.18)	0.001 (0.21)	0.007 (1.03)	-0.006 (-0.60)
Hazard analysis	-0.003 (-0.48)	0.003 (0.25)	0.015 (1.41)	-0.023*	0.011 (1.35)	-0.015 (-1.15)	-0.022 (1.32)

General	Study	Population	Exposure	Outcome	Results	Comments

				* (-2.23)			
Housekeeping/ maintenance	0.043*** (9.62)	0.023 (1.54)	0.039*** (3.75)	0.051*** (6.64)	0.038*** (6.09)	0.023** (2.50)	0.001 (0.11)
Monitoring/ medical exams	0.036*** (10.13)	0.09*** (10.25)	0.029*** (3.33)	0.019*** (2.76)	0.015** (2.50)	0.011 (1.31)	0.003 (0.20)
Provide equipment	0.028*** (8.97)	0.045*** (5.48)	0.047*** (6.70)	0.011* (1.96)	0.021*** (4.14)	0.006 (0.88)	0.011 (1.13)
Provide information	0.046*** (12.50)	-0.009 (-0.75)	0.089*** (11.42)	-0.012 (-1.37)	0.055*** (10.46)	0.017** (2.09)	0.027*** (2.99)
Safe storage	0.011 (1.49)	0.023 (1.28)	-0.004 (-0.28)	0.019 (1.44)	-0.03* (1.83)	0.003 (0.24)	0.024 (1.52)
Written plan	-0.014*** (-3.53)	-0.026** (-2.40)	0.013 (1.46)	-0.048**** (-5.60)	0.005 (0.77)	-0.018** (-1.98)	0.007 (0.75)
Work practices	0.012*** (3.86)	0.029*** (3.65)	0.004 (0.45)	0.01* (1.95)	0.004 (0.70)	0.008 (1.34)	-0.015 (-1.31)
Own penalty quintiles (base=lowest quintile)							
Own penalty Q2	0.012*** (5.38)	0.032*** (5.77)	0.003 (0.64)	0.014*** (2.97)	0.001 (0.19)	0.005 (0.84)	0.012** (2.03)
Own penalty Q3	0.01*** (5.10)	0.029*** (5.96)	0.005 (1.19)	0.007 (1.62)	-0.001 (-0.18)	0.003 (0.58)	0.015** (2.38)
Own penalty Q4	0.013*** (6.47)	0.034*** (6.54)	0.001 (0.29)	0.013*** (3.38)	0.003 (0.87)	0.008 (1.51)	0.011* (1.67)
Own penalty Q5 (highest)	0.016*** (7.19)	0.036*** (6.08)	0.004 (0.73)	0.02*** (4.70)	0.002 (0.55)	0.015* (2.35)	0.014** (1.99)
Other penalty quintiles (base=lowest quintile)							
Other penalty Q2	-0.001 (-0.35)	0.004 (0.75)	-0.006 (-1.21)	0.003 (0.63)	-0.009** (-2.46)	-0.008 (-1.29)	0.006 (0.81)
Other penalty Q3	0.0004 (0.16)	0.001 (0.25)	-0.015*** (-3.01)	0.006 (1.47)	-0.001 (-0.25)	-0.006 (-1.04)	0.009 (1.19)

General	Study	Population	Exposure	Outcome	Results	Comments
					(continued below)	

Other penalty Q4	-0.001 (-0.23)	0.004 (0.66)	-0.012** (-2.37)	-0.001 (-0.20)	-0.002 (-0.48)	0.004 (0.62)	-0.006 (-0.78)
Other penalty Q5 (highest)	-0.012*** (-3.78)	0.005 (0.59)	-0.027*** (-3.09)	-0.017*** (-3.09)	-0.03*** (-5.78)	-0.003 (-0.37)	-0.008 (-0.97)
Number of observ.	181,040	43,974	27,593	47,796	32,123	18,196	11,110
Pseudo R^2	0.056	0.071	0.059	0.069	0.093	0.050	0.080
Log-likelihood	-43,033.6	-14,786.7	-5,830.9	11,417.3	-4,637.5	-2,840.2	-1,721.2

Inspec= inspections, progr=program, compl=complaint, follow=follow-up
t statistics in parentheses; *P < 0.10, **P < 0.05, ***P < 0.01.
~ = coefficient dropped - variable perfectly predicts the outcome. regression also includes dummies for two-digit SIC industries and year of following inspection

Short summary of findings:
- Health complaint inspections are the base group, and they have relatively low re-violation rates, similar to safety and health follow-up inspections. In contrast, both health and safety programmed inspections have significantly higher probabilities of re-violation.
- repeated violations low but higher re-violation for some standards -> standard characteristic influences re-violation -> but not always in same direction -> Standards whose violations rated as more hazardous or which received higher initial penalties tend to have more re-violations -> authors argue that this is because of inspector behaviour focussing more on these standards

company size: re-violation rates in safety programmed inspections do not vary greatly with size (but largest establishments highest rate, 1.7 percentage points above rate in base category) & smaller re-violation are for health complaint inspections for largest companies (reduction 3.4% points)
- unions: negative association between unions and re-violation rates (BUT: 15% higher re-violation rates for programmed safety inspections & 25% lower for complaint safety inspections than non-unionized)
- no strong pattern of industry
- longer abatement times have higher re-violation rates (doubling abatement period increase in re-violation rates of about 5–10%)

<table>
<tr><th>General</th><th>Study</th><th>Population</th><th>Exposure</th><th>Outcome</th><th>Results</th><th>Comments</th></tr>
<tr><td></td><td></td><td></td><td></td><td></td><td>- degree of hazard: benefits of compliance with standard increase with dangers resulting from violating it. BUT: increase of one unit in the hazard index (runs from 3 to 9) increases the re-violation rates (of 9 to 15%)
- categories of standard: Relative to Training standards (base category), other categories mostly higher re-violation rates; except for having a written plan: lower re-violation rates
- own penalty (= penalty for the violation considered): programmed and complaint safety inspections: higher penalties on target violation associated with re-violation rates 20 to 30 percent higher than the lowest penalty category; for health inspections: no relation
- Other penalties (= penalties for other violations): higher penalties linked to lower re-violations rates (25 to 80% lower) especially for the highest penalty quintile (roughly equivalent to total penalties exceeding $20,000)</td><td></td></tr>
<tr><td>First Author, Year
Fan, 2022
#2515

Extracted by: MS

Checked by:</td><td>Study name:-

Country: USA

Study design: quasi-experimental

Time of Study: 2000-2014 (since 2015: firms required to report work-related</td><td>Recruitment method/ Data source:
OSHA safety violation data; Name list of manufacturing firms from COMPUSTAT database

Matching criteria: NA

invited companies: NA

companies at baseline: n=25,273 observations from 2965 firms; average number of observations per firm: 8.52 (years) -> dataset 694 firms with at least 1 violation. N=4475 violations

Response: NA

companies at follow –up: NA</td><td>Description of intervention: Annual number of OSHA inspections

Study groups: NA

Time of measurement: 2000-2014</td><td>Outcome name: safety violation

Outcome definition and assessment: Annual number of OSHA violation, scaled by its 10,000 number of employees (‘000) in year t
-overall violation number
-Repeated is issued within 3 years of final order date of previous citation
-violation experience: total number of violations committed by a</td><td>Descriptive Statistics:
Tab.3a: Summary descriptive
<table>
<tr><th>Variable</th><th>Mean (SD)</th><th>Measure</th></tr>
<tr><td>Repeat violation rate (per 10000 employees)</td><td>0.0340 (1.0508)</td><td>Annual number of repeat violations per 10 thousand employees</td></tr>
<tr><td>Non-repeat violation rate (per 10000 employees)</td><td>1.0992 (14.2866)</td><td>Annual number of non-repeat violations per 10 thousand employee</td></tr>
<tr><td>Total violation rate (per 10000 employees)</td><td>1.1332 (14.2574)</td><td>Annual number of overall violations per 10 thousand employees</td></tr>
<tr><td>Inspection (times)</td><td>0.3178 (1.3977)</td><td>Annual number of OSHA inspections</td></tr>
<tr><td>Violation types in past experiences</td><td>0.0204 (0.1744)</td><td>Percentage of past willful and repeat violations in the firm’s total violation experiences</td></tr>
<tr><td>Past penalty (million USD)</td><td>0.0017 (0.0055)</td><td>Average penalty for a firm’s past violations</td></tr>
<tr><td>Violation experiences (times)</td><td>3.2898 (12.3233)</td><td>Total number of violations a firm committed in all years before the foc year</td></tr>
</table>

Statistical methods used: binomial regression; 1-year-lag between independent and dependent variable

Unadjusted/ adjusted estimates with precision (e.g. 95% confidence interval) for each outcome:
Violation types in past experience= Percentage of past willful and repeat violations in the firm’s total violation experiences;</td><td>Funding: Start-up Fund for New Recruits from The Hong Kong Polytechnic University (no. BE41), The Hong Kong Polytechnic University under research grant no. 99QP

Conflict of Interest stated: no

Other comments: -</td></tr>
</table>

General	Study	Population	Exposure	Outcome	Results	Comments
	hospitalizations within 24h; before 2015: report only when 3 or more workers hospitalized because of same incident)	**Loss-to-follow-up:** NA<br><br>**Work characteristics:** manufacturing firms (Standard Industry Classification code (SIC) 2000-3999)		firm in all years before year *t*<br><br>Control group: firms with no violation<br><br>**Time of measurement:** 2000-2014	Past penalty (million USD) = Average penalty for a firm's past violations;<br>Violation experiences (times) = Total number of violations a firm committed in all years before the focal year.	

Tab.4/ Tab. A appendix: Regression analysis for safety violations

	Model 1 (N=8294), full samples Repeat violation rate (of same clauses) Coef (SE)	Model 2 (N=25273), only firms with violations Non-repeat violation rate (of different clauses) Coef (SE)	Model 3 (N=25273) Total violation rate Coef (SE)
Inspection	0.0497** (0.011)	0.0063 (0.0470)	0.0566 (0.047)
Violation types in past experience	0.0958 (0.065)	0.839 (0.4949)	0.9421 (0.492)
Past penalty	5.1549 (3.953)	28.0712** (10.3850)	33.5683** (11.113)
Violation experience	-0.0024** (0.001)	0.0293** (0.0060)	0.0265** (0.006)
F	1.26**	7.45**	8.27**

two-tailed tests with dummy variables of year, industry, and state included; **, and * denote statistical significance at 0.01, and 0.05 levels

Tab.5: Analysis of reducing safety violations.

N=25273	Model 1 Non-repeat violation rate Moderating effect of penalty Coef. (SE)	Model 2 Non-repeat violation rate Moderating effect of OHSAS Coef. (SE)	Model 3 Total violation rate Moderating effect of penalty Coef. (SE)	Model 4 Total violatio[illegible] rate Moderating effect of OHSAS Coef. (SE)
Inspection	0.0068 (0.048)	-0.0201 (0.048)	0.0571 (0.048)	0.0297 (0.047
Violation types in past experiences	0.9814* (0.501)	0.9037 (0.495)	1.0935* (0.499)	1.0075* (0.493)
Past penalty	55.2416** (12.363)	28.1153** (10.375)	62.5822** (13.305)	33.6132** (11.105)
Violation experiences	0.0564** (0.008)	0.0383** (0.007)	0.0554** (0.008)	0.0356** (0.007)
Violation experiences*Past penalty	-4.7954** (0.668)		-5.1207** (0.689)	
F	7.56**	7.58**	8.37**	8.42**

two-tailed tests with dummy variables of year, industry, and state included; **, and * denote statistical significance at 0.01, and 0.05 levels

<table>
<tr><th>General</th><th>Study</th><th>Population</th><th>Exposure</th><th>Outcome</th><th>Results</th><th>Comments</th></tr>
<tr><td></td><td></td><td></td><td></td><td></td><td>(Appendix: more tests on the effect of violation experience on repeated violations)

Short summary of findings:
- coefficient indicates that with one more past violation experience, a firm’s subsequent repeat violation rate would be reduced by 0.0024 per 10,000 employees. Given an average repeat violation rate of 0.0340, there is a decrease of 7% (0.0024/0.034)
- inspections are positively correlated with repeat violations (of the same clause but not with non-repeated and total violations)
- no significant effect of inspections on reducing safety interventions
- With one more past violation experience, a firm’s subsequent non-repeat violation would increase to 0.0293 per 10,000 employees. Given an average non-repeat violation rate of 1.0992, there is an increase of 3%.
- Given an average total violation rate of 1.1332, one more past violation experience increases the total violation rate by 2%.
- past violation experiences are negatively correlated with subsequent repeat violations (violations of the same clause), but are positively correlated with non-repeat violations (violations of a different clause) and the number of overall violations (the sum of both)
- effectiveness of safety regulation enforcement is significantly enhanced when reinforced by stringent penalties</td><td></td></tr>
<tr><td>First Author, Year
Gray, 2023
#3726

Extracted by: MS

Checked by:</td><td>Study name:-

Country: USA

Study design: ecological study</td><td>Recruitment method/ Data source:
Fatality rates: Census of fatal occupational Injuries (CFOI) microdata;
Exclusion: self-employed workers; homicides & most highway fatalities, deaths on airplanes, trains, ships

Matching criteria: NA

invited companies: NA</td><td>Description of intervention:
Inspection rate

(=number of inspections divided by the number of construction establishmen</td><td>Outcome name: fatality rate

Outcome definition and assessment:
Fatality rate=fatalities per 100,000 workers
Confidential CFOI microdata;
N=1250 observations</td><td>Descriptive Statistics:
Tab.1: Summary statistics (1250 state-year observations; 50 states, 1992-2016)
<table>
<tr><th>Variable</th><th>Full sample Mean (Std. Dev.)</th><th>General construction Mean</th><th>Heavy construction Mean</th><th>Specialty trades Mean</th><th>Roofers</th></tr>
<tr><td>Fatality rate</td><td>11.049 (6.502)</td><td>8.76</td><td>17.24</td><td>10.33</td><td>32.51</td></tr>
<tr><td>Inspection rate</td><td>0.062 (0.045)</td><td>0.062</td><td>0.088</td><td>0.064</td><td>0.299</td></tr>
<tr><td>Consultation rate (Visits/ establishments)</td><td>0.012 (0.015)</td><td>0.012</td><td>0.012</td><td>0.012</td><td>0.012</td></tr>
</table></td><td>Funding: National Institute of Occupational Safety and Health

Conflict of Interest stated: no

Other comments: -</td></tr>
</table>

<table>
<tr><th>Genera l</th><th>Study</th><th>Population</th><th>Exposure</th><th>Outcome</th><th>Results</th><th>Comments</th></tr>
<tr>
<td></td>
<td>Time of Study: 1992-2016</td>
<td># companies at baseline: N=1250 state-year observations; 50 states

Response: NA

companies at follow –up: NA

Loss-to-follow-up: NA

Work characteristics: construction: General construction, Heavy construction, Specialty trades, Roofing</td>
<td>ts operating in that state)

state workers' compensation (WC) programs

Study groups: NA

Time of measurement: 1992-2016</td>
<td>Dependent variable = log(fatality rate + 1)

Time of measurement: 1992-2016</td>
<td>
<table>
<tr><td>Total visit rate (inspection + consultation)</td><td>0.074 (0.052)</td><td>0.066</td><td>0.099</td><td>0.076</td><td>0.310</td></tr>
<tr><td>Log(penalty)</td><td>7.16</td><td>7.21</td><td>7.09</td><td>7.19</td><td>7.22</td></tr>
<tr><td>WC waiting period (days)</td><td>5.005 (1.879)</td><td>-</td><td>-</td><td>-</td><td>-</td></tr>
<tr><td>Any WC firm size exemption (dummy)</td><td>0.166 (0.372)</td><td>-</td><td>-</td><td>-</td><td>-</td></tr>
</table>
WC= state workers' compensation programs

Statistical methods used: Multiple regression

Unadjusted/ adjusted estimates with precision (e.g. 95% confidence interval) for each outcome:

Tab.3: Determinants of workplace fatalities in construction
<table>
<tr><th></th><th>M5
Coeff (SE)</th><th>M6
Coeff (SE)</th><th>M7
Coeff (SE)</th></tr>
<tr><td>Inspection rate</td><td>-1.206** (0.560)</td><td>-0.559 (0.621)</td><td></td></tr>
<tr><td>Consultation rate</td><td></td><td>-5.224*** (1.802)</td><td></td></tr>
<tr><td>Total visit rate</td><td></td><td></td><td>-1.614*** (0.415)</td></tr>
<tr><td>Log(penalty)</td><td>-0.00713 (0.0485)</td><td>-0.0258 (0.0479)</td><td>-0.0305 (0.0501)</td></tr>
<tr><td>WC waiting period</td><td>0.0538*** (0.200)</td><td>0.0453** (0.0193)</td><td>0.0509** (0.0197)</td></tr>
<tr><td>WC size exemption</td><td>0.433*** (0.104)</td><td>0.420*** (0.100)</td><td>0.430*** (0.103)</td></tr>
</table>
Other variables: year dummies, Employment change, Unionization, log(wage), General construction, Specialty trades, New workers, Older workers, Total Hispanic, Foreign Hispanic, HS grads, Non-production workers, Seatbelt use, Smoking; *p < 0.10; **p < 0.05; ***p < 0.01

Tab.4: Determinants of workplace facilities in construction sub-sectors
<table>
<tr><th></th><th>General construction</th><th>Heavy construction</th><th>Specialty trades</th><th>Roofing</th></tr>
<tr><td colspan="5">Panel A</td></tr>
<tr><td>Inspection rate</td><td>-3.581** (1.352)</td><td>0.196 (0.728)</td><td>-1.589*** (0.530)</td><td>-0.479 (0.432)</td></tr>
<tr><td>log(penalty)</td><td>0.0384 (0.0596)</td><td>-0.0724 (0.0889)</td><td>0.0682* (0.0357)</td><td>0.151 (0.117)</td></tr>
<tr><td>WC waiting period</td><td>0.0803* (0.0401)</td><td>0.0551 (0.0442)</td><td>0.0844*** (0.0251)</td><td>0.247*** (0.0654)</td></tr>
<tr><td>WC size exemption</td><td>0.299 (0.193)</td><td>0.487** (0.200)</td><td>0.484*** (0.128)</td><td>0.600** (0.263)</td></tr>
</table>
</td>
<td></td>
</tr>
</table>

General	Study	Population	Exposure	Outcome	Results	Comments

R-squared	0.077	0.174	0.205	0.185
Panel B				
Inspection rate	-0.955 (1.568)	0.242 (0.730)	-0.974 (0.641)	-0.287 (0.401)
Consultation rate	-14.93*** (3.481)	-4.419 (4.463)	-5.769* (2.937)	-20.97*** (7.083)
log(penalty)	0.00616 (0.0535)	-0.099 (0.0890)	0.0553 (0.0394)	0.0547 (0.113)
WC waiting period	0.0586 (0.0372)	0.0471 (0.0452)	0.0743*** (0.0245)	0.212*** (0.0557)
WC size exemption	0.257 (0.181)	0.484** (0.196)	0.475*** (0.125)	0.581** (0.247)
R-squared	0.098	0.176	0.211	0.203
Panel C				
Total visit rate	-4.359*** (1.013)	-0.0691 (0.777)	-1.912*** (0.461)	-0.539 (0.425)
log(penalty)	0.00642 (0.0562)	-0.079 (0.0911)	0.0548 (0.0386)	0.146 (0.117)
WC waiting period	0.0714* (0.0383)	0.0568 (0.0440)	0.0809*** (0.0247)	0.245*** (0.0651)
WC size exemption	0.292 (0.187)	0.491** (0.200)	0.485*** (0.126)	0.598** (0.262)
R-squared	0.089	0.174	0.209	0.185

Other variables: year dummies, Employment change, Unionization, log(wage), New workers, Older workers, Total Hispanic, Foreign Hispanic, HS grads, Non-production workers, Seatbelt use, Smoking; *p < 0.10; **p < 0.05; ***p < 0.01

Tab. 6: greater significance for OSHA variables in state plan states and for WC variables in the federal OSHA states

	Federal OSHA states			State-plan OSHA states		
	1	2	3	4	5	6
Inspection rate	1.714 (2.591)	1.745 (2.474)		-1.320* (0,725)	-0.695 (0.660)	
Consultation rate		-3.674 (3.543)			-5.550*** (1.748)	
Total visit rate			-0.432 (1.452)			-1.703** (0.678)

General	Study	Population	Exposure	Outcome	Results	Comments
					(continued below)	

Log(penalty)	0.092 (0.0812)	0.0943 (0.0845)	0.0731 (0.0759)	-0.105 (0.0662)	-0.108* (0.0593)	-0.119* (0.0655)
WC waiting period	0.0566** (0.0226)	0.0531** (0.0219)	0.0560** (0.0235)	0.0738 (0.0510)	0.0474 (0.0505)	0.067 (0.0482)
WC size exemption	0.542*** (0.143)	0.519*** (0.139)	0.542*** (0.148)	0.193 (0.195)	0.248 (0.195)	0.213 (0.192)

Multiple regression model with year dummies included but not reported. Dependent variable = log(fatality rate + 1), based on microdata from Census of Fatal Occupational Injuries. Standard errors in parentheses, clustered by state.*p < 0.10; **p < 0.05; ***p < 0.01

Tab.7: firm size (small (1–10 employees) versus big firms (100+ employees):

	Small firms (1–10 employees)			Big firms (100+ employees)		
	1	2	3	4	5	6
Inspection rate	-0.348 (1.423)	0.147 (1.352)		-0.825 (1.240)	-0.605 (1.436)	
Consultation rate		-4.045 (4.933)			-1.783 (4.478)	
Total visit rate			-0.647 (1.213)			-0.907 (0.916)
Log(penalty)	-0.00236 (0.0951)	-0.017 (0.101)	-0.0165 (0.0969)	-0.0145 (0.112)	-0.0209 (0.109)	-0.0226 (0.106)
WC waiting period	0.131*** (0.0437)	0.125*** (0.0447)	0.130*** (0.0438)	0.0537 (0.0365)	0.0508 (0.0357)	0.0521 (0.0366)
WC size exemption	0.825*** (0.223)	0.816 (0.223)	0.824*** (0.223)	0.433** (0.195)	0.428** (0.193)	0.431* (0.194)

Multiple regression model with year dummies included but not reported. Dependent variable = log(fatality rate + 1), based on microdata from Census of Fatal Occupational Injuries. Standard errors in parentheses, clustered by state.*p < 0.10; **p < 0.05; ***p < 0.01

Short summary of findings:

- higher inspection rates associated with lower fatality rates (for general construction and specialty trades)

<table>
<tr><th>General</th><th>Study</th><th>Population</th><th>Exposure</th><th>Outcome</th><th>Results</th><th>Comments</th></tr>
<tr><td></td><td></td><td></td><td></td><td></td><td>One standard deviation increase in inspection rate (about a 4-percentage point increase over the mean of 6%) is associated with about a 5% reduction in fatality rate. For consultation rate and total visit rate, one standard deviation increases are associated with larger reductions in the fatality rate (9%–13%).
- longer WC waiting period and WC size exemption associated with higher fatality rates
- differences in federal OSHA states and state plan states</td><td></td></tr>
<tr><td>First Author, Year
Raghunandan, 2024
#3494

Extracted by: MS

Checked by:</td><td>Study name:-

Country: USA

Study design: Ecological study

Time of Study: 2002-2016</td><td>Recruitment method/ Data source:
OSHA inspection and violation data from U.S. Department of Labor’s Enforcement Data Web page: firms with no violation or inspection excluded

Matching criteria: NA

invited companies: NA

companies at baseline: Regression sample: N=430,015

Response: NA

companies at follow –up: NA

Loss-to-follow-up: NA

Work characteristics: no restriction</td><td>Description of intervention: Violation

ViolAny
Unit measurement: Firm-year
Definition: Indicator variable that equals 1 if firm I committed at least one OSHA violation in year t, in any state

Unit measurement: Firm-state-year
ViolInStateijt = indicator for whether firm i</td><td>Outcome name: Inspection (programmed, unprogrammed); (re-) violations

Outcome definition and assessment:
CleanOutOfStateijt = indicator for whether firm i was inspected and had no violation in any state other than j in year t.
AnyInspectionInStateijt = indicator for whether OSHA conducts an inspection of firm i in state j in year t.
ReactiveInStateijt, an indicator for whether OSHA conducts a reactive inspection (i.e., in response to a trigger event) but no centrally</td><td>Descriptive Statistics:
Tab.2: Descriptive statistics
<u>OSHA firm-state-year violation rates by year</u>
OSHA violations: 0.039
OSHA inspections: 0.063
Discretionary OSHA inspections: 0.006

Tab.2: Descriptive statistics
<table><tr><th>Variable</th><th>Mean</th><th>Median</th><th>Std. Dev.</th></tr><tr><td>ViolAnyt</td><td>0.472</td><td>0.000</td><td>0.499</td></tr><tr><td>ViolInStatet</td><td>0.039</td><td>0.000</td><td>0.194</td></tr><tr><td>ViolOutOfStatet</td><td>0.461</td><td>0.000</td><td>0.499</td></tr><tr><td>RWInState</td><td>0.002</td><td>0.000</td><td>0.043</td></tr><tr><td>AnyInspectionInState</td><td>0.063</td><td>0.000</td><td>0.243</td></tr><tr><td>ReactiveInState</td><td>0.033</td><td>0.000</td><td>0.178</td></tr><tr><td>PlannedInState</td><td>0.024</td><td>0.000</td><td>0.152</td></tr><tr><td>DiscInState</td><td>0.006</td><td>0.000</td><td>0.079</td></tr><tr><td>CleanInState</td><td>0.024</td><td>0.000</td><td>0.153</td></tr><tr><td>CleanOutOfState</td><td>0.123</td><td>0.000</td><td>0.328</td></tr></table>
Statistical methods used: logistic regression

Unadjusted/ adjusted estimates with precision (e.g. 95% confidence interval) for each outcome:

Tab.3: Response to past violations
<table><tr><th></th><th colspan="2">Response to past violations</th></tr><tr><td></td><td>linear</td><td>linear</td></tr><tr><td>ViolAnyt</td><td>-0.0579*** (-7.44)</td><td>-0.0708*** (-9.43)</td></tr></table></td><td>Funding: NR

Conflict of Interest stated: no

Other comments:
- dependent variables: Inspection Exposure: Violation</td></tr>
</table>

<table>
<tr><th>General</th><th>Study</th><th>Population</th><th>Exposure</th><th>Outcome</th><th>Results</th><th>Comments</th></tr>
<tr>
<td></td>
<td></td>
<td></td>
<td>violates an OSHA rule in state j in year t.
ViolOutOfStateijt = indicator for whether firm i violates an OSHA rule in any state other than j in year t.
RWInState = indicator for whether OSHA assesses a Repeat or Willful violation for the firm in a given state in a given year.

Study groups: NA

Time of measurement: 2002-2016</td>
<td>planned or discretionary inspections of firm i in state j in year t.
DiscInStateijt = indicator for whether OSHA conducts a discretionary inspection of firm i (i.e., an inspection undertaken at the discretion of a state-level OSHA field office) in state j in year t.
PlannedInStateijt = indicator for whether there was at least one “planned” inspection (i.e., proactively requested by OSHA headquarters) of firm i in state j in year t but no inspections undertaken at the discretion of the state office
CleanInStateijt = indicator variable for whether firm i was inspected and had no violation in state j in year t.</td>
<td>
<table>
<tr><td>lnEstabst</td><td></td><td>0.0513*** (8.81)</td></tr>
<tr><td>lnAssetst</td><td></td><td>0.0568*** (7.86)</td></tr>
<tr><td>ROA</td><td></td><td>0.0426 (1.30)</td></tr>
<tr><td>Leverage</td><td></td><td>-0.0061 (-0.21)</td></tr>
<tr><td>MB</td><td></td><td>-0.0008 (-1.15)</td></tr>
<tr><td>Controls</td><td></td><td></td></tr>
<tr><td>Controls</td><td>No</td><td>Yes</td></tr>
<tr><td>Year FE</td><td>Yes</td><td>Yes</td></tr>
<tr><td>Firm by State FE</td><td>Yes</td><td>Yes</td></tr>
<tr><td>Adjusted/ Pseudo R^2</td><td>0.3775</td><td>0.3841</td></tr>
<tr><td>Obs.</td><td>25,594</td><td>25,594</td></tr>
</table>
LogAssetsit = Natural logarithm of firm-year total assets, lnEstabst = Natural logarithm of the number of distinct establishments firm i operates in state j in year t, ROA= Return on assets, measured as ratio of net income to lagged assets, leverage = ratio of total short- and long-term debt to assets, MB= Market to book ratio. Robust standard errors are clustered by firm and corresponding t-statistics are presented in brackets. The markings ***, **, and * denote results significant at the 1%, 5%, and 10% levels.

- committing an OSHA violation in any location in one year is negatively associated with committing an OSHA violation in any location in the next year
- estimate in column 2 suggests a 7.1 percentage point lower violation rate, or a 22.5% reduction in the likelihood of a violation relative to the percentage of firm-years with a violation in the sample (31.5%, untabulated)

Tab.4: Geographic Shifts in Violating Behavior
<table>
<tr><td></td><td colspan="3">ViolInStatet+1</td></tr>
<tr><td></td><td>Linear
OLS estimate</td><td>Linear
OLS estimate</td><td>Linear
OLS estimate</td></tr>
<tr><td>ViolInState</td><td>-0.00536*** (-12.00)</td><td>-0.0558*** (-12.58)</td><td>-0.0460*** (-9.11)</td></tr>
<tr><td>ViolOutOfStatet</td><td>0.0045*** (5.96)</td><td>0.0027*** (3.63)</td><td>0.0511*** (7.08)</td></tr>
<tr><td>Controls:</td><td></td><td></td><td></td></tr>
<tr><td>Controls</td><td>No</td><td>Yes</td><td>Yes</td></tr>
<tr><td>Year FE</td><td>Yes</td><td>Yes</td><td>No</td></tr>
<tr><td>Firm by State FE</td><td>Yes</td><td>Yes</td><td>Yes</td></tr>
<tr><td>Firm by Year FE</td><td>No</td><td>No</td><td>Yes</td></tr>
<tr><td>State by Year FE</td><td>No</td><td>No</td><td>Yes</td></tr>
<tr><td>Adjusted/Pseudo R2</td><td>0.1709</td><td>0.1723</td><td>0.1761</td></tr>
<tr><td>Obs.</td><td>432,307</td><td>430,015</td><td>427,819</td></tr>
</table>
Column 1 includes only year and firm-by-state fixed effects, columns 2, and 3 include additional controls. Finally, column 3 includes both firm-by-year and stateby-year fixed effects. Coefficients are omitted for brevity, but control variables include the natural log of
</td>
<td></td>
</tr>
</table>

General	Study	Population	Exposure	Outcome	Results	Comments
				**Time of measurement:** 2002-2016	the number of establishments the firm has in the state, the natural log of assets of the firm, return on assets, leverage, and market to book. Robust standard errors are clustered by firm and corresponding *t*-statistics are presented in brackets. The markings ***, **, and * denote results significant at the 1%, 5%, and 10% levels.<br><br>-violations in a given state are associated with fewer violations in the same state in the next year; but violations in out-of-state facilities are associated with more in-state violations the next year<br>- average firm-state-year having an in-state violation rate of 3.9%, but an out-of-state violation rate of 47.2%<br><br>Tab. 5: Within-State Compliance Effort Shifting	

	ViolOutOfStatei,j,t+1 OSL estimate	ViolSameStatehij,t+1 OSL estimate
$ViolInState_t$	0.112*** (2.99)	
ViolEstabhijt		-0.0672*** (-14.55)
$ViolOutOfState_t$	-0.0781*** (-7.96)	0.0078* (1.87)
ViolInStateijt × SameStateEstabPcti,j,t+1	0.0091 (0.46)	
SameStateEstabPcti,j,t+1	-0.2052*** (-7.75)	
Controls		
Controls	Yes	Yes
Year FE	Yes	Yes
Firm by State FE	Yes	Yes
Adjusted/ Pseudo R^2	0.4386	0.3213
Obs.	430,015	6,315,010

Column 1 sample is a firm-state-year panel from 2002 to 2016, column 2 uses an establishment-level panel. Coefficients are omitted for brevity, but control variables include the natural log of the number of establishments the firm has in the state, the natural log of assets of the firm, return on assets, leverage, and market to book. Robust standard errors are clustered by firm and corresponding *t*-statistics are presented in brackets. The markings ***, **, and * denote results significant at the 1%, 5%, and 10% levels.

- firms do not shift violating behaviour within states

Tab.6: OSHA inspections: AnyInspectionInState

	$AnyInspectionInState_{t+1}$	
	linear	linear
$ViolInState_t$	-0.0249*** (-5.19)	-0-0284*** (-6.07)
$ViolOutOfState_t$	0.0072*** (6.96)	0.0040*** (3.98)
$CleanInState_t$	-0.0563*** (-12.30)	-0.0591*** (-13.13)
$CleanOutOfState_t$	-0.005 (-0.41)	-0.0020* (-1.88)
Controls		
Controls	No	Yes

General	Study	Population	Exposure	Outcome	Results	Comments
					(see below)	

Year FE	Yes	Yes
Firm by State FE	Yes	Yes
Adjusted/ Pseudo R^2	0.2281	0.2301
Obs.	432,307	430,015

Columns 1 includes year fixed effects and firm-by-state fixed effects, whereas columns 2 includes additional controls. Coefficients are omitted for brevity, but control variables include the natural log of the number of establishments the firm has in the state, the natural log of the firm's assets, return on assets, leverage, and market to book. Columns use a linear probability model. Robust standard errors are clustered by firm and corresponding *t*-statistics are presented in brackets. The markings ***, **, and * denote results significant at the 1%, 5%, and 10% levels.

Tab.6: OSHA inspections: ReactiveInState

	$ReactiveInState_{t+1}$	
	linear	linear
$ViolInState_t$	-0.0248*** (-7.39)	-0.0267*** (-8.04)
$ViolOutOfState_t$	0.0048*** (6.36)	0.0032*** (4.47)
$CleanInState_t$	-0.0350*** (-10.07)	-0.0363***(-10.50)
$CleanOutOfState_t$	0.0001 (0.07)	-0.0008 (-0.97)
Controls		
Controls	No	Yes
Year FE	Yes	Yes
Firm by State FE	Yes	Yes
Adjusted/ Pseudo R^2	0.1369	0.1375
Obs.	432,307	430,307

Columns 1 includes year fixed effects and firm-by-state fixed effects, whereas columns 2 includes additional controls. Coefficients are omitted for brevity, but control variables include the natural log of the number of establishments the firm has in the state, the natural log of the firm's assets, return on assets, leverage, and market to book. Columns use a linear probability model. Robust standard errors are clustered by firm and corresponding *t*-statistics are presented in brackets. The markings ***, **, and * denote results significant at the 1%, 5%, and 10% levels.

Tab.6: OSHA inspections: *PlannedInState*

	$PlannedInState_{t+1}$	
	linear	linear
$ViolInState_t$	-0.0176*** (-5.02)	-0.0188*** (-5.41)
$ViolOutOfState_t$	0.0021*** (3.71)	0.0010* (1.66)
$CleanInState_t$	-0.0173*** (-6.05)	-0.0183*** (-6.44)
$CleanOutOfState_t$	0.0003 (0.44)	-0.0002 (-0.27)
Controls		
Controls	No	Yes
Year FE	Yes	Yes
Firm by State FE	Yes	Yes
Adjusted/ Pseudo R^2	0.1317	0.1324
Obs.	432,307	430,015

General	Study	Population	Exposure	Outcome	Results	Comments
					(see below)	

Columns 1 includes year fixed effects and firm-by-state fixed effects, whereas columns 2 includes additional controls. Coefficients are omitted for brevity, but control variables include the natural log of the number of establishments the firm has in the state, the natural log of the firm's assets, return on assets, leverage, and market to book. Columns use a linear probability model. Robust standard errors are clustered by firm and corresponding *t*-statistics are presented in brackets. The markings ***, **, and * denote results significant at the 1%, 5%, and 10% levels.

Tab.6: OSHA inspections: *DiscInState*

	$DiscInState_{t+1}$	
	linear	linear
$ViolInState_t$	0.0175*** (9.80)	0.0171*** (9.69)
$ViolOutOfState_t$	0.0002 (0.69)	-0.0003 (-0.74)
$CleanInState_t$	-0.0040** (-2.14)	-0.0045** (-2.39)
$CleanOutOfState_t$	-0.0008** (-2.07)	-0.0010*** (-2.62)
Controls		
Controls	No	Yes
Year FE	Yes	Yes
Firm by State FE	Yes	Yes
Adjusted/ Pseudo R^2	0.0459	0.0459
Obs.	432,307	430,015

Columns 1 includes year fixed effects and firm-by-state fixed effects, whereas columns 2 includes additional controls. Coefficients are omitted for brevity, but control variables include the natural log of the number of establishments the firm has in the state, the natural log of the firm's assets, return on assets, leverage, and market to book. Columns use a linear probability model. Robust standard errors are clustered by firm and corresponding *t*-statistics are presented in brackets. The markings ***, **, and * denote results significant at the 1%, 5%, and 10% levels.

- inspections are lower (higher) in year *t*+1 subsequent to in-state (out-of-state) violations in year *t*
- negative and significant relation between in-state clean inspections in year *t* and the likelihood of inspection in *t*+1
- relation between out-of-state violations in year *t* and reactive inspections in year *t*+1
- weaker evidence between out-of-state violations in year *t* and planned inspections in year *t*+1, and no relation between out-of-state violations in year *t* and discretionary inspections in year *t*+1

Tab.7.: Repeat and willful violations (*RWInState)*

	$RWInState_{i,j,t+1}$	
	1<br>OLS estimate	2<br>OLS estimate
$ViolInState_t$	0.0035*** (3.52)	0.0034*** (3.39)

<table>
<tr><th>General</th><th>Study</th><th>Population</th><th>Exposure</th><th>Outcome</th><th>Results</th><th>Comments</th></tr>
<tr><td></td><td></td><td></td><td></td><td></td><td>
<table>
<tr><td>ViolOutOfState$_t$</td><td>0.0002 (1.29)</td><td>0.0001 (0.76)</td></tr>
<tr><td colspan="3">Controls</td></tr>
<tr><td>Controls</td><td>No</td><td>Yes</td></tr>
<tr><td>Year FE</td><td>Yes</td><td>Yes</td></tr>
<tr><td>Firm by State FE</td><td>Yes</td><td>Yes</td></tr>
<tr><td>Adjusted/ Pseudo R^2</td><td>0.0524</td><td>0.0521</td></tr>
<tr><td>Obs.</td><td>432,307</td><td>430,015</td></tr>
</table>
Additional interaction terms, using a firm-state-year panel from 2002 to 2016. Column 1 includes year fixed effects and firm-by-state fixed effects. Column 2 includes additional controls. Coefficients are omitted for brevity, but control variables include the natural log of the number of establishments the firm has in the state, the natural log of assets of the firm, return on assets, leveage, and market to book. Robust standard errors are clustered by firm and corresponding t-statistics are presented in brackets. The markings ***, **, and * denote results significant at the 1%, 5%, and 10% levels.

Short summary of findings:
- firms caught violating in one state subsequently violate less in that state but violate more in other states.
- violations in one state do not trigger proactive OSHA inspections in other states:out-of-state violations impact reactive investigations (those based on whistleblowers or injuries), but not centrally planned inspections or those taken at the discretion of local offices.
- firms face lower monetary penalties when subsequent violations occur across state lines
- firms are more likely to shift violating behavior into states with greater information frictions
- violations in one state lead to an increase in severe (repeat or willful) penalties in the same state, but the severe penalty rate decreases for firms previously violating in a different state
</td><td></td></tr>
<tr><td>First Author, Year
Johnson, 2023
#3041

Extracted by: MS

Checked by:</td>
<td>Study name:-

Country: USA

Study design: difference-in-difference (DID) (=</td>
<td>Recruitment method/ Data source:
OSHA's annual SST target lists (2011-2010); b) OSHA's annual ODI survey data on injuries and employment (1996-2011); OSHA inspection data from Integrated Management Information System (IMIS) (1990-2014), d) annual Dun and Bradstreet data from</td>
<td>Description of intervention:
Inspection (Site-Specific Targeting (SST) program)

Number of OSHA</td>
<td>Outcome name:
(serious) Injuries

Outcome definition and assessment:
-Serious injuries/100 FTE = Serious injuries are those that cause days away from work.</td>
<td>Descriptive Statistics:
Tab.1. Summary statistics for randomized sample (+/ - 4 years from directive year)
<table>
<tr><th></th><th>N</th><th>Mean</th><th>SD</th><th>Median</th></tr>
<tr><td>Number of serious injuries [ODI]</td><td>90,343</td><td>6.5</td><td>8.9</td><td>4.0</td></tr>
<tr><td>Serious injuries/100 FTE [ODI]</td><td>90,343</td><td>3.9</td><td>3.6</td><td>3.1</td></tr>
<tr><td>Injuries with days away, restricted or transfer/100 FTE (DART rate) [ODI]</td><td>90,343</td><td>7.6</td><td>5.0</td><td>6.7</td></tr>
<tr><td>Total hours worked, 000s [ODI]</td><td>90,346</td><td>284.8</td><td>331.2</td><td>183.0</td></tr>
</table></td>
<td>Funding: Laura and John Arnold Foundation, the Harvard Business School Division of Research, and the Department of Labor DOL Scholars Program

Conflict of Interest stated: yes</td></tr>
</table>

<table>
<tr><th>General</th><th>Study</th><th>Population</th><th>Exposure</th><th>Outcome</th><th>Results</th><th>Comments</th></tr>
<tr>
<td></td>
<td>controlled before-after study)

Time of Study: 1990-2014; Sampling period: 2001-2010</td>
<td>National Establishment Time Series (NETS) (1990-2013)
Sample construction: DUNS numbers linked to establishments on any of OSHA’s SST, and records in ODI and NETS; SST target list linked to IMIS records by fuzzy-matching names; Creation of random sample according to pre-defined criteria -> randomly assigned for an SST inspection (treatment group) or were eligible but not assigned (control group).

Matching criteria: NA

invited companies: NA

companies at baseline:
Sampling period: 2001-2010: SST target list ranged from 8,404 to 12,153 establishments (total: 101,463)
N=101,463 establishment-directive observations from 40,946 unique establishments

Response: NA

companies at follow –up: NA

Loss-to-follow-up: NA</td>
<td>inspections in calendar year = include those triggered by an incident (i.e., a serious accident, complaint, or referral) or pre-planned via one of OSHA’s programs (including SST)

Study groups: Treatment group: randomly assigned for an SST inspection; control group: were eligible but not assigned to inspection

Time of measurement:</td>
<td>-Injuries with days away, restricted or transfer/100 FTE (DART rate) = reported as number of injuries per 100 full-time employees (FTE). Calculated by dividing number of injuries in a calendar year by the number of FTE (calculated as total number of hours worked divided by 2,000), and multiplying this ratio by 100.

Time of measurement:</td>
<td>
<table>
<tr><td>Average number of employees [ODI]</td><td>90,346</td><td>149.5</td><td>176.8</td><td>96.0</td></tr>
<tr><td>Number of employees [NETS]</td><td>137,566</td><td>135.1</td><td>153.1</td><td>89.0</td></tr>
<tr><td>Number of OSHA inspections in calendar year[IMIS]</td><td>143,757</td><td>0.2</td><td>0.5</td><td>0.0</td></tr>
<tr><td>Number of SST inspections in calendar year [IMIS]</td><td>143,757</td><td>0.1</td><td>0.3</td><td>0.0</td></tr>
</table>

-baseline characteristics balanced between establishment assigned and not assigned to SST inspection

Statistical methods used: exclusion of large outliers of the number of serious injuries per year -> variable was top-coded at its ninety-ninth percentile (which is 54); regression models; Regressions include area-office–directive and τ-year (number of years since the directive year) as fixed effects, and mean of establishment’s dependent variable (or log(1 + dependent variable) in Poisson regressions) over the 4 years prior to directive year and number of years over which this baseline mean is calculated.
<u>historical injuries, predicted injuries, and estimated treatment effects:</u> In each scenario: same number of establishments each year as were assigned under the actual SST policy: 16,861 over the 10-year period.

Unadjusted/ adjusted estimates with precision (e.g. 95% confidence interval) for each outcome:

Tab.3: Regression-estimated effects of SST Inspection on serious injuries
<table>
<tr><td></td><td colspan="2"># of serious injuries</td><td>SST inspected</td><td># of serious injuries</td></tr>
<tr><td></td><td colspan="2">Intention-to-treat</td><td>(First stage)</td><td>Treatment-on treated</td></tr>
<tr><td></td><td>1</td><td>2</td><td>3</td><td>4</td></tr>
<tr><td>Assigned to SST inspection</td><td>−0.035 (0.017)</td><td>−0.037 (0.017)</td><td>0.458 (0.006)</td><td></td></tr>
<tr><td>SST-inspected</td><td></td><td></td><td></td><td>−0.091 (0.042)</td></tr>
<tr><td>log(hours) in t − 2</td><td></td><td>0.258 (0.019)</td><td></td><td>0.255 (0.019)</td></tr>
</table>
</td>
<td>Other comments: -</td>
</tr>
</table>

<table>
<tr><th>General</th><th>Study</th><th>Population</th><th>Exposure</th><th>Outcome</th><th>Results</th><th>Comments</th></tr>
<tr><td></td><td></td><td>Work characteristics: no restrictions</td><td></td><td></td><td>
<table>
<tr><td>Average marginal effect</td><td>−0.19</td><td>−0.20</td><td></td><td>−0.48</td></tr>
<tr><td>Specification</td><td>Poisson</td><td>Poisson</td><td>OLS</td><td>IV-Poisson</td></tr>
<tr><td># observations</td><td>40,993</td><td>40,993</td><td>40,993</td><td>40,993</td></tr>
<tr><td># establishment-directives</td><td>13,736</td><td>13,736</td><td>13,736</td><td>13,736</td></tr>
<tr><td># establishments</td><td>11,083</td><td>11,083</td><td>11,083</td><td>11,083</td></tr>
<tr><td># area-office–directives</td><td>383</td><td>383</td><td>383</td><td>383</td></tr>
<tr><td>Mean dep var, estabs not assigned</td><td>5.35</td><td>5.35</td><td>0.17</td><td>5.35</td></tr>
</table>
SEs, in parentheses, are clustered by establishment. Columns 1–2: Poisson regression estimates of effect of being assigned to SST inspection on an establishment’s annual number of serious injuries (intent- to-treat estimates). Column 3: OLS estimate of tincreased probability (in percentage points) that establishments assigned to SST inspection in directive year actually received an SST inspection. Column 4: IV-Poisson regression effect of receiving an SST inspection on an establishment’s annual number of serious injuries.

- establishments assigned to SST inspection experience 3.4 percent fewer injuries over the directive year and four following years (β = −0.035, SE = 0.017, p = 0.04) than do those not assigned.
- average marginal effects: being assigned to inspection leads to decline of 0.19 in annual number of serious injuries from the sample annual average of 5.35 (similar results when controlling for baseline log total working hours from two years before the directive year (year t − 2) (column 2)).
- OSL for estimating treatment-on-the-treated effect: Being assigned to inspection increases probability of actually being inspected over the directive year and four following years by 46 percentage points (p < 0.01) above the 17% inspection rate over this period among those not assigned in the directive year (column 3).
- Column 4: effect of an SST inspection on serious injuries: SST inspections lead to 8.7 percent fewer serious injuries per year (β = −0.091, SE = 0.042, p = 0.03). Average marginal effect indicates SST inspections lead to an average decline of 0.48 in annual number of serious injuries (-> average SST inspection averted (5 × 0.48 =) 2.4 serious injuries over the five-year period.)

Tab.5: Number of serious injuries OSHA averts under alternative targeting policies
</td><td></td></tr>
</table>

General	Study	Population	Exposure	Outcome	Results	Comments
					(see table below)	

Policy (1)	Targeting metric (2)	Average # annual serious averted per establishment, among those assigned (3)	Number of establishments assigned to inspection on the… high-priority list (4)	low-priority list (5)	Total number of serious injuries averted over 5 years (6)	Additional injuries averted compared to historical policy (7)
OSHA's policy[a]	DART rate, t − 2[b]	−0.168 (−0.193; −0.140)	12,458	4,403	14,163	
Inspect those with highest metric, preserving the historical total number of inspections	Historical injuries	−0.312 (−0.393; −0.222)	16,861	0	26,303	12,140
	Predicted injuries	−0.357 (−0.491; −0.227)	16,861	0	30,097	15,934
	Estimated treatment effect	−0.364 (−0.620; 0.072)	16,861	0	30,687	16,524
Inspect those with highest metric, preserving the historical total cost of inspections	Historical injuries	−0.332 (−0.414; −0.233)	15,126	0	25,109	10,945
	Predicted injuries	−0.419 (−0.567; −0.274)	14,944	0	31,308	17,144
	Estimated treatment effect	−0.393 (−0.681; 0.050)	15,223	0	29,914	15,751
Maintain size and Pr(inspection) of highand low-priority lists from	Historical injuries	−0.240 [−0.363; −0.118]	11,788	4,276	19,277	5,114
	Predicted injuries	−0.241 (−0.387; −0.083)	11,798	4,188	19,262	5,099
	Estimated treatment	−0.205 (−0.365; −0.046)	11,890	4,307	16,602	2,439

<table>
<tr><th>General</th><th>Study</th><th>Population</th><th>Exposure</th><th>Outcome</th><th>Results</th><th>Comments</th></tr>
<tr><td></td><td></td><td></td><td></td><td></td><td>
<table>
<tr><td>historical policy, preserving the historical total cost of inspections</td><td>effect</td><td></td><td></td><td></td><td></td><td></td></tr>
</table>
[a]=high-priority= OSHA's "primary list";low-priority = OSHA's secondary list; [b]=Injuries with days away, restricted or transferred/ 100 FTE, from two years prior to the directive year. Column 3: Each reported estimate= median of average of two coefficient across 250 random splits of sample into training and holdout samples: one coefficient where the machine learning models are trained on training sample and regression is estimated on the holdout sample, the other where roles of the training and holdout samples are reversed- 95% CI in brackets. Colum6=number of establishments assigned to inspection (the sum of columns 4 and 5), multiplied by (negative of) average treatment effect among assigned establishments (column 3), multiplied by 5 (the window of years over which effects of assignment to inspection are estimated)

- historical injuries: would have averted an average of 0.312 (90 percent CI of −0.393 to −0.222, Row 2) serious injuries per year among assigned establishments, only 0.168 averted under the historical SST policy (row 1).
- highest predicted injuries: would have averted an average of 0.357 (90 percent CI of−0.491 to −0.227, row 3) serious injuries per year, (averting 2.1 times as many as averted under the historical SST policy).
- highest estimated treatment effects: averted 0.364 (90 percent CI of −0.620 to 0.072, row 4) serious injuries per year, 2.2 times as many as averted under the historical SST policy
- total number of establishments assigned to inspection: total number of injuries that OSHA would avert under each policy: historical injuries 12,140, predicted injuries 15,934, and estimated treatment effects: 16,524
- OSHA could avert an average of 0.24 injuries per year per assigned establishment if this targeting policy were based on historical injuries (90 percent CI of −0.363 to −0.118; see Table 5 row 8), 0.241 injuries per year per assigned establishment if based on predicted injuries (90 percent CI of −0.387 to−0.083, row 9), or 0.205 injuries per year per assigned establishment if based on estimated treatment effects (90 percent CI of −0.365 to −0.046, row 10). These correspond to 136
</td><td></td></tr>
</table>

<table>
<tr><th>General</th><th>Study</th><th>Population</th><th>Exposure</th><th>Outcome</th><th>Results</th><th>Comments</th></tr>
<tr><td></td><td></td><td></td><td></td><td></td><td>percent, 136 percent, and 117 percent, respectively, of the number of injuries averted under the SST policy.

Short summary of findings:
- each inspection led to 2.4 (9 %) fewer serious injuries over the next 5 years
- OSHA could have averted as much as twice as many injuries by targeting inspections to workplaces with the highest expected averted injuries and nearly as many by targeting the highest expected level of injuries</td><td></td></tr>
<tr><td>First Author, Year
Payá castiblanque, 2020
#google scholar

Extracted by: MS

Checked by:</td><td>Study name:
ESENER-2-Spain

Country:
Spain

Study design:
Cross-sectional

Time of Study:
14 July - 20 October 2014</td><td>Recruitment method/ Data source:
microdata from the Second European Survey of Companies on New and Emerging Risks (ESENER-2-Spain) from by the National Institute of Safety and Hygiene in the Workplace (INSHT):
-interviews of “the person who knows most about occupational safety and health” in opinion of company manager

Matching criteria: NA

invited companies:
Establishments with 5 or more employees in NACE Rev.2 sections A-S: n=454.000*

# companies at baseline:
n=3.162 work centres in Spain with ≥5 workers</td><td>Description of intervention:
Inspections within last 3 years

“Has your workplace received any visits from the Labour Inspectorate in the last 3 years to check compliance with risk prevention regulations? work?”(Q165)

Study groups: NA

Time of measuremen</td><td>Outcome name:
absenteeism

Outcome definition and assessment:
“How would you describe the level of absenteeism at your workplace compared to other workplaces in the industry?” (Q450)
Response: very high, quite high, within the average, quite low or very low

Time of measurement: 14 July -20 October 2014</td><td>Descriptive Statistics:
Tab.A1: Descriptive analysis of multinomial logistic regression
<table>
<tr><th>Variable</th><th>Number (%)</th></tr>
<tr><td colspan="2">Level of absenteeism</td></tr>
<tr><td>Very low</td><td>1663 (54.0)</td></tr>
<tr><td>Quite low</td><td>650 (21.1)</td></tr>
<tr><td>Within half</td><td>614 (19.9)</td></tr>
<tr><td>Very high or quite high</td><td>151 (4.9)</td></tr>
<tr><td colspan="2">Inspections visits within last 3 years</td></tr>
<tr><td>No</td><td>1700 (55.2)</td></tr>
<tr><td>Yes</td><td>1378 (44.8)</td></tr>
</table>

Statistical methods used: dependent variable recoded: transformed into four answer alternatives (1 = very high or quite high; 2 = within half; 3 = quite low; 4 = very low); multinomial logistic regression; dependent variable: reference category =very low level of absenteeism; independent variable: reference category = absence of participation or absence of preventive management

Unadjusted/ adjusted estimates with precision (e.g. 95% confidence interval) for each outcome:

Tab.2: Association between inspection visits and the risk of absenteeism
<table>
<tr><th>Absenteeism</th><th>aOR(95%IC)[1]</th><th>p-Value</th></tr>
<tr><td>Quite Low</td><td>1.01 (0.83–1.23)</td><td>0.900</td></tr>
<tr><td>Within half</td><td>1.91 (1.56–2.33)</td><td><0.001</td></tr>
<tr><td>Very High or Quite High</td><td>2.02 (1.41–2.90)</td><td><0.001</td></tr>
<tr><td>Population</td><td>3.162</td><td></td></tr>
<tr><td>Number of valid cases and percentage</td><td>2916 (92.2%)</td><td></td></tr>
</table></td><td>Funding: Spanish Ministry of Education, Culture and Sports, grant number FPU2016 / 04591

Conflict of Interest stated: yes

Other comments:
- low response
- study population consists only of “the person who knows most about occupational safety and health” in opinion of company manager
- main outcome is participation</td></tr>
</table>

<table>
<tr><th>General</th><th>Study</th><th>Population</th><th>Exposure</th><th>Outcome</th><th>Results</th><th>Comments</th></tr>
<tr>
<td></td>
<td></td>
<td>Response: total (5+employees): Cooperation rate: 21%; response rate: 12%; contact rate: 74%, refusal rate: 28%[1]

companies at follow –up: NA

Loss-to-follow-up: NA

Work characteristics: all activity sectors (except sections T (Domestic activities) and U (Extraterritorial organisations) of the National Classification of Economic Activities (CNAE Rev.2)

*TNS Deutschland GmbH (TNS Infratest Sozialforschung): Second European survey of enterprises on new and emerging risks (ESENER-2): Report No. 8:Quality Report: https://search.gesis.org/research_data/ZA6865</td>
<td>t: 14 July -20 October 2014</td>
<td></td>
<td>[1]aOR= Odds ratios adjusted for other variables of participation, management and contral, 95% CI: confidence interval of 95%
(other variables in model: Passive participation, Consultative participation, Active participation, Prevention plans, Risks evaluation, Preventive planning, Collective representation, Economic sector, Workplace size)

Short summary of findings:
-Labour inspections are associated with higher levels of absenteeism</td>
<td></td>
</tr>
<tr>
<td>First Author, Year
Lee, 2019 #reference list

Extracted by: MS

Checked by:</td>
<td>Study name:-

Country: USA

Study design: quasi-experimental</td>
<td>Recruitment method/ Data source:
Data on safety inspection and fatality records from OSHA Integrated Management Information System (IMIS), Manufacturing facility data: Census of Manufactures (COM): linkage via SIC code: exclusion of plants <11 employees, receiving first inspection prior to study start (1987), receiving complaint or</td>
<td>Description of intervention:
Programmed inspections

Average annual programmed inspections

Study groups:</td>
<td>Outcome name:
Fatality rate

Outcome definition and assessment:
Average annual fatality rate per 10,000 workers for industries</td>
<td>Descriptive Statistics:
Tab.1: Summary statistics (panel A)
<table>
<tr><th>Variable</th><th>Description</th><th>Mean (SD)</th></tr>
<tr><td>Total Annual Inspections</td><td>Average annual inspections</td><td>7,682</td></tr>
<tr><td>Programmed Inspections</td><td>Average annual programmed inspections</td><td>3,365</td></tr>
<tr><td>Programmed Inspections with Violations</td><td>Average annual programmed inspections that result in at least one violation found</td><td>2,451</td></tr>
<tr><td>Violations</td><td>Average number of violations</td><td>4.64 (6.98)</td></tr>
<tr><td>Manuf. Fatality Rate (Fatrate)</td><td>Average annual fatality rate per 10,000 workers in all manufacturing industries</td><td>0.65 (21.23)</td></tr>
</table>
</td>
<td>Funding:
“research reported herein was conducted while Lee was a Special Sworn Status researcher of the US Census Bureau at the Triangle Census Research Data Center.”

Conflict of Interest stated: “Any opinions</td>
</tr>
</table>

<table>
<tr><th>General</th><th>Study</th><th>Population</th><th>Exposure</th><th>Outcome</th><th>Results</th><th>Comments</th></tr>
<tr>
<td></td>
<td>Time of Study: main analysis: 1987-1997</td>
<td>other non-random inspections, having fatality prior to being scheduled for programmed inspection

matching score to each possible plant-record combination in the Business Register and OSHA inspection data that is equal to the sum of the weighted number of field sequences for which the two records are a match

Matching criteria: unbalanced

invited companies: NA

companies at baseline: N plants =65,300

Response: NA

companies at follow –up: NA

Loss-to-follow-up: NA

Work characteristics: manufacturing plants</td>
<td>Control group=uninspected plants Treatment group=inspected plants

Time of measurement: 1988-1996</td>
<td>Time of measurement: 1987-1997</td>
<td>
<table>
<tr><td>Fatality Rate in OSHA Targeted Industries</td><td>Average annual fatality rate per 10,000 workers for industries targeted by OSHA for randomized inspections</td><td>3.05 (51.28)</td></tr>
</table>

Statistical methods used: regression

Unadjusted/ adjusted estimates with precision (e.g. 95% confidence interval) for each outcome:
Tab.4: Tests for effects of programmed inspections on fatality rates among related plants
<table>
<tr><th></th><th>Fatality rate
M1
Coeff (SE)</th><th>Fatality rate
M2
Coeff (SE)</th></tr>
<tr><td>Programmed inspection (PI)</td><td>-1.370*** (0.310)</td><td>-1.451***(0.308)</td></tr>
<tr><td>Related MSA</td><td>-0.009 (0.111)</td><td>-</td></tr>
<tr><td>Related firm</td><td>-</td><td>0.090 (0.082)</td></tr>
<tr><td>R^2</td><td>0.295</td><td>0.295</td></tr>
</table>
MSA= other plants in the same metropolitan statistical area (MSA), Statistical significance 1%, level represented by ***. N= 252,800 observations (65,300 plants. Models include plant and industry-by-state-by-year fixed effects. Standard errors clustered at plant level. Sample size rounded to nearest hundred due to Census Bureau confidentiality requirements.

Tab.A4: IV estimates of plant-level changes in risks in response to receiving an OSHA inspection
<table>
<tr><th></th><th>Fatality rate
Coeff (SE)</th></tr>
<tr><td>Programmed inspection</td><td>-1.338*** (0.338)</td></tr>
</table>
Also included in model: # employees, # production workers, cost of materials. PW productivity, single unit plant, turnover
Statistical significance 1%, level represented by ***. N=252,800 observations (65,300 plants). Industry-by-state-by-year fixed effects and plant-level fixed effects also included. Number of observations (number of plants) rounded to nearest 100 due to U.S. Census Bureau confidentiality requirements.

Short summary of findings:
- OSHA inspections reduce plant fatality rates by about 1.4 fatalities per 10,000 production workers -> average number of fatalities in OSHA-targeted industries is 3 deaths per 10,000 workers, plant-level fatality risks are decreased by approximately 45 percent after plants receive their first programmed OSHA inspection
- OSHA inspection reduces the number of fatalities
</td>
<td>and conclusions expressed herein are those of the authors and do not necessarily represent the views of the US Census Bureau”

Other comments:
- other main outcome: wages</td>
</tr>
</table>

<table>
<tr><th>Genera l</th><th>Study</th><th>Population</th><th>Exposure</th><th>Outcome</th><th>Results</th><th>Comments</th></tr>
<tr><td></td><td></td><td></td><td></td><td></td><td>- related plants in the same MSA or plants owned by the same parent company do not experience a change in fatality risks after a related plant receives an OSHA inspection</td><td></td></tr>
<tr>
<td>First Author, Year
Dahl, 2022
#284

Extracted by: MS

Checked by:</td>
<td>Study name:-

Country: Norway

Study design: before-after study (study 2+3)
Cross-sectional (study 1)

Time of Study: NR</td>
<td>Recruitment method/ Data source: data from regular health and safety inspections performed by the Norwegian Labour Inspection Authority (NLIA)
-> 3 studies

Matching criteria: NA

invited companies: NA

companies at baseline:
Study 1: n=29,224
Study 2: n=1119 companies
Study 3: n=378 companies

Response: NA

companies at follow –up: NA

Loss-to-follow-up: NA

Work characteristics:
Study 1:
<table>
<tr><th>Characteristics</th><th>N (%)</th></tr>
<tr><td>Wholesale and retail trade; repair of motor vehicles</td><td>6674 (22.8)</td></tr>
<tr><td>Construction</td><td>4555 (15.6)</td></tr>
<tr><td>Accommodation and food service activities</td><td>3722 (12.7)</td></tr>
<tr><td>Manufacturing</td><td>3072 (10.5)</td></tr>
<tr><td>Human health and social work activities</td><td>2492 (8.5)</td></tr>
</table></td>
<td>Description of intervention: inspections

Study groups: NA

Time of measurement:
2 times (study 2+3)
1 time (study 1)</td>
<td>Outcome name: Compliance

Outcome definition and assessment: inspectors applies predefined checklist; checklists differ according to inspection type and industry) -> in the 3 studies: 7 frequently checkpoints: 1. OSH training (“Has the employer undergone training in health and safety work?”); 2. Safety representative “Has the company chosen a safety representative?”), 3. Employment contract („Has a written employment contract been signed with the employees?”), 4. Health and safety skills (“Do</td>
<td>Descriptive Statistics:
Tab.2: Descriptive statistics
<table>
<tr><th>Topic</th><th>Study 1 Mean (SD)</th><th>Study 2 Mean (SD)</th><th>Study 3 Mean (SD)</th></tr>
<tr><td>OSH training</td><td>0.81 (0.39)</td><td>1.00 (0.00)</td><td>0.50 (0.50)</td></tr>
<tr><td>Safety representative</td><td>0.76 (0.43)</td><td>0.90 (0.29)</td><td>0.69 (0.46)</td></tr>
<tr><td>Employment contract</td><td>0.92 (0.27)</td><td>0.97 (0.18)</td><td>0.85 (0.35)</td></tr>
<tr><td>Health and safety skills</td><td>0.94 (0.24)</td><td>0.95 (0.22)</td><td>0.89 (0.32)</td></tr>
<tr><td>Working time overview</td><td>0.92 (0.27)</td><td>0.95 (0.21)</td><td>0.85 (0.35)</td></tr>
<tr><td>Hazard identification</td><td>0.70 (0.46)</td><td>0.80 (0.40)</td><td>0.68 (0.47)</td></tr>
<tr><td>Risk reduction measures</td><td>0.74 (0.44)</td><td>0.82 (0.38)</td><td>0.73 (0.44)</td></tr>
</table>
Statistical methods used: two-tailed paired t-test (study 2 + 3), Linear regression (study 1)

Unadjusted/ adjusted estimates with precision (e.g. 95% confidence interval) for each outcome:

Study 1: Effect of OSH training on compliance: B (unstandardized)=0.212, β =0.308, p < 0.001 (company size also included in model)

Study 2:
Tab.5: Scores for compliance index and checkpoints for companies with OSH training at T1 and T2
<table>
<tr><th>Topics</th><th>Pairs</th><th>Mean T1</th><th>Mean T2</th><th>Mean Diff.</th><th>% Diff.</th></tr>
<tr><td>Compliance index</td><td>1119</td><td>0.89</td><td>0.92</td><td>0.03</td><td>3.4%**</td></tr>
<tr><td>Safety representative</td><td>847</td><td>0.89</td><td>0.95</td><td>0.06</td><td>6.7%*</td></tr>
<tr><td>Employment contract</td><td>438</td><td>0.96</td><td>0.96</td><td>0.00</td><td>0.0%</td></tr>
<tr><td>Health and safety skills</td><td>587</td><td>0.96</td><td>0.95</td><td>-0.01</td><td>-1.0%</td></tr>
</table></td>
<td>Funding: no external funding

Conflict of Interest stated: yes

Other comments: -</td>
</tr>
</table>

General	Study	Population	Exposure	Outcome	Results	Comments
		(see below)		(see below)	(see below)	

Administrative and support service activities	1510 (5.2)
Transportation and storage	1325 (4.5)
Other	5874

Study 2:

Characteristics	N(%)
Wholesale and retail trade; repair of motor vehicles	181 (16.2)
Construction	194 (17.3)
Accommodation and food service activities	142 (12.7)
Manufacturing	215 (19.2)
Human health and social work activities	67 (6.0)
Administrative and support service activities	69 (6.2)
Transportation and storage	32 (2.9)
Other	219 (19.5)

Study3

Characteristics	N(%)
Wholesale and retail trade; repair of motor vehicles	40(21.2)
Construction	30 (15.9)
Accommodation and food service activities	57 (30.2)
Manufacturing	25 (13.2)
Human health and social work activities	5 (2.6)
Administrative and support service activities	3 (1.6)
Transportation and storage	6 (3.2)

employees assigned to lead others have the necessary skills such that the consideration of safety and health is taken care of within the manager's area of responsibility?"), 5. Working time overview („Does the employer have a continuous overview of how much the individual employee works?"), 6. Hazard identification ("Has the employer identified the hazards and problems the employees may be exposed to in the business and, on this basis, assessed the risk of injury or danger to workers' health and safety?"), and 7. Risk reduction measures („Has the employer taken the

Working time overview	273	0.92	0.95	0.03	3.3%
Hazard identification	347	0.80	0.83	0.03	3.8%
Risk reduction measures	324	0.83	0.82	-0.01	-1.2%

* p < 0.001. ** t-test: df = 1118, t = 3.310, p < 0.001 (effect size: Cohen's d = 0.10),

Study 3:
Tab. 6: Scores for compliance index and checkpoints for companies with managers that lack OSH training at T1 but have OSH training at T2

Topics	Pairs	Mean T1	Mean T2	Mean Diff.	% Diff.
Compliance index	189	0.67	0.87	0.20	29.9%***
Safety representative	115	0.64	0.91	0.27	21.2%*
Employment contract	111	0.82	0.91	0.09	11.0%**
Health and safety skills	41	0.78	0.98	0.20	25.6%**
Working time overview	71	0.82	0.86	0.04	4.9%
Hazard identification	31	0.77	0.84	0.07	9.1%
Risk reduction measures	30	0.83	0.87	0.04	4.8%

* p < 0.001. ** p < 0.05. *** t-test: df = 188, t = 6.284, p < 0.001 (effect size: Cohen's d = 0.46)

Short summary of findings:
- study 1: OSH training is positively and significantly related to the compliance index
- study 2: companies with managers with mandatory health and safety training at both T1 (first inspection) and T2 (second inspection) scored significantly higher on the compliance index at T2 compared to T1 (single checkpoints only "safety representative" improves significantly)
- study 3: companies led by managers with no mandatory OSH training at T1 (first inspection) but at T2 scored significantly higher on the compliance index at T2 (second inspection) compared to T1
- OSH training positively linked to OSH compliance

<table>
<tr><th>General</th><th>Study</th><th>Population</th><th>Exposure</th><th>Outcome</th><th>Results</th><th>Comments</th></tr>
<tr><td></td><td></td><td><table><tr><td>Other</td><td>23 (12.1)</td></tr></table></td><td></td><td>necessary measures and/or plans that describe measures to remove or reduce hazards and problems at work?”) response: “yes” (1), “no” (0) (no triggers a formal order) ->calculation of a compliance index from checkpoint #2 to #7 (if not all 6 checkpoints controlled-> value of actually controlled checkpoints)

-Compliance index = checkpoints satisfied / checkpoints controlled

Time of measurement:
2 times (study 2+3)
1 time (study 1)</td><td></td><td></td></tr>
<tr><td>First Author, Year
Agnesi, 2016
#Andersen review</td><td>Study name: -

Country:
Italy</td><td>Recruitment method/ Data source:
- National Institute for the Insurance of Work-Related Injuries (INAIL): injury data National Health</td><td>Description of intervention:
Programmed inspection Inspection after injury</td><td>Outcome name:
Injury rates

Outcome definition and assessment:</td><td>Descriptive Statistics:
Tab.1: Descriptive summary: employment<table><tr><th>Intervention type</th><th>Mean number of employees</th><th>Person-years</th></tr><tr><td>Inspection after injury</td><td>41.1</td><td>98,295</td></tr><tr><td>Programmed inspection</td><td>37.4</td><td>60,701</td></tr><tr><td>Reference group</td><td>30.0</td><td>878,181</td></tr></table></td><td>Funding:
Italian Ministry of Health and the Veneto Region (RF-2009-1493881)</td></tr>
</table>

<table>
<tr><th>General</th><th>Study</th><th>Population</th><th>Exposure</th><th>Outcome</th><th>Results</th><th>Comments</th></tr>
<tr>
<td>Extracted by: MS

Checked by:</td>
<td>Study design: Before-after study with control group

Time of Study: 2001-2007</td>
<td>- System Service of Prevention, Health and Safety at Work (SPISAL) archives: information on intervention
- manufacturing industry with >10 employees; exclusion of construction companies
- Veneto region (North-Eastern Italy)

Matching criteria: no

invited companies: NA

companies at baseline:
intervention group: n=795
Control group: n=4186

Response: NA

companies at follow –up: NA

Loss-to-follow-up: NA

Work characteristics: manufacturing industry</td>
<td>Study groups:
<u>Intervention group</u>: receiving SPISAL inspection: inspection after injury: n=342; programmed inspection: n=232; multiple interventions in 186/ 795 firms
<u>Control group:</u> firms in INAIL database

Time of measurement: 2003-2005</td>
<td>INAIL definition of injury = 3 days of absence from work

Injury rates = either overall injuries or major injuries (fatalities; lost workdays >30 days; degree of permanent disability >0) / average annual number of employees
-observation period divided into time points: injury rates calculated for each time point

Time of measurement: 2001-2007</td>
<td>Tab.1: Descriptive summary: injuries from 2001 to 2007
<table>
<tr><th></th><th>Overall</th><th>Minor</th><th>Major</th></tr>
<tr><td>Inspection after injury</td><td>6417</td><td>5083</td><td>1334</td></tr>
<tr><td>Programmed inspection</td><td>3267</td><td>2661</td><td>606</td></tr>
<tr><td>Reference group</td><td>38664</td><td>31450</td><td>7214</td></tr>
</table>

Tab.1: Descriptive summary: injury rates (x 1000 person-years) from 2001 to 2007
<table>
<tr><th></th><th>Overall</th><th>Minor</th><th>Major</th></tr>
<tr><td>Inspection after injury</td><td>65.3</td><td>51.7</td><td>13.6</td></tr>
<tr><td>Programmed inspection</td><td>53.8</td><td>43.8</td><td>10.0</td></tr>
<tr><td>Reference group</td><td>44.0</td><td>35.8</td><td>8.2</td></tr>
</table>

Statistical methods used: interrupted time series analysis -> Poisson regression

Unadjusted/ adjusted estimates with precision (e.g. 95% confidence interval) for each outcome:

Tab.2: Effect of inspection after injury (A), programmed inspection (B) on total injuries and major injuries
<table>
<tr><th></th><th>Total injuries
RR (95% CI)</th><th>Minor injuries
RR (95% CI)</th></tr>
<tr><td colspan="3">A—inspection after injury</td></tr>
<tr><td>Baseline time trend</td><td>1.00 (0.99-1.02)</td><td>1.03 (1.00-1.05)</td></tr>
<tr><td>Peak preintervention</td><td>1.23 (1.04-1.44)</td><td>2.48 (2.00-3.01)</td></tr>
<tr><td>Effect of intervention*</td><td>0.76 (0.65-0.90)</td><td>0.64 (0.50-0.83)</td></tr>
<tr><td>Change in time trend</td><td>0.99 (0.97-1.01)</td><td>0.99 (0.96-1.01)</td></tr>
<tr><td colspan="3">B—programmed inspection</td></tr>
<tr><td>Baseline time trend</td><td>0.98 (0.97-1.00)</td><td>0.98 (0.96-1.01)</td></tr>
<tr><td>Peak preintervention</td><td>0.98 (0.80-1.20)</td><td>1.20 (0.83-1.71)</td></tr>
<tr><td>Effect of intervention*</td><td>0.96 (0.81-1.14)</td><td>1.14 (0.81-1.61)</td></tr>
<tr><td>Change in time trend</td><td>1.02 (0.99-1.04)</td><td>1.01 (0.97-1.05)</td></tr>
</table>

Tab. 3: Intervention after injury versus no intervention
<table>
<tr><th>Models</th><th>Total injuries</th><th>Major injuries</th></tr>
<tr><td>Unadjusted</td><td>0.79 (0.67- 0.94)</td><td>0.62 (0.47-0.81)</td></tr>
<tr><td>Adjusted*</td><td>0.84 (0.71-0.99)</td><td>-</td></tr>
<tr><td>Matched analysis**</td><td>0.83 (0.70-0.97)</td><td>0.65 (0.47-0.89)</td></tr>
</table>
(interaction term group × effect (change in rates in the intervention group after the inspection, with respect to the reference group)
* adjusted for industrial sector and Local Health Units
** analysis conducted on groups matched by industrial sector and Local Health Unit</td>
<td>Conflict of Interest stated: yes

Other comments: -</td>
</tr>
</table>

<table>
<tr><th>General</th><th>Study</th><th>Population</th><th>Exposure</th><th>Outcome</th><th>Results</th><th>Comments</th></tr>
<tr><td></td><td></td><td></td><td></td><td></td><td>Short summary of findings:
Significant effect of inspection after injury but not for programmed inspection: Inspection after injury reduced by 24%, all injuries, and by 36% (fatalities, lost workdays >30, degree of permanent disability >0); effects were acute and persisted for 2 years</td><td></td></tr>
<tr><td>First Author, Year
Farina, 2016
#Andersen review

Extracted by: MS

Checked by:</td><td></td><td></td><td>Description of intervention: inspection

<u>- comprehensive:</u> complete intervention covering potentially high-risk areas of plant,
<u>- partial:</u> intervention limited to certain potentially hazardous areas, operations, conditions or practices

Time of measurement:
At least one inspection from 2003 to 2005; none</td><td>Outcome name: Injury

Outcome definition and assessment: Total & serious first injuries occurring after inspection (serious injury = deaths, injuries involving permanent disability or more than 30 days of absence from work)

Time of measurement: Not specified</td><td>Descriptive Statistics:
Tab.1: Distribution (%) of firm and inspection characteristics by completeness of inspection
<table>
<tr><th></th><th>Partial inspection (n=331)</th><th>Comprehensive inspection (n=243)</th><th>Total (574)</th></tr>
<tr><td>Firm characteristics</td><td></td><td></td><td></td></tr>
<tr><td>Firm size</td><td></td><td></td><td></td></tr>
<tr><td>10-30 employees</td><td>175 (30%)</td><td>160 (66%)</td><td>335 (58%)</td></tr>
<tr><td>>30 employees</td><td>156 (47%)</td><td>83 (34%)</td><td>239 (42%)</td></tr>
<tr><td>Class of incidence</td><td></td><td></td><td></td></tr>
<tr><td>Class 1</td><td>45 (14%)</td><td>26 (11%)</td><td>71 (13%)</td></tr>
<tr><td>Class 2</td><td>117 (34%)</td><td>79 (33%)</td><td>196 (34%)</td></tr>
<tr><td>Class 3</td><td>48 (15%)</td><td>40 (16%)</td><td>88 (15%)</td></tr>
<tr><td>Class 4</td><td>121 (37%)</td><td>98 (40%)</td><td>219 (38%)</td></tr>
<tr><td>Serious injury rate 2001-2002 (mean)</td><td>1.41</td><td>1.23</td><td></td></tr>
<tr><td>Inspection characteristics</td><td></td><td></td><td></td></tr>
<tr><td>Number of visits</td><td></td><td></td><td></td></tr>
<tr><td>1</td><td>259 (78%)</td><td>173 (71%)</td><td>432 (75%)</td></tr>
<tr><td>>1</td><td>72 (22%)</td><td>70 (29%)</td><td>142 (25%)</td></tr>
<tr><td>Number of officers</td><td></td><td></td><td></td></tr>
<tr><td>1</td><td>129 (39%)</td><td>50 (21%)</td><td>179 (31%)</td></tr>
<tr><td>>1</td><td>202 (61%)</td><td>193 (79%)</td><td>395 (69%)</td></tr>
<tr><td>Type of inspection</td><td></td><td></td><td></td></tr>
<tr><td>Planned</td><td>31 (9%)</td><td>201 (83%)</td><td>232 (40%)</td></tr>
<tr><td>After injury</td><td>300 (91%)</td><td>42 (17%)</td><td>342 (60%)</td></tr>
<tr><td>Presence of violations</td><td></td><td></td><td></td></tr>
<tr><td>Yes</td><td>166 (50%)</td><td>203 (84%)</td><td>369 (64%)</td></tr>
<tr><td>No</td><td>165 (50%)</td><td>40 (16%)</td><td>205 (36%)</td></tr>
<tr><td>Total</td><td>331 (58%)</td><td>243 (42%)</td><td>574</td></tr>
</table>
class of incidence: defined on basis of incidence of injury in Veneto in 2003 of the associated economic activity: Class 1 = lowest injury rate; Class 4= highest injury rate)

- median follow-up time: 167.5 days (152 after partial inspection, 202 after comprehensive inspection),
- median follow-up time for serious injuries: 682 days (592 after partial inspection, 743 after comprehensive inspection)</td><td>Conflict of Interest stated: yes

Other comments: -</td></tr>
</table>

<table>
<tr><th>General</th><th>Study</th><th>Population</th><th>Exposure</th><th>Outcome</th><th>Results</th><th>Comments</th></tr>
<tr><td></td><td></td><td></td><td>during 2 years prior to first inspection and 2 years after the last inspection</td><td></td><td>

Statistical methods used: Multivariate Cox Models

Unadjusted/ adjusted estimates with precision (e.g. 95% confidence interval) for each outcome:

Tab. 2: Hazard ratio (p-value) of Multivariate Cox Models for Total Injuries stratified by firm size

<table>
<tr><td></td><td colspan="2">Total injuries</td></tr>
<tr><td>Firm size</td><td>10-30</td><td>>30</td></tr>
<tr><td>Model 1</td><td>Hazard ratio (p-value)</td><td>Hazard ratio (p-value)</td></tr>
<tr><td>Completeness (vs. partial)</td><td>0.74 (0.018)</td><td>1.15 (0.344)</td></tr>
<tr><td>Number of visits (>1 vs. 1)</td><td>1.11 (0.487)</td><td>0.97 (0.854)</td></tr>
<tr><td>Number of officers (>1 vs. 1)</td><td>1.01 (0.950)</td><td>0.90 (0.488)</td></tr>
<tr><td>Class of incidence (ref. class 1)</td><td></td><td></td></tr>
<tr><td>Class 2</td><td>1.21 (0.416)</td><td>1.30 (0.182)</td></tr>
<tr><td>Class 3</td><td>1.45 (0.125)</td><td>0.98 (0.960)</td></tr>
<tr><td>Class 4</td><td>1.94 (0.003)</td><td>1.30 (0.211)</td></tr>
<tr><td>Serious injury rate 2001-2002</td><td>1.04 (0.132)</td><td>1.19 (<0.001)</td></tr>
<tr><td>Model2: M1 + type of inspection</td><td></td><td></td></tr>
<tr><td>Completeness (vs. partial)</td><td>0.77 (0.203)</td><td>1.28 (0.208)</td></tr>
<tr><td>Inspection type (PI vs. AI)*</td><td>0.95 (0.810)</td><td>0.85 (0.405)</td></tr>
<tr><td>Model3. M1 + violations</td><td></td><td></td></tr>
<tr><td>Completeness (vs. partial)</td><td>0.80 (0.083)</td><td>1.24 (0.168)</td></tr>
<tr><td>Violations (yes/ no)</td><td>0.77 (0.090)</td><td>0.78 (0.104)</td></tr>
</table>

PI = programmed inspection, AI = accident inspection

Tab. 2: Hazard ratio (p-value) of Multivariate Cox Models for serious Injuries stratified by firm size

<table>
<tr><td></td><td colspan="2">Serious injuries</td></tr>
<tr><td>Firm size</td><td>10-30</td><td>>30</td></tr>
<tr><td>Model 1</td><td>Hazard ratio (p-value)</td><td>Hazard ratio (p-value)</td></tr>
<tr><td>Completeness (vs. partial)</td><td>0.72 (0.063)</td><td>0.84 (0.307)</td></tr>
<tr><td>Number of visits (>1 vs. 1)</td><td>1.11 (0.605)</td><td>0.86 (0.418)</td></tr>
<tr><td>Number of officers (>1 vs. 1)</td><td>0.94 (0.719)</td><td>0.99 (0.987)</td></tr>
<tr><td>Class of incidence (ref. class 1)</td><td></td><td></td></tr>
<tr><td>Class 2</td><td>1.64 (0.181)</td><td>1.14 (0.570)</td></tr>
<tr><td>Class 3</td><td>1.15 (0.744)</td><td>1.01 (0.972)</td></tr>
<tr><td>Class 4</td><td>2.16 (0.031)</td><td>1.73 (0.027)</td></tr>
<tr><td>Serious injury rate 2001-2002</td><td>1.04 (0.211)</td><td>1.18 (0.003)</td></tr>
<tr><td>Model2: M1 + type of inspection</td><td></td><td></td></tr>
<tr><td>Completeness (vs. partial)</td><td>0.74 (0.283)</td><td>0.84 (0.424)</td></tr>
<tr><td>Inspection type (PI vs. AI)*</td><td>0.97 (0.914)</td><td>0.99 (0.999)</td></tr>
</table>

</td><td></td></tr>
</table>

<table>
<tr><th>General</th><th>Study</th><th>Population</th><th>Exposure</th><th>Outcome</th><th>Results</th><th>Comments</th></tr>
<tr><td></td><td></td><td></td><td></td><td></td><td>
<table>
<tr><td>Model3. M1 + violations</td><td></td><td></td></tr>
<tr><td>Completeness (vs. partial)</td><td>0.73 (0.081)</td><td>0.87 (0.423)</td></tr>
<tr><td>Violations (yes/ no)</td><td>0.98 (0.911)</td><td>0.87 (0.581)</td></tr>
</table>
PI = programmed inspection, AI = accident inspection

Short summary of findings:
- smaller companies with complete inspection have lower risk for total and serious injuries (no effect for larger companies)
</td><td></td></tr>
<tr><td>First Author, Year
Kolenprat, 2023 #graue Literatur

Extracted by: MS

Checked by:</td>
<td>Study name:

Country: Austria

Study design: Before-after study

Time of Study: March 2021 – May 2022</td>
<td>Recruitment method/ Data source:
-number of companies to be inspected per labour inspectorate calculated proportionally according to distribution of opencast mines in supervisory districts of the labour inspectorate -> selection of workplaces carried out by labour inspectorates themselves -> selection of workplaces not based on sampling and specification of a list of workplaces to be visited -> no conclusions about any distributions

Matching criteria: NA

invited companies: NA

companies at baseline: N=286

Response: NA</td>
<td>Description of intervention: Inspection for mineral dust exposure

Study groups: 1 intervention group

Time of measurement:
Baseline: March- July 2021
Follow-up: March- May 2022</td>
<td>Outcome name: Compliance

Outcome definition and assessment:
1.Are health & safety documents at time of inspection available?
2. Do health & safety documents contain information on composition of mineral raw materials & the overburden, in particular with regard to quartz or asbestos minerals?
3. Do health and safety documents submitted address & record expected exposure of workers to released mineral dusts?</td>
<td>Descriptive Statistics: Percentage of workplaces

<table>
<tr><th></th><th>Baseline (n=287)</th><th>Follow-up (n=238 of which n=217 participated in baseline)</th></tr>
<tr><td>1.Availability of health & safety documents at workplace</td><td>80%</td><td>96%</td></tr>
<tr><td>2.Mineralogical composition of the raw materials and overburden was at least partially known</td><td>62%</td><td>84%</td></tr>
<tr><td>3.Information on expected exposure of workers to mineral dusts released could be obtained</td><td>46%
(in 14% no information on mineral dusts)</td><td>81%
(in 0.5% no information on mineral dusts</td></tr>
<tr><td>technical & organizational measures to minimize workers' exposure to mineral dusts released</td><td>30%</td><td>87%</td></tr>
</table>
</td>
<td>Funding: NA (study performed by Federal Ministry of Labour and Economics (BMAW))

Conflict of Interest stated: NA

Other comments:
- final report of a survey conducted by authorities</td></tr>
</table>

<table>
<tr><th>General</th><th>Study</th><th>Population</th><th>Exposure</th><th>Outcome</th><th>Results</th><th>Comments</th></tr>
<tr><td></td><td></td><td># companies at follow –up: N=238 (of which n=217 participated in first survey)

Loss-to-follow-up: NA

Work characteristics: focus on opencast mining and its processing plants</td><td></td><td>4. Are technical & organizational measures to minimize workers' exposure to released mineral dusts addressed & recorded in the submitted health and safety documents?

Time of measurement:
Baseline: March-July 2021
Follow-up: March-May 2022</td><td>Statistical methods used: no

Unadjusted/ adjusted estimates with precision (e.g. 95% confidence interval) for each outcome: only descriptive analysis

Short summary of findings:
-significant improvement in compliance with legal requirements in all aspects
- in half of workplaces protocols for dust concentration could be presented to labour inspector</td><td></td></tr>
<tr><td>First Author, Year
Brückner et al. 2018

#graue Literatur

Extracted by: MS

Checked by:</td><td>Study name:
“GDA-Wirkungsprojekt”

Country: Germany

Study design: Retrospective before-after study

Time of Study: January</td><td>Recruitment method/ Data source:
Companies with 10-49 and 50-249 employees from western and eastern federal states (Brandenburg, Hesse, Mecklenburg-Western Pomerania & North Rhine-Westphalia)
Recruitment via company lists exchanged between federal states and accident insurance organisation
Planned sample size: N=120 (3 groups each: n=40)

Matching criteria: triples matched according to site group, size, industrial sector and region</td><td>Description of intervention:
Inspection (tandem of state representative and an accident insurance organisation)
- t_0 = inspection
- t_1 = after inspection according to GDA program
-no intervention</td><td>Outcome name: Compliance

Outcome definition and assessment:
- t_1 and t_2: identical assessment
-assessment announced to companies beforehand by letters from state authorities
- interview questionnaire on implementation and quality of occupational safety (1x for</td><td>Descriptive Statistics:

Tab. 3.1-3.3. Descriptive summary
<table>
<tr><th></th><th>“AP Orga”</th><th>GDA guideline</th><th>Control group</th></tr>
<tr><td colspan="4">Company size</td></tr>
<tr><td>10-49 employees</td><td>14</td><td>31</td><td>25</td></tr>
<tr><td>>50 employees</td><td>18 (2 companies with 250-1.000)</td><td>19
(4 companies >250, 2 companies >1.000)</td><td>9 (2 companies with 250-1.000)</td></tr>
<tr><td colspan="4">Branch</td></tr>
<tr><td>manufacturing</td><td>19</td><td>24</td><td>21</td></tr>
<tr><td>service</td><td>13</td><td>26</td><td>13</td></tr>
<tr><td colspan="4">Region</td></tr>
<tr><td>West</td><td>18</td><td>22</td><td>20</td></tr>
<tr><td>East</td><td>14</td><td>28</td><td>14</td></tr>
</table></td><td>Funding: NA

Conflict of Interest stated: NA

Other comments:
- no information on occupational safety level at t_0 in all participating companies
- no information on statistical analysis
-no data for results reported
- prior testing of questionnaires from inspectorates and prevention services</td></tr>
</table>

General	Study	Population	Exposure	Outcome	Results	Comments
	2017 – April 2018	**# invited companies:** NA<br><br>**# companies at baseline:** N=116<br>(groups:<br>-intervention group1: “AP Orga Betriebe”: n=32<br>-intervention group 2: GDA guideline (GDA-Leitlinien-Betriebe“): n=50<br>Control group (no intervention): n=34<br><br>**Response:** NR<br><br>**# companies at follow –up:** N=113 (company did not exist anymore or no appointment could be scheduled)<br><br>**Loss-to-follow-up:** NA<br><br>**Work characteristics:** manufacturing & service sector	between t_1 and t_2 (no information on occupational safety level at t_0)<br><br>**Study groups:** Intervention group 1: firms with GDA-work program (“AP Orga Betriebe”), Intervention group 2: firms with inspection according to GDA guideline Control group: no inspection since 2011<br><br>**Time of measurement:** t_0 =before study (no detailed information)	interview) with the categories 1. Formal occupational health and safety situation, 2. Engagement of management, 3. Participation, 4. Social climate, 5. Consistent approach and 6. Culture of failure<br>- questionnaire on implementation and quality of occupational safety: same questions as interview response categories: different options (yes/ no,..), free text, 5-point scale (1 = not present; 2= planned, 3=started, 4=partially implemented, 5=fully implemented), 4-pont scale (1=true; 2=somewhat true, 3= somewhat not true, 4=untrue)<br>- questionnaire for inspection of company	Bias<br>- after baseline, letters of findings of visits sent to 26 companies<br>- contacts between first and second survey with inspectorates or prevention services (n=6)<br>- questionnaire were sent prior to visit (n=5)<br>(changes account for in data analysis)<br><br>Changes in companies between first and second visit<br>- change of management (n=11)<br>- changes in work processes such as new technologies, etc (n=42)<br>Organizational changes such as expansion, etc (n=30)<br>(changes account for in data analysis)<br><br>**Statistical methods used**: not described<br><br>**Unadjusted/ adjusted estimates with precision (e.g. 95% confidence interval) for each outcome:**<br>No data reported<br><br>**Short summary of findings:**<br>- “AP Orga Betriebe“: no differences in any outcome between baseline and follow-up<br>- “GDA guideline group”: significant differences in “Formal occupational health and safety situation”,“Participation” and “Regular supervision: organization of occupational health and safety processes” t1 and t2.<br>- group of non-visited companies: significant changes for “Formal safety situation” and for ‘first aid and emergency measures’.	

<table>
<tr><th>General</th><th>Study</th><th>Population</th><th>Exposure</th><th>Outcome</th><th>Results</th><th>Comments</th></tr>
<tr><td></td><td></td><td></td><td></td><td>- recording of key company data: summarizes questions on structural features and change process
- log sheet for company visit: questions about the duration, conversation and atmosphere

Time of measurement: t_1 and t_2 separated by one year
t_1= January - April 2017
t_1= January - April 2018</td><td></td><td></td></tr>
<tr><td>First Author, Year
Lamers & Baars, 2018
#graue Literatur

Gründler & Schulz, 2018
#graue Literatur</td><td>Study name:
“GDA-Wirkungsprojekt” workpage for prevention of musculoskeletal disorders (MSD)

Country:
Germany</td><td>Recruitment method/ Data source:
Invited companies chosen according to key industry for MSD and company size for baseline inspection (at least 10% companies with ≤ 20 employees, max 10% companies with ≥250).
For follow-up, companies selected using a “score” that was based on 5 variables -> identification of companies that did not achieve adequate results in baseline</td><td>Description of intervention: inspection
Evaluation of prevention program for MSD
Program included seminars, symposiums, online tools for employees, qualification</td><td>Outcome name:
Use and awareness of GDA products

Outcome definition and assessment:
Outcome assessed with questionnaire developed for MSD work package

Time of measurement:
Baseline: QIII 2014–Q IV 2017</td><td>Descriptive Statistics:
Of 13,609 companies 12,109 could be included in data analysis.
Data availability for baseline inspection (n=11,955) and for follow-up (n=1,292).
(Gründler & Schulz: of 13,751 data sets 12,109 from baseline inspection and 1,110 for follow-up inspection)

Tab. 3.3: Distribution of companies according to size at baseline<table><tr><td>Company size</td><td>Number of companies</td></tr><tr><td>≤ 20 employees</td><td>3,889</td></tr><tr><td>21-249 employees</td><td>6,861</td></tr><tr><td>>250 employees</td><td>1,359</td></tr></table>
Statistical methods used: no models used</td><td>Funding: NA

Conflict of Interest stated: no

Other comments:
- Lamers & Baarss and Gründler & Schulz: identical reports
- only descriptive statistics used</td></tr>
</table>

<table>
<tr><th>General</th><th>Study</th><th>Population</th><th>Exposure</th><th>Outcome</th><th>Results</th><th>Comments</th></tr>
<tr>
<td>Extracted by: MS

Checked by:</td>
<td>(all states included)

Study design: randomized before-after study with control group

Time of Study: September 1st to December 2017</td>
<td>inspection (score of <14 of 23 points)

Matching criteria: NA

invited companies: NA

companies at baseline: Number of inspections at baseline: n=13,869

Response: NA

companies at follow –up: n=1,386

Loss-to-follow-up: NA

Work characteristics: key industries for risk of musculoskeletal diseases:
- waste disposal
- construction
- forestry
- healthcare/welfare
- trade and logistics
- maintenance and repair of motor vehicles
- workplace kitchen
- metal/plastic production and processing
- food production
- cleaning
- pre-school childcare</td>
<td>and training courses

Study groups: 1 intervention group

Time of measurement: Baseline: QIII 2014 – Q IV 2017
Follow-up: QIII-QIV 2016 and QII-IV 2017</td>
<td>Follow-up: QIII-QIV 2016 and QII-IV 2017</td>
<td>Unadjusted/ adjusted estimates with precision (e.g. 95% confidence interval) for each outcome

Fig. 4.0: Use of key indicator method in companies
<table>
<tr><td>key indicator method</td><td>% companies at baseline</td><td>% companies at follow-up</td></tr>
<tr><td>Lift & carry</td><td>26% (n=969)</td><td>49% (n=970)</td></tr>
<tr><td>Pull & push</td><td>16% (n=739)</td><td>33% (n=726)</td></tr>
<tr><td>manual work processes</td><td>15% (n=317)</td><td>27% (n=271)</td></tr>
</table>

Fig. 4.1: Performance of risk assessment
<table>
<tr><td>Risk assessment</td><td>Baseline (n=1,107)</td><td>Follow-up (n=1,108)</td></tr>
<tr><td>Implementation of risk assessment appropriate</td><td>18%</td><td>59%</td></tr>
<tr><td>Implementation of risk assessment not appropriate</td><td>43%</td><td>31%</td></tr>
<tr><td>No risk assessment performed</td><td>39%</td><td>10%</td></tr>
</table>

Fig. 4.3: Consideration of psychological load with regard to MSD:
Baseline inspection (n=1,110): 25%
Follow-up (n=1,110): 46%

Fig. 4.4: Proportion of groups involved in the risk assessment
<table>
<tr><td>Groups</td><td>Baseline</td><td>Follow-up</td></tr>
<tr><td>Employer/responsible manager</td><td>58% (n=442)</td><td>78% (n=452)</td></tr>
<tr><td>Occupational safety expert</td><td>67% (n=442)</td><td>85% (n=452)</td></tr>
<tr><td>Works council/staff council/employee representation</td><td>47% (n=292)</td><td>56% (n=308)</td></tr>
<tr><td>Company physician</td><td>39% (n=441)</td><td>58% (n=452)</td></tr>
<tr><td>Safety officer</td><td>36% (n=442)</td><td>50% (n=452)</td></tr>
</table></td>
<td></td>
</tr>
</table>

General	Study	Population	Exposure	Outcome	Results	Comments
					(see below)	

Employees	31% (n=442)	42% (n=452)
Other representatives or technical experts	10% (n=441)	16% (n=452)

Fig. 4.5: Ergonomic design of workplaces

	Baseline (n=1,107)	Follow-up (n=1,108)
Ergonomically optimized	15%	34%
Minimum standards fulfilled	57%	57%
Minimum standards partly fulfilled	26%	9%
Ergonomically unfavourably designed	2%	0%

Fig. 4.5: Ergonomic design of work processes

	Baseline (n=1,107)	Follow-up (n=1,108)
Ergonomically optimized	15%	33%
Minimum standards fulfilled	58%	57%
Minimum standards partly fulfilled	25%	9%
Ergonomically unfavourably designed	3%	1%

Fig 4.8: Comparison of occupational health and safety measures, participation processes and leadership skills between first and second inspection (mean values; 3: yes, completely, always, 2: partially, mostly, frequently; 1: rarely, little; 0: no, never, not at all)

	Baseline (n=1,107)	Follow-up (n=1,108)
Employee instruction	1.55 (n=1,106)	2.21 (n=1,107)
Instructions directly at workplace	1.39 (n=1,106)	2.00 (n=1106)
Participation of employees	1.68 (n=1,106)	2.14 (n=1,107)

General	Study	Population	Exposure	Outcome	Results	Comments

Regulations on handling loads	1.80 (n=968)	2.37 (n=953)
Training of managers	1.16 (n=1,104)	1.77 (n=1,107)
Managers support preventative behaviour	1.55 (n=1,106)	2.11 (n=1,107)

Fig.: 4.9: Compulsory and optional occupational health care for activities involving vibrations

	Baseline (n=424)	Follow-up (n=377)
arranged or offered within the time limit	24%	54%
before commencing work but subsequently not arranged in good time or not offered in writing or not offered before commencing work	26%	27%
only arranged after commencement of the activity or only partially or not offered in due time	18%	8%
not arranged or offered	32%	11%

Fig.: 4.10: Occupational health care for activities involving load handling, repetitive manual activities or forced postures

	Baseline (n=424)	Follow-up (n=377)
arranged or offered within the time limit	19%	43%
before commencing work but subsequently not arranged in good time or not offered in writing or not offered before commencing work	20%	29%
only arranged after commencement of the activity or	15%	9%

General	Study	Population	Exposure	Outcome	Results	Comments

only partially or not offered in due time		
not arranged or offered	46%	19%

Fig. 4.11: Embedding prevention programs in a company health management concept

Embedding prevention programs	Baseline (n=542)	Follow-up (n=524)
Yes	17%	35%
No	83%	65%

Fig. 4.12: Preventive measures offered by companies

Preventive measures offered	Baseline (n=542)	Follow-up (n=524)
Continuously	13%	25%
Sporadically	29%	31%
Never	58%	44%

Fig. 4.13: Used preventive measures by employees

Preventive measures used	Baseline (n=536)	Follow-up (n=588)
High	8%	17%
Medium	32%	46%
Low	60%	38%

Fig. 4.14: Use of ergonomic work equipment and aids by employees

Use of ergonomic work equipment and aids	Baseline (n=1,070)	Follow-up (n=1,071)
Completely	26%	42%
Predominantly	55%	51%
Little	16%	6%
Not at all	3%	1%

Fig. 4.15: Use of occupational health care offered by employees

Occupational health care	Baseline (n=624)	Follow-up (n=1,071)
Used by all	15%	30%

General	Study	Population	Exposure	Outcome	Results	Comments
					Used by most 29% 40%<br>Used by little 22% 15%<br>Not used 35% 14%<br><br>**Short summary of findings:**<br>- improvement in awareness and use of GDA products for prevention of MSD<br>- increase in compliance at follow-up<br>"The inspection data clearly shows that the companies have not yet satisfactorily implemented MSD prevention and that key areas still need to be addressed in the future." (Lamers & Baars)	

Abbreviations: NA=not applicable, NR= not reported
